# Quantitative Persulfide Site Identification (qPerS-SID) Reveals Protein Targets of H_2_S Releasing Donors in Mammalian Cells

**DOI:** 10.1038/srep29808

**Published:** 2016-07-14

**Authors:** Sebastian Longen, Florian Richter, Yvette Köhler, Ilka Wittig, Karl-Friedrich Beck, Josef Pfeilschifter

**Affiliations:** 1 Pharmazentrum Frankfurt/ZAFES, Universitätsklinikum Frankfurt, Frankfurt am Main, Germany; 2 Functional Proteomics, SFB 815 Core Unit, Goethe University Frankfurt, Frankfurt, Germany

## Abstract

H_2_S is an important signalling molecule involved in diverse biological processes. It mediates the formation of cysteine persulfides (R-S-SH), which affect the activity of target proteins. Like thiols, persulfides show reactivity towards electrophiles and behave similarly to other cysteine modifications in a biotin switch assay. In this manuscript, we report on qPerS-SID a mass spectrometry-based method allowing the isolation of persulfide containing peptides in the mammalian proteome. With this method, we demonstrated that H_2_S donors differ in their efficacy to induce persulfides in HEK293 cells. Furthermore, data analysis revealed that persulfide formation affects all subcellular compartments and various cellular processes. Negatively charged amino acids appeared more frequently adjacent to cysteines forming persulfides. We confirmed our proteomic data using pyruvate kinase M2 as a model protein and showed that several cysteine residues are prone to persulfide formation finally leading to its inactivation. Taken together, the site-specific identification of persulfides on a proteome scale can help to identify target proteins involved in H_2_S signalling and enlightens the biology of H_2_S and its releasing agents.

During the last decades, short-lived and reputedly toxic molecules such as nitric oxide (NO) and reactive oxygen species (ROS) have been identified as important endogenously synthesised signalling molecules affecting diverse cellular processes (for review see ref. 1). Recently, an additional signalling molecule has been identified heading spotlight: hydrogen sulfide (H_2_S). H_2_S attracts growing attention as a potential therapeutic substance since increasing numbers of scientific publications link H_2_S to many physiological and pathophysiological processes such as hypertension, arteriosclerosis, ischemia-reperfusion injury, preeclampsia and chronic inflammatory diseases (for review see ref. 2). Although H_2_S can be endogenously synthesised by the enzymes cystathionine gamma lyase (CSE), cystathionine beta synthase (CBS) and 3-mercaptopyruvate sulfurtransferase (MPST) the effects of H_2_S have been mainly investigated using exogenously applied H_2_S donors^3^,^4^. In most studies H_2_S releasing sulfur salts (NaSH, Na_2_S, Na_2_S_3,_ Na_2_S_4_) or slow releasing H_2_S substances such as GYY4137^5^ were used to investigate the effects of H_2_S *in vitro* and *in vivo*. Despite a large and growing number of reports describing the effects of H_2_S on cells and tissues, the exact molecular targets affected by H_2_S and its releasing substances are largely unknown. It is believed that H_2_S mainly reacts with cysteine residues (thiols) leading to the formation of persulfides (R-S-SH), a process also referred to as sulfhydration, thereby influencing the localisation, stability and activity of a target protein. Therefore, this specific cysteine modification is regarded as the main switch mediating the cellular response of H_2_S. However, due to its oxidation state H_2_S cannot react with thiols and recent reports indicate that rather polysulfides and thiosulfates are responsible for persulfide formation^6^,^7^,^8^. Indeed, polysulfides can be found as trace amounts in all H_2_S releasing agents and are able to modify cysteine residues in for example, phosphatase and tensin homolog (PTEN) thereby changing its activity^9^.

Due to the chemical properties of persulfides it is challenging to identify proteins containing this type of cysteine modification. With p*K*_A_ values lower than thiols, persulfides show a similar or even better reactivity to electrophilic substances. Furthermore, persulfides also display the properties of thiols forming a disulfide bond. Hence, specific enrichment and subsequent isolation of persulfides on total proteins is challenging since intact proteins usually contain more than one unaffected or modified thiol. Several previous studies demonstrate the detection of persulfides in glyceraldehyde 3-phosphate dehydrogenase (GAPDH), Parkin, Kelch-like ECH-associated protein 1 (Keap1), dual specificity mitogen-activated protein kinase 1 (MEK1), NF-κB, tyrosine-protein phosphatase non-receptor type 1 (PTP1B) and several ion channels using different experimental settings^10^,^11^,^12^,^13^,^14^,^15^,^16^,^17^. However, methods for a proteome-wide identification of persulfides are required to broaden the spectrum of molecular targets for the pharmacological use of H_2_S releasing agents. For the first time, Mustafa *et al*. reported a modified Biotin-switch assay based on the electrophilic reagent MMTS (methyl methanethiosulfonate), which blocks thiols whereas persulfides seemed not to be affected by MMTS^10^,^14^. Another method, which was developed based on the selective replacement of electrophilic substances on persulfides by CN-biotin forming stable thioether conjugates^18^,^19^. Two other approaches based on the fact that persulfide containing proteins and peptides can be selectively eluted with reductants after enrichment with electrophilic biotin conjugates, since persulfides contain an “internal disulfide” bond^20^,^21^. All the methods brought valuable progress in the research on H_2_S signalling.

We describe here a liquid chromatography mass spectrometry-based method that also relies on the selective elution of persulfides by reducing agents with several advantages. First, we used TCA (trichloroacetic acid) to block thiol exchange reactions, which might influence the persulfide pattern in the proteome. Secondly, we implemented a SILAC (stable isotope labelling by amino acids in cell culture) approach that allows simultaneously both the site-specific identification and the quantification of cysteine persulfides upon stimulation with H_2_S releasing agents. Furthermore, metabolic labelling minimises artefacts that occur by sample handling. We describe here the effects of different H_2_S releasing agents on the “persulfidome” in the human embryonic kidney cell line HEK293 with our method called qPerS-SID (quantitative PerSulfide Site IDentification). Using this approach, we demonstrate that persulfides induced by H_2_S releasing agents indeed affect enzymatic activity as exemplified for pyruvate kinase M2 (PKM2).

## Results

### Development of a method for the quantitative identification of cysteine persulfides (qPerS-SID)

**Figure 1:**
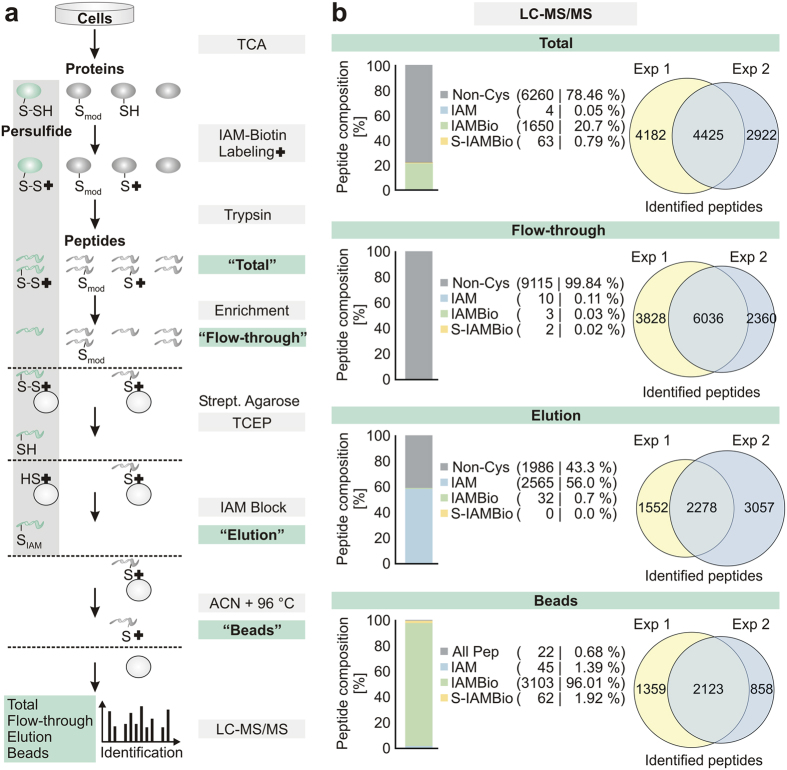
Workflow for the identification of persulfide containing peptides using mass spectrometry. (**a**) Cells were subjected to trichloroacetic acid (TCA) precipitation and subsequently, the thiols and persulfides were labelled using iodoacetyl-PEG2-Biotin (IAMBio). After digestion of the proteins (Total), single peptides containing either labelled persulfides or cysteines were enriched and separated from non-cysteine peptides (flow-through) using streptavidin agarose beads. After several washing steps, persulfide containing peptides were eluted using tris(2-carboxyethyl)phosphine (TCEP) where thiol containing peptides were not affected. The subsequent accessible cysteines were labelled with iodoacetamide (IAM) (Elution). As control, labelled thiol peptides remaining on the beads were eluted using 10 mM TCEP and 80% acetonitrile (ACN) (Beads). Samples of the total, flow-through, elution and bead fraction were subjected to liquid chromatography and mass spectrometry (LC-MS/MS) and the peptides were identified using the PEAKS 7.0 proteomics software. (**b**) Number of identified peptides in the different fractions as described in **a**). Shown are the mean values of two independent experiments. S-IAMBio: Persulfide peptide modified by IAMBio.

The general problem with the identification of post-translational modifications on a proteome scale, using the so-called bottom-up proteomics approach, is the high degree of complexity of a sample and the sub-stoichiometric abundance of any given modification. This makes it rather unlikely to detect modified peptides of interest with mass spectrometry and later on to identify the species in a database search. Therefore, proteome research mainly deals with the specific enrichment of the desired modifications to improve detection sensitivity. This is also true for the detection of persulfides. After tryptic digestion, only about 20% of the peptides would contain cysteines^22^ and we assumed that only a very small subset of these peptides would contain persulfides making detection of persulfides difficult in a direct approach. Therefore, we developed a mass spectrometry-based workflow for the specific enrichment and isolation of persulfides from cultured cells (Fig. 1a): In the first step, we treated HEK293 cells with trichloroacetic acid (TCA), which on the one hand causes a complete lysis of the cells and denaturation of proteins. On the other hand, the strong acidic shift in pH leads to the protonation of the reactive thiolate anion, thus instantly stopping redox reactions^23^ and therefore stabilizing cysteine modifications such as persulfides. Subsequently, we treated the samples with the thiol reactive reagent iodoacetyl-PEG2-Biotin (IAMBio, Fig. 1a). In contrast to the classical biotin switch assay, which first blocks free cysteines followed by a reduction step and subsequent labelling and enrichment of modified cysteines of interest, we performed the labelling reaction first and did not reduce the oxidized cysteine species. This led to the parallel labelling of thiols and persulfides^24^ (Fig. 1a). However, on the protein level it is nearly impossible to distinguish IAMBio labelled persulfides from thiols. To be able to differentiate persulfides from thiols later in a mass spectrometry step, we digested the labelled proteins with trypsin, thus increasing the likelihood of isolating a peptide carrying either a single cysteine or a persulfide. As a control, the input sample was directly subjected to LC-MS/MS analysis and, subsequently, identification of the peptides was performed using the proteomics software PEAKS 7 (Waterloo, Canada, http://www.bioinfor.com^25^). As expected, only 20% of the peptides contained cysteines and the majority of them possessed thiols that were modified by IAMBio (Fig. 1b, Supplementary Table 1). In addition, a very small fraction (0.79%) of the peptides also contained the labelling reagent IAMBio with an additional sulfur atom, which can be considered direct proof of persulfide presence, showing that this cysteine modification is also present in cells not exposed to H_2_S releasing agents (Fig. 1b, Fig. S7, Supplementary Table 1). Next, we enriched and separated the cysteine and persulfide containing peptides from other peptides using streptavidin agarose beads (Fig. 1a). The peptide composition in the non-bound fraction contained hardly any cysteine or persulfide containing peptide (Fig. 1b, Supplementary Table 1) indicating that the thiol and persulfide containing peptides are strongly bound to the streptavidin agarose beads. After this enrichment step, persulfide containing peptides had to be separated from cysteine peptides. This can be achieved by taking advantage of the slight chemical difference between persulfides and thiols. Since persulfides contain an additional sulfur atom, they are labelled and linked to streptavidin beads via a disulfide bond, whereas thiols are bound as a thioether. This allows the selective elution of persulfides from the beads by opening the disulfide bond using a reducing agent such as tris(2-carboxyethyl)phosphine (TCEP) (Fig. 1a). Subsequently, the eluted peptides were treated with iodoacetamide (IAM, Fig. 1a) to improve their detection by LC-MS/MS analysis. In this fraction we identified ~56% (2,565 peptides) of IAM labelled peptides and hardly any IAMBio labelled peptides, which underpins the selective elution and enrichment of putative persulfides (Fig. 1b, Supplementary Table 1). The remaining ~44% of the eluted peptides bound non-specifically to the streptavidin beads and did not contain any cysteines. Thus, these peptides were not considered for further analysis. An additional washing step and transfer of beads to a fresh tube could further reduce non-specific bound peptides. As a further control, we analysed bead-bound peptides by boiling the samples in the presence of acetonitrile to remove all remaining peptides from the beads (Fig. 1a). As expected, the majority (~96%) of the identified peptides possessed solely the IAMBio label (Fig. 1b, Supplementary Table 1) demonstrating that the thioether-bound thiol peptides were unaffected by the previous TCEP treatment.

Although we identified 2,565 putative persulfide containing peptides with this elution strategy, there are two conditions, which can lead to the identification of false positive peptides. Firstly, two peptides might be interconnected via an “interpeptide” disulfide bond and one of these peptides must contain at least one additional cysteine labelled by IAMBio (Fig. S1a). This would lead to a similar elution behaviour as described here for persulfides. As an indirect marker of these false positives the amount of peptides found at the beads carrying both an IAM and IAMBio labelling (Fig. S1a) can be assessed. In our experiments, only 1.32% of the identified IAMBio peptides additionally contained the IAM label, leading to the assumption that the likelihood of these false positives is very low (Fig. S1b, Supplementary Table 1), especially if one considers that some of the double modified peptides might also originate from mixed persulfides/thiols or other thiol oxidations than disulfides. In summary, the number of such false positive peptides is negligible in our experimental setting. Secondly, electrophilic reagents like NEM, MMTS, or IAM also show a slow reactivity towards primary amines^26^ and sulfenic acid *in vitro*^27^. To circumvent this problem, we applied the SILAC strategy described in the next section, which allowed the identification and quantification of differently treated samples and thus, resulted in a higher specificity. This method is further referred to as qPerS-SID (quantitative PerSulfide Site IDentification).

### A SILAC approach reveals that H_2_S releasing donors differ in their efficacy to induce persulfides

**Figure 2:**
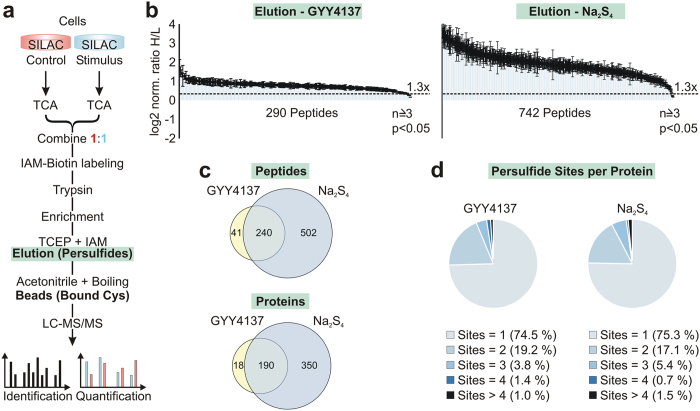
Workflow for the detection of quantitative changes of persulfide formation upon treatment with different H_2_S donors. (**a**) The experiment was performed as described in Fig. 1a, except that the cells were grown in heavy or light SILAC medium (red/blue) allowing the quantification of induced persulfides. In order to induce persulfide formation the cells were treated 30 min with 200 μM of the polysulfide Na_2_S_4_ and 1 mM of the slow releasing H_2_S reagent GYY4137 for 4 h. Peptides were quantified by MaxQuant. (**b**) Elution profile of GYY4137 and Na_2_S_4_ stimulated cells. The experiment was performed four times (two times forward, two times reverse) and the mean values and error bars of significant peptides (p ≤ 0.05) appearing in at least 3 of 4 experiments are plotted as log2 value. The median of the heavy to light ratio (H/L) of non-cysteine peptides in the total fraction was used for normalisation. Dashed line: threshold of peptides that were at least 30% induced (1.3×). (**c**) Venn diagram of peptides and proteins influenced by GYY4137 and Na_2_S_4_. (**d**) Diagram showing the number of persulfide sites per protein changed by treatment with GYY4137 and Na_2_S_4_.

To this end, we used lysates from HEK293 cells grown in SILAC medium that were treated with different H_2_S donors for the induction of persulfide formation (Fig. 2a). In most studies regarding the molecular action of H_2_S the sulfur salts NaSH and Na_2_S, the slow releasing H_2_S donor GYY4137 and polysulfides such as Na_2_S_4_ are used to analyse H_2_S-driven signalling pathways. Therefore, we chose these compounds for the stimulation of HEK293 cells. Cell lysates were then subjected to our mass spectrometry workflow. For each donor, the experiment was performed in quadruplicates (two times forward labelling, two times reverse labelling) and data obtained were quantified using MaxQuant^28^. Only peptides identified in at least 3 out of 4 samples were taken for quantitative evaluation. We quantified 368 peptides when using GYY4137 (Supplementary Table 2), 774 for Na_2_S_4_ (Supplementary Table 3), 843 for Na_2_S (Fig. S2, Supplementary Table 4) and 725 for NaSH (Fig. S2, Supplementary Table 5) as a stimulus. Exposure of HEK293 cells to GYY4137 and Na_2_S_4_ led to a clear and quantifiable induction of persulfides (Fig. 2b). As depicted in Fig. 2b, the polysulfide Na_2_S_4_ was much more efficient inducing persulfide formation compared to the slow releasing donor GYY4137. In contrast, we could not observe quantitative differences in lysates from NaSH- or Na_2_S-treated cells compared to controls (Fig. S2), indicating that the H_2_S-releasing compounds tested, differ drastically in their efficacy to induce persulfide formation. We also checked the rate of possible false positive peptides for all donors, but this rate was again very low and ranged between 1.33% and 1.68% (Fig. S1, Supplementary Table 6) confirming the reliability of our method.

Since we found a quantitative enhancement of persulfide formation by GYY4137 and Na_2_S_4_, we used the data of these H_2_S donors for further analysis. To be certain that we examined peptides that were authentically responding to those stimuli, we set a threshold considering only peptides that showed an enhanced enrichment of 30% or more compared to controls (Fig. 2b). To further enhance confidence and reproducibility we considered only peptides for further evaluation that met significance in a one-sample *t*-Test (Fig. S3). Choosing these criteria, we first compared the number of peptides, which were evenly responding to both donors. In this setting, we found in total 783 peptides that formed persulfides of which 240 were responding to both donors (Fig. 2c). Since for both GYY4137 and Na_2_S_4_ the total protein number (208 and 540, respectively) was overlapping nearly three quarters with the peptide numbers (281 and 742, respectively) we compared the number of persulfide sites per protein (Fig. 2d). For both donors in more than 74% of the proteins there was only one persulfide site found, explaining the low deviation of peptide number versus protein number. These data also indicate that there is a specific cysteine residue forming a persulfide, rather than an unspecific modification caused by the donors.

### Bioinformatic analysis of persulfide containing peptides and proteins affected by GYY4137 and Na_2_S_4_

**Figure 3:**
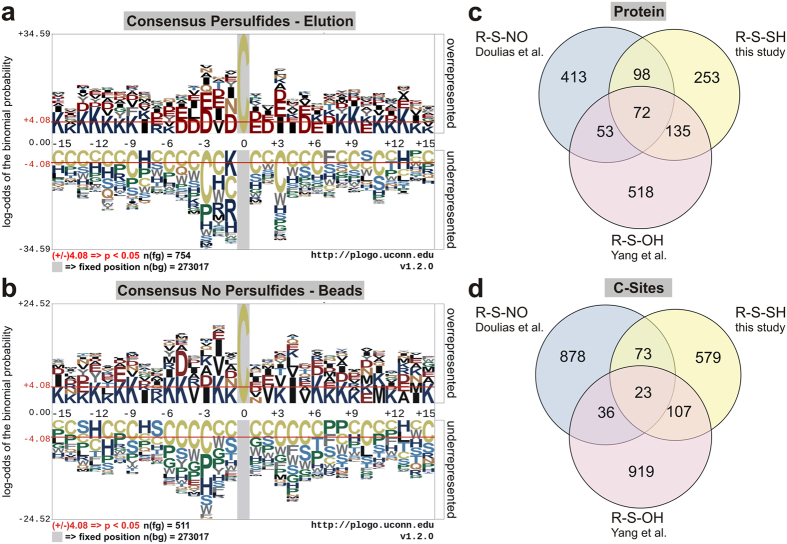
Amino acid enrichment analysis of persulfide containing peptides and proteomic comparison of persulfides, S-nitrosated cysteines and S-sulfenylated cysteines. (**a**) pLogo^29^ analysis of enriched amino acids up to 15 positions before or after the reactive cysteine. Red: negative charged amino acids, blue: positive charged amino acids, black: hydrophobic amino acids (**b**) pLogo analysis of enriched amino acids 15 positions before or after cysteines that were not affected by H_2_S stimulation found in the beads fraction. (**c**) Venn diagram comparing proteins that were S-sulfhydrated (R-S-SH, persulfides) by GYY4137 and Na_2_S_4_ as well as S-sulfenylated (R-S-OH) and S-nitrosated (R-S-NO). (**d**) Venn diagram comparing S-sulfhydrated (R-S-SH, persulfides), S-sulfenylated (R-S-OH) and S-nitrosated peptides (R-S-NO).

Based on these findings, it is tempting to speculate that the specific persulfide sites contain a consensus amino acid composition. Therefore, we applied the pLOGO algorithm^29^ to calculate the likelihood of repeatly appearing amino acids up to 15 positions surrounding the identified persulfide site (Fig. 3a, Fig. S4a). Interestingly, it seemed that the negatively charged amino acids glutamate and aspartate appeared more frequently in close proximity to the reactive cysteine, especially at position -3. As a control, we checked cysteine peptides bound to the streptavidin beads carrying an IAMBio labelling and thus were not corresponding to persulfides (Fig. 3b, Fig. S4b). The amino acids of those peptides appeared to be arranged randomly. However, there was a trend towards positive charged amino acids within the peptide sequence. This may be due to the use of trypsin for the generation of peptides, which cuts after arginine and lysine. This data clearly indicates that the H_2_S sensitive cysteines, rather than the non-reactive cysteines, are present in vicinity of a particular amino acid composition. Yang *et al*.^30^ reported a similar pattern of negatively charged amino acids surrounding S-sulfenylated cysteines at position +3 and +4. Moreover, they also analysed the amino acid composition surrounding S-nitrosated cysteines identified by Doulias *et al*.^31^ and showed that hydrophobic amino acids appear more frequently in their proximity. We also detected hydrophobic amino acids surrounding the persulfide forming cysteine although with less frequency. Several publications strongly indicate a cross-talk of NO, ROS and H_2_S all known to potently trigger redox-based thiol switches (for review see ref. 32). The similarities between the amino acid composition of S-sulfenylated cysteines, S-nitrosated cysteines and persulfides prompted us to compare our findings with the results from Yang and Doulias^30^,^31^. We found an overlap of 72 proteins affected by all three redox modifications (Fig. 3c, Supplementary Table 7). Among them, we identified triosephosphate isomerase (TPI), peroxiredoxin 6 (PRDX6), GAPDH and superoxide dismutase (SOD1), proteins that are known to be redox-regulated. In a previous study, it was shown that a persulfide formed at cysteine C111 of SOD1 stabilizes the enzyme against oxidation-induced aggregation without affecting its activity^33^. Therefore, it is tempting to speculate that H_2_S-induced persulfide formation on SOD1 may play a role in familial amyotrophic lateral sclerosis by influencing the aggregation of SOD1^34^. For GAPDH three cysteines C152, C156 and C247 have been identified that form persulfides that affect its activity^10^,^35^. For both, SOD1 and GAPDH, these specific cysteine sites for persulfide formation have been confirmed by our mass spectrometry-based approach, which demonstrates that this technique is suitable for site-specific analysis of persulfides. In total, 98 proteins were found to be modified by both NO and H_2_S (Fig. 3c, Supplementary Table 7) including thioredoxin (TXN). It is known that TXN contains a nitrosocysteine at position 73, which we found also underwent H_2_S-triggered persulfide formation indicating a crosstalk between NO and H_2_S. 135 proteins found in our study showed an overlap with S-sulfenylated proteins (Fig. 3c, Supplementary Table 7). Among them, we found glutathione-S-transferase P (GSTP1), a protein involved in glutathionylation of substrates to be modified at C48. Interestingly, this site differs from the S-sulfenylation site C102, showing that different redox active molecules might also target different thiols on one protein. That NO, H_2_O_2_ and H_2_S target different cysteine residues at the same protein is also underlined by the observation that the overall overlap of the specific site of thiol modification is lower than the overlap of proteins (Fig. 3d). Nevertheless, our data further hints for the immense crosstalk between H_2_S and other redox active molecules like ROS and NO on the molecular level^32^.

**Figure 4:**
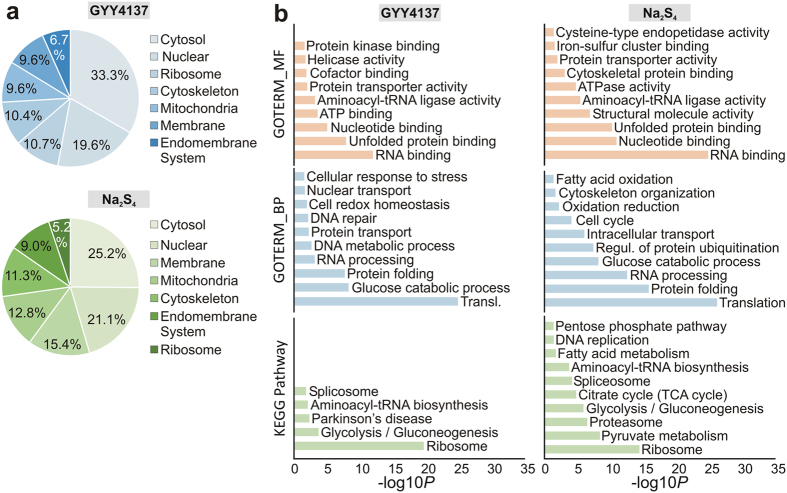
Protein enrichment analysis of persulfides affected by GYY4137 and Na_2_S_4_. (**a**) Cellular compartment enrichment analysis using the DAVID program^59^. (**b**) GO term enrichment analysis of biological processes (GOTERM_BP), molecular function (GOTERM_MF) and KEGG pathway enrichment analysis.

Next, we analysed the function and localisation of the H_2_S targeted proteins found in our study *in silico*. To this end, we used the DAVID program (database for annotation, visualization and integrated discovery) to run gene onthology term (GO term) enrichment analyses. Proteins forming persulfides in the presence of GYY4137 and Na_2_S_4_ were found in all cellular compartments (Fig. 4a). Nearly half of them were present in the cytosol and nucleus, supported by the GO-TERM analysis for molecular functions and biological processes such as RNA binding and processing, protein folding, translation and nucleotide binding (Fig. 4b). However, we detected hardly transcription factors regulated by GYY4137 or Na_2_S_4_, which might be due to lower abundance that limits their detection compared to more prominent peptides. Moreover, we found redox active proteins to be enriched in both GYY4137 and Na_2_S_4_ treated samples (e.g. PRDX6, TXN, GAPDH, PRDX4, malate dehydrogenase (MDH)) showing the redox active nature of H_2_S. KEGG pathway analysis suggesting the high impact of H_2_S on metabolic pathways for both GYY4137 and Na_2_S_4_ (Fig. 4b). Additionally, the data for Na_2_S_4_ indicate a role of H_2_S in proteasomal processes and protein ubiquitination. Taken together, these results clearly indicate that H_2_S targets widespread pathways and processes within a cell.

### Functional characterisation of persulfide formation on pyruvate kinase M2

**Figure 5:**
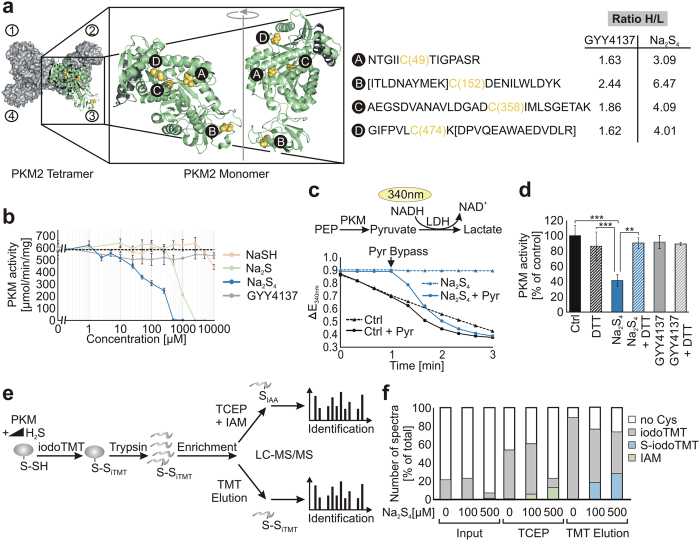
*In vitro* characterisation of persulfide formation on PKM2. (**a**) Structure showing the human PKM2 monomer and tetramer (PDB: 3SRH). Cysteine peptides forming persulfides are highlighted (yellow spheres; A, B, C, D). Black: intersubunit contact site; letters in square brackets: sequence of misscleaved peptide. (**b**) Purified PKM2 from rabbit muscle was incubated with increasing concentrations of Na_2_S_4_, Na_2_S, NaSH and GYY4137. PKM2 activity (μmol/min/mg) was measured in a coupled enzyme assay with LDH as second enzyme monitoring the consumption of NADH at 340 nm. (**c**) The activity of PKM2 was followed as decline in absorption at 340 nm. After 1 min 5 mM pyruvate was added to bypass the reaction catalysed by PKM2. (**d**) The experiment was carried out as described in (**b**) except that 1 mM DTT was added in parallel to treatment with 200 μM Na_2_S_4_. Data are means +/− SD, **p < 0.01 Na_2_S_4_ vs Na_2_S_4_ + DTT, ***p < 0.001 Ctrl as well as DTT vs Na_2_S_4_. (**e**) Workflow to confirm that persulfides are formed at PKM2. PKM2 was incubated with 100 μM and 500 μM Na_2_S_4_ or kept untreated. Induced persulfides were modified with iodoTMT similar to qPerS-SID. After digestion with trypsin the persulfide peptides were enriched using an anti-iodoTMT resin and subjected to TCEP elution followed by IAM blocking as described for the proteomic approach. In parallel, direct labelled persulfides (S-iodoTMT) were eluted using iodoTMT elution buffer. The eluted peptides were subjected to LC-MS/MS measurement and the peptides were identified using PEAKS 7.0. (**f**) Spectra counts of iodoTMT labelled cysteine peptides (iodoTMT), persulfide peptides identified according to the qPerS-SID protocol (TCEP elution, IAM) and iodoTMT labelled persulfide peptides (TMT elution, S-iodoTMT).

In order to investigate the functional consequences of persulfide formation more closely we chose pyruvate kinase M2 (PKM2), which we found to possess persulfides on cysteines C49, C152, C358 and C474 induced by Na_2_S_4_ and GYY4137 (Fig. 5a, Fig. S6). PKM2 catalyses the rate-limiting step of glycolysis by generating pyruvate from phosphoenolpyruvate, thereby producing ATP. It is a key player in controlling the metabolic state of a cell and thus, linked to cancer development^36^. Previous studies had already implied a redox regulation of PKM2 by ROS^37^. To further address the impact of persulfide formation on PKM2 activity, we used purified PKM2 from rabbit muscle and performed a coupled enzyme assay, which uses LDH as the terminal reaction enzyme^38^. Upon pre-incubation of PKM2 with the H_2_S donors Na_2_S_4_, Na_2_S and NaSH we observed an inhibition of enzyme activity in a concentration dependent manner (Fig. 5b). The polysulfide Na_2_S_4_ had the highest potency for inhibiting PKM2 followed by Na_2_S. Pre-treatment of PKM2 with NaSH had only minor effects on PKM2 inhibition. In contrast to our proteomic data, pre-treatment with the slow releasing donor GYY4137 had no effect on PKM2 activity, indicating that a cellular compound present in intact cells or a pre-oxidation step by another molecule is required to catalyse the persulfide formation. To test that the observed effect on PKM2 activity is really due to its specific inhibition and not based on an inhibition of LDH we bypassed the reaction by adding pyruvate. The inhibitory effect of Na_2_S_4_ could be reversed when pyruvate was added showing that LDH activity was not affected under the conditions chosen (Fig. 5c). Like in disulfides the chemical bond between the two sulfur atoms in persulfides is susceptible to the treatment with reducing agents. This led us to test whether the inhibitory effect of Na_2_S_4_ can be antagonized by co-treatment with the reductant dithiothreitol (DTT) (Fig. 5d). Indeed, in the presence of DTT, the activity of PKM2 was comparable to the mock-treated control sample, reinforcing the conclusion that the inhibition of PKM2 is generated by a redox modification of cysteine residues. To prove that the cysteine modification is indeed a persulfide and to confirm our proteomics data we directly assessed the persulfide formation using mass spectrometry. For this, we applied our established workflow on purified PKM2 with a minor modification in order to directly analyse persulfides through the identification of labelled cysteines that contain a second sulfur atom (Fig. 5e). To this end, we used iodoTMT instead of IAMBio as a labelling reagent, because iodoTMT is known to improve the ionization and fragmentation of these peptides^39^. We incubated purified PKM2 with increasing amounts of Na_2_S_4_ and subsequently labelled it with iodoTMT (Fig. 5e). With increasing amounts of Na_2_S_4_ we observed an increase in elution of IAM labelled cysteine peptides indicating that persulfides are formed upon Na_2_S_4_ treatment (Fig. 5f). The same holds true when looking at persulfides modified directly by iodoTMT appearing as iodoTMT with an additional sulfur atom (Fig. 5f). Moreover, when comparing the peptide spectra, we identified exactly the same peptides that form persulfides with our qPerS-SID protocol as with the direct labelling (Fig. S6). This clearly demonstrated that this indirect approach using reducing agents in order to elute persulfide peptides is suitable for their identification. We also performed the experiment with 500 μM and 2.5 mM GYY4137, but detected hardly any persulfide formation in either mass spectrometry-based approaches (data not shown) supporting the finding mentioned above that in a cell free environment GYY4137 does not react with PKM2.

## Discussion

Here, we describe a mass spectrometry-based method for the enrichment and site specific identification of cysteine persulfides in mammalian cells. One major problem of identifying persulfides on whole proteins is the ambivalent behaviour of persulfides. On the one hand, they show a similar reactivity towards electrophiles to that shown by thiols. On the other hand, due to their intrinsic disulfide bond, it is very challenging to distinguish persulfides from other cysteine modifications when using a commonly used biotin switch technique^40^,^41^. Since most proteins contain more than one cysteine, it is nearly impossible, to enrich and detect persulfides on intact proteins. However, we could clearly demonstrate that on the peptide level the ambivalent properties of persulfides allow their selective elution by reducing agents and their subsequent identification.

In order to detect quantifiable differences between different stimuli and further reduce the detection of potential false positives such as labelled primary amines and sulfenic acids, we extended our approach with a SILAC labelling technique (qPerS-SID). To achieve this high specificity, we chose only peptides for further consideration that were identified at least in 3 out of 4 experiments. Furthermore, the SILAC approach allowed the quantification of persulfides and therefore, the only peptides that were considered as relevant persulfides, were those that responded significantly with a 30% or higher rate to stimulation by the H_2_S donors, compared to untreated controls. For the stimulation of HEK293 cells the sulfur salts NaSH and Na_2_S, the polysulfide Na_2_S_4_ and the slow H_2_S releasing agent GYY4137 were used. We detected quantitative differences with Na_2_S_4_ and GYY4137 stimulation and only minor differences in case of Na_2_S and NaSH treatment indicating that the diverse H_2_S releasing substances differ in their potency to induce persulfides. For NaSH and Na_2_S, the selected point in time might have been unfavourable or the concentration was not optimal to induce measureable differences. Indeed, some reports described that an administration of NaSH and Na_2_S not as single dose but instead repetitively over several days was required to evoke biological consequences^42^,^43^. Furthermore, it might be that the biological effects caused by NaSH and Na_2_S are not primarily dependent on persulfide formation, but instead on their anti-oxidative properties or on a possible reaction with metal centres of proteins.

In total, we detected 281 tryptic peptides responding to GYY4137 and 742 peptides responding to Na_2_S_4_ stimulation that were considered as persulfide containing peptides. Since tryptic digestion may generate peptides that are too small or too big for detection, the use of additional proteases such as chymotrypsin or elastase, which cut at different amino acids than trypsin might further increase the number of identified persulfide containing peptides.

We also identified peptides that contained two or more cysteine residues. Since redox active proteins often contain cysteine motifs like CXXC or CXC, e.g. in zinc finger proteins, we also analysed such patterns in our setting. However, we could not observe a significant enrichment of these peptides after treatment with GYY4137 or Na_2_S_4_ indicating that such motifs are obviously not a preferential target for persulfide formation.

Interestingly, the number of proteins affected by H_2_S donors (GYY4137: 208; Na_2_S_4_: 540) hardly differed compared to the amount of peptides (GYY4137: 281; Na_2_S_4_: 742) showing that the reaction is directed to specific cysteine residues, rather than appearing randomly at cysteines. This is underlined by our observation that cysteines that are prone to persulfide formation are often surrounded by negatively charged amino acids. We believe that negatively charged amino acids may deprotonate a neighbouring cysteine thus promoting the formation of a much more reactive thiolate anion.

There is crosstalk between redox active molecules such as H_2_S, NO and H_2_O_2_ which is corroborated by comparing our data with earlier proteomic studies investigating S-sulfenylated^30^ and S-nitrosated^31^ proteins. In a coordinated manner, different cysteine modifications as induced by ROS, NO or H_2_S can influence a protein differently regarding activity, localisation and stability. For example, it has been shown for the NF-kB subunit p65, that S-nitrosation of C38^44^,^45^ inhibits its activity, whereas persulfide formation leads to its activation^14^. For GAPDH, it has been reported that S-nitrosation at C150 also inhibits the catalytic activity. The same cysteine can also be targeted by H_2_S, which has been confirmed by our study. However, whether this positively or negatively affects GAPDH activity is under debate since conflicting results have been reported^10^,^35^. Furthermore, we found a H_2_S-induced persulfide on C73 of TXN. This cysteine residue drives the nitrosation process of target proteins such as caspase 3 (CASP3)^46^,^47^. Thus, one can speculate that H_2_S might also influence the nitrosation of CASP3 and therefore, play a role in apoptosis. GO term and KEGG pathway analyses showed that H_2_S releasing substances can target all subcellular compartments thereby influencing a wide variety of biological processes. Interestingly, we found that H_2_S targets nearly all aspects of energy metabolism (e.g. glycolysis, TCA cycle) and thus might strongly influence the metabolic state of a cell, which has been confirmed by a recent study^20^. This might also explain the beneficial effects observed in ischemic preconditioning^48^,^49^,^50^ and the controversial effects gained by H_2_S in cancer treatment and development^42^,^51^,^52^, which might be deduced by a possible hypoxia-like condition upon H_2_S treatment. KEGG pathway analysis also showed a link of persulfide formation and Parkinson’s disease. Recently, persulfide formation of the ubiquitin protein ligase parkin has been reported^11^. Although we did not identify this particular protein in our experiments, we were able to detect persulfide formation of other proteins potentially involved in Parkinson’s disease such as park7, ubiquitin-like modifier-activating enzyme 1 (UBA1) and voltage-dependent anion-selective channel protein 1 (VDAC) suggesting a possible impact of H_2_S on this neurodegenerative disease. Indeed, some *in vitro* and *in vivo* studies corroborate that H_2_S plays a role in Parkinson’s disease^11^,^53^,^54^. Future experiments are necessary to shed light on the impact of persulfide formation in the development and progression of this disease.

Recently, a tag switch method was developed for the identification of persulfide containing proteins^18^,^19^ by forming stable thioether conjugates using CN-biotin. The authors combined this method with 2D gel electrophoresis to analyse persulfides in CSE overexpressing lung carcinoma A549 cells and found approximately 24 different proteins to form persulfides among them protein disulfide isomerase, heat shock proteins, aldo-keto reductase, GAPDH, enolase, and phosphoglycerate kinase. Remarkably, we also identified among many others, the same proteins with our approach, clearly demonstrating the high reproducibility of the results obtained by both methods. A comparison of the results of this study with our results is shown in Fig. S5 and Supplementary Table 8. Our method further allows the direct identification of a specific persulfide site within a protein and the quantitative evaluation of persulfides in cultured cells treated with H_2_S donors compared to unstimulated controls. Furthermore, in contrast to the reported studies^10^,^19^,^21^ our method relies on a simple preparation of samples with direct connection to mass spectrometry and we think that this increases the identification of sulfhydrated proteins with lower abundance. More recently Gao *et al*.^20^ described a similar strategy to ours in order to analyse persulfide formation in metabolic reprogramming. They used native conditions and low amounts of maleimide-biotin assuming that persulfides show a higher reactivity and accessibility compared to thiols. Our strategy uses immediate denaturation at low pH e.g. by TCA precipitation to prevent further thiol exchange reactions^23^ and thus, “freeze” the persulfide and redox-state within the cellular proteome. Moreover, our denaturing set up allows the detection of persulfides in proteins that are not soluble under mild lysis conditions to cover the complete cellular proteome. This might explain the slightly higher number of sulfhydrated proteins we achieved with our method (Fig. S5 and Supplementary Table 8). In addition, we used a metabolic labelling strategy with SILAC, which further minimizes artefacts that occur by sample handling that may cause differences in quantification. Nevertheless, when comparing the number of identified persulfide containing proteins by Gao *et al*., the tag-switch method applied by Ida *et al*. and our study, one can clearly see that the selective elution of persulfides by reducing agents allows detection of more sulfhydrated proteins than the tag switch strategy (Fig. S5 and Supplementary Table 8).

In our last set of experiments, we focused on PKM2, a key regulator of glycolysis influencing the metabolic state of a cell. Using purified rabbit PKM2, we could demonstrate that the four cysteines C49, C152, C358 and C474 found in the proteomic approach, clearly form persulfides upon treatment with Na_2_S_4_ leading to enzymatic inhibition *in vitro*. Na_2_S_4_ treatment had the highest inhibitory effect followed by Na_2_S and NaSH underlining the possibility that trace amounts of polysulfides found in NaSH and Na_2_S are responsible for the effect rather than H_2_S per se^9^. This may also explain why treatment of short duration with NaSH and Na_2_S showed hardly any detectable effect on persulfide formation in the proteome of HEK293 cells. Interestingly, GYY4137 did not inhibit PKM2 under cell-free conditions although we observed persulfide formation with treatment in intact cells. It is probable that the compound GYY4137 follows a different reaction to the sulfur salts, and that in this case persulfide formation relies on a pre-oxidation of the protein or other cellular compounds. In line with this, a publication reports that persulfide formation relies on sulfenic acid *in vitro*^55^. Another possibility might be that other low molecular weight substances like GSH or cysteine might be involved in transmitting the H_2_S signals intracellularly. It has been demonstrated that such molecules form S-polythiolation^19^ and GYY4137 might therefore rely on these intracellular compounds to be able to modify cysteines. Moreover, GYY4137 might activate or serve as a substrate for an enzyme, which might introduce persulfides on substrate proteins e.g. sulfurtransferases^6^,^56^.

In summary, we developed qPerS-SID a robust mass spectrometry-based method allowing the site specific identification and quantification of persulfides in cultured cells. Our results provide a solid basis to initiate further investigations regarding the biological effect of H_2_S and its releasing substances. The detailed knowledge of the “persulfidome” may facilitate the development of H_2_S-based therapies to treat a variety of disorders such as cardiovascular disease and cancer.

## Methods

### Cell culture

HEK293 cells were maintained in a 5% CO_2_ humidity atmosphere at 37 °C and grown to 80–90% confluency in DMEM medium containing 10% FCS and Penicillin /Streptomycin. For quantification of persulfides HEK293 cells were grown in medium containing heavy (^13^C_6_,^15^N_2_-L-Lysine, ^13^C_6_,^15^N_4_-L-Arginine) and light amino acids supplemented with 200 mg/L L-Proline for SILAC (*Silantes*). For investigating H_2_S releasing substances cells were treated 4 h with 1 mM GYY4137 (*Cayman chemical*) and 30 min with 200 μM Na_2_S_4_ (*Alfa Aesar*), 200 μM NaSH or 200 μM Na_2_S (*Sigma*) in medium without FCS. The experiment was carried out in quadruplicates for each H_2_S releasing substance.

### Sample preparation for qPerS-SID

After stimulation with the different H_2_S donors, cells were treated with 12% TCA in order to denature and precipitate proteins and avoid further thiol exchange reactions. Samples were sonified 7× (amplitude 30%) using the sonifier device 450 (*Branson*). After TCA precipitation overnight at −20 °C, the protein pellet was resuspended in an IAMBio-lysis buffer (6 M urea, 200 mM Tris pH 8.2, 100 mM NaCl, 2% SDS, 20 mM iodoacetyl-PEG2-Biotin (*Thermo scientific*), 5 mM EDTA) and incubated 2 h at 37 °C in the dark allowing the parallel labelling of thiols and persulfides. The pH was checked for optimal labelling conditions. In the case of SILAC experiments equal amounts of heavy and light samples were mixed with 3 volumes of UA-buffer (8 M Urea in 100 mM Tris, pH 8.5). The samples were subjected to filter aided sample preparation (FASP) as described previously^57^ using 30 kDa cut-off filter (*MERCK Millipore)*. Proteins were digested overnight at 37 °C using a digestion buffer (1 μg trypsin/200 μg protein, 0.01% Protease Max (*Promega*), 50 mM ammonia bicarbonate (ABC)). Peptides were eluted by centrifugation and an aliquot of 35 μg was taken as a total control. The remaining peptides were subjected to biotin enrichment using high affinity streptavidin agarose beads (*Thermo scientific*) for 4 h at 4 °C. 35μg were taken as the non-bound control. The streptavidin beads were washed 3 times in 100 mM Tris pH 7.5, 45 mM ABC and once with LC-MS grade water. Subsequently, persulfides were specifically eluted by incubating the beads for 30 min at 30 °C with 5 mM TCEP in 10 mM Tris pH 8.0. Afterwards, the resulting free thiols were blocked with 30 mM iodoacetamide. As a control, peptides bound to the beads were eluted by boiling in 0.5% acetic acid, 80% acetonitrile, 10 mM TCEP. Peptides were dried and reconstituted in 0.5% acetic acid. Desalting of peptide fractions was performed using C18 stage tips as described^58^. Subsequently, the peptides were subjected to LC-MS analysis.

### Liquid chromatography / mass spectrometry (LC/MS) and peptide identification

LC-MS was performed on a Q Exactive Plus (*Thermo Scientific*) equipped with an ultra-high performance liquid chromatography unit (Dionex Ultimate 3000, *Thermo Scientific*) and a Nanospray Flex Ion-Source (*Thermo Scientific*). Peptides were loaded on a C18 reversed-phase precolumn (*Thermo Scientific)* followed by separation on an in-house packed 2.4 μm Reprosil C18 resin (*Dr. Maisch GmbH*) picotip emitter tip (diameter 100 μm, 10 μm tip width, 15 cm long, *New Objectives*) using a gradient from 5% to 36% acetonitrile (v/v) in 0.1% formic acid (v/v) for 60 min with a flow rate of 300 nl/min. MS data were recorded by data dependent acquisition Top10 method selecting the most abundant precursor ions in positive mode for higher-energy collisional dissociation (HCD) fragmentation. The full MS scan range was 300 to 2,000 m/z with a resolution of 70,000 and an automatic gain control (AGC) value of 3*10^6^ total ion counts with a maximal ion injection time of 160 ms. MS spectra were recorded in the profile mode. Only higher charged (2+) precursor ions were selected for MS/MS scans with a resolution of 17,500. The scan range for MS/MS fragments was between m/z 200 and m/z 2,000. The isolation window was 2 m/z and the AGC value was set to 10^5^ ions with a maximal ion injection time of 150 ms. Selected ions were excluded in a time frame of 30 s following the fragmentation event.

For peptide identification Xcalibur raw files were directly analyzed by Peaks 7.0 software for proteomics (Waterloo, Canada, http://www.bioinfor.com25) using the Uniprot human reference proteome (April, 8th, 2015, 68506 entries). The enzyme specificity was set to Trypsin, missing cleavages were limited to 3. Initial monoisotopic precursor mass error tolerance was set to 15 ppm and fragment ion tolerance to 0.02 Da. The maximum number of posttranslational modifications (PTM) per peptide was limited to 3. Carbamidomethylation (+57.02 Da), methionine oxidation (+15.99 Da), deamidation on asparagine or glutamine (+0.98 Da), acetylation (+42.01 Da) and iodoacetyl-PEG2-biotinylated cysteine (+414.19 Da) as well as iodoacetyl-PEG2-biotinylated persulfide (+446.17 Da) were used as variable modification. Only peptides with a false discovery rate of less than 1% were considered for further evaluation.

Quantitative analyses were performed using the MaxQuant software version 1.5.2.8^28^ with the same parameters as for the PEAKS search excluding iodoacetyl-PEG2-biotinylated persulfide (+446.17 Da). Additionally, the SILAC labels (K: +8.01 Da; R: +10.01 Da) were included. Heavy/Light ratios of peptides were normalised to the median of non-cysteine peptides in the control fraction.

### PKM2 enzyme activity assay

Pyruvate kinase activity was measured as described previously^38^. In brief, purified PKM2 from rabbit muscle (*Sigma*) was incubated 30 min at 37 °C in the presence or absence of Na_2_S_4_, Na_2_S, NaSH and GYY4137. Afterwards, pretreated PKM2 was added to lactate dehydrogenase in an enzyme buffer (30 mM ADP, 50 mM phosphoenolpyruvate (PEP), 5 mM NADH) and absorption was recorded for 3 min at 340 nm using spectrophotometer spectra max M5e (*Molecular Devices*).

### LC-MS/MS analysis of purified PKM2

Purified PKM2 from rabbit muscle was subjected to treatment with different concentrations of Na_2_S_4_ (0 μM, 100 μM and 500 μM) and GYY4137 (0 μM, 500 μM and 2500 μM) for 30 min at 37 °C and subjected to labeling with iodoTMT (200 mM Tris pH 8.5, 100 mM KCl and 4.4 mM iodoTMT (*Thermo Scientific*)) for 1 h at 30 °C in the dark allowing the parallel labeling of thiols and persulfides. Subsequently, the protein was washed twice with 50 mM ammonia bicarbonate (ABC) and subjected to sample preparation (FASP) as described above. After centrifugation, one third of peptides were directly used as input control and purified by stage tips. The remaining peptides were incubated for 2 h at 4 °C with iodoTMT antibody beads (*Thermo scientific*). After washing, one half was subjected to TCEP elution as described for qPerS-SID. The rest of the peptides were eluted using the provided iodoTMT elution buffer according to the manufacturers’ protocol followed by stage tip purification. All samples were subjected to LC-MS/MS analysis on a LTQ-Orbitrap XL (*Thermo Scientific*) equipped with a high performance nano liquid chromatography unit (Agilent 1200). Peptides were loaded on a C18 reversed-phase precolumn (Zorbax 300SB-C18, *Agilent Technologies*) and separated within 60 min on a 3 μm Reprosil-Pur C18 resin (*Dr. Maisch GmbH*) in-house packed picotip emitter (diameter 75 μm, 15 cm long, *New Objectives*) using a gradient from 4% to 50% acetonitrile in 0.5% formic acid.

MS full scans were measured with a resolution of 30,000 and a scan range of 300–2,000 Da. Collision-induced dissociation (CID) was performed with a normalized collision energy of 35, an activation Q of 0.25 and activation time of 30 s. CID spectra in the ion trap were scanned with the accuracy provided by the enhanced scan mode. Spectra in the orbitrap detector were recorded at resolution of 15,000. The minimum ion intensity was 2,000 and precursor window was set to 2 Da. In addition, a second run with HCD fragmentation and first fixed mass of 110 m/z was used. Data analysis was performed by PEAKS 7.0 software using the Uniprot rabbit database (October 28^th^, 2015, 21183 entries) with an FDR of 5%. As Enzyme specificity we set Trypsin and 3 missed cleaved, no non-specific cleavages allowed. As variable modifications we allowed carbamidomethylation (+57.02 Da), deamidation (+0.98 Da), oxidation at methionine (+15.98 Da), iodoTMT on persulfides (+356.18 Da) and iodoTMT on cysteines (+324.22 Da). Mass accuracy for MS search was 10 ppm, and 0.8 Da for the MS/MS, but we also repeated the searches with MS/MS mass accuracy 0.02 Da to identify the high resolution spectra exclusively.

### Bioinformatics analysis

Localisation, KEGG pathway and GO term analyses were performed using the functional annotation program DAVID v6.7^59^ with p-values < 0.05. Sequence alignments were performed using pLOGO^29^ v1.2.0 with a p-value < 0.05. The Structure (PDB: 3SRH) of PKM2 was visualised using Pymol v1.1.

## Additional Information

**How to cite this article**: Longen, S. *et al*. Quantitative Persulfide Site Identification (qPerS-SID) Reveals Protein Targets of H_2_S Releasing Donors in Mammalian Cells. *Sci. Rep*. **6**, 29808; doi: 10.1038/srep29808 (2016).

## Supplementary Material

Supplementary Information

Supplementary Table 1

Supplementary Table 2

Supplementary Table 3

Supplementary Table 4

Supplementary Table 5

Supplementary Table 6

Supplementary Table 7

Supplementary Table 8
