# Supplementary material for: Quantitative Persulfide Site Identification (qPerS-SID) Reveals Protein Targets of H2S Releasing Donors in Mammalian Cells
Source: Sci Rep. 2016 Jul 14;6:29808. doi: 10.1038/srep29808 (PMC4944133; doi:10.1038/srep29808)
Supplement: Supplementary Information [file srep29808-s1.pdf]

Quantitative Persulfide Side Identification (qPerS-SID) Reveals Protein Targets of H_2S Releasing Donors in Mammalian Cells

Sebastian Longen*§[1], Florian Richter*[2], Yvette Köhler[1], Ilka Wittig[2], Karl-Friedrich Beck[1] and Josef Pfeilschifter[1]

SUPPLEMENTARY INFORMATION

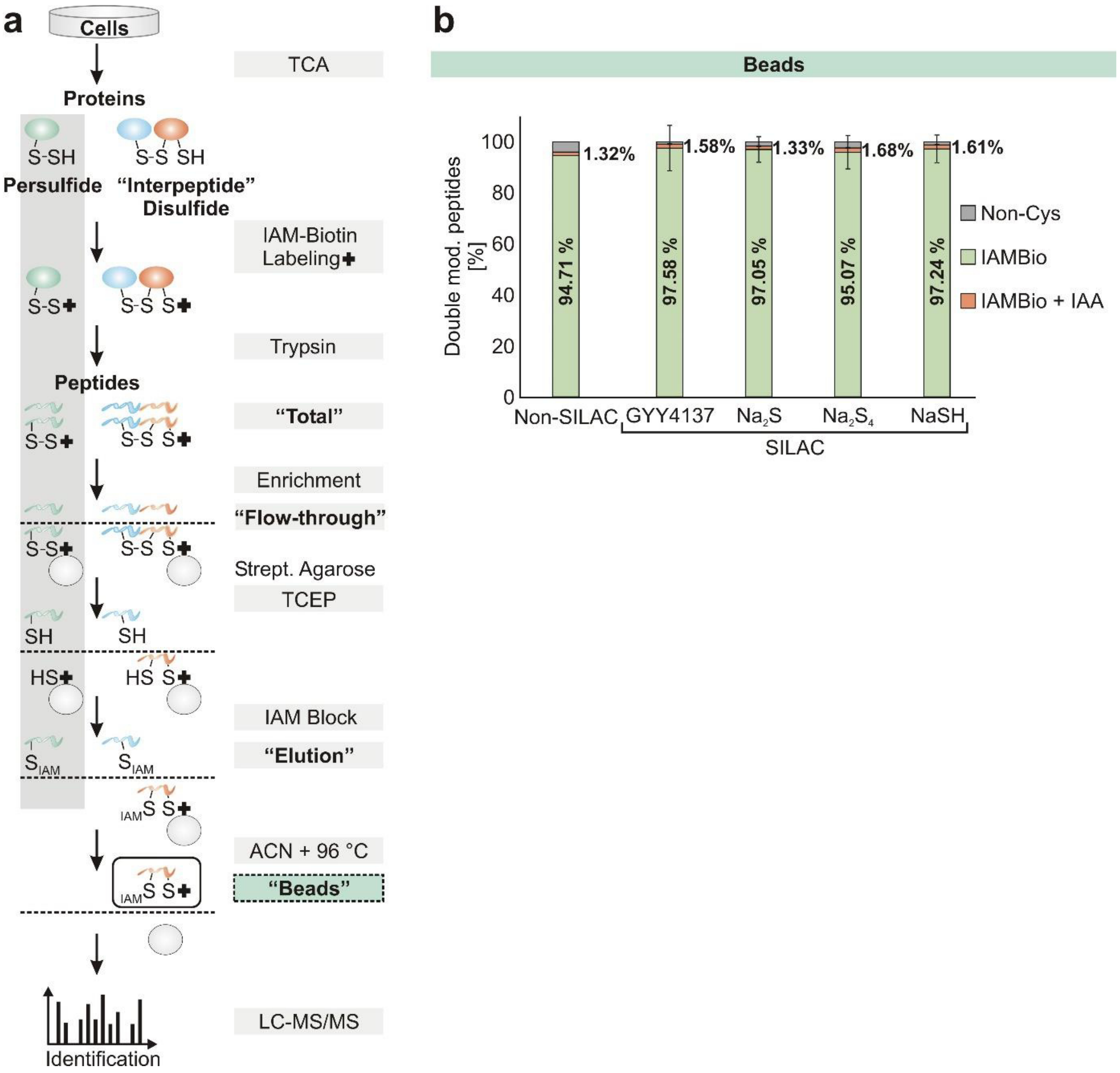

Fig S1: Evaluation of possible false positive persulfides. a) Scheme showing a special case, which might lead to the detection of false positive peptides. After digestion, two peptides connected by a disulfide bond might occur ("interpeptide disulfide"; orange and blue peptide). If in addition one of

these peptides contains at least an additional cysteine previously labelled with IAMBio (orange peptide, +), this interconnected peptide would be susceptible to TCEP treatment resulting in an elution of a non-persulfide peptide and thus, resulting in detection of a false positive peptide (blue peptide). As an indirect marker of such a case, peptides with at least two cysteines that contain both an IAMBio and IAM label can be measured in the beads fraction. **b)** Mean values of double modified peptides in the beads fraction of the experiment as described in Fig 1 (Non-SILAC, two independent experiments) and as described in Fig 2 using the H_2S donors GYY4137, Na_2S, NASH and Na_2S_4 (SILAC, four independent experiments). Error bars indicate the standard deviation in the SILAC samples. IAMBio: iodoacetyl-PEG2-Biotin; IAM: iodoacetamide. Note that the actual value of false positive peptides might be lower because the double labelling can also appear when a persulfide or another reversible thiol oxidation is present instead of an "interpeptide disulfide bond".

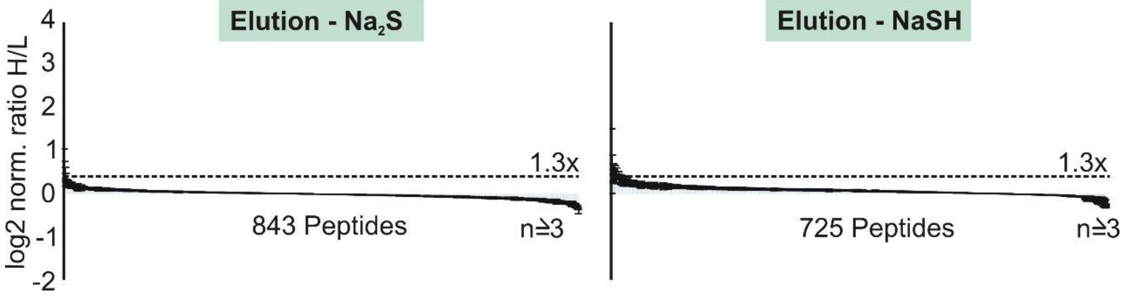

Fig S2: Quantitative changes of persulfides after treatment with 200 µM NaSH and Na_2S. The experiment was performed as described in Fig 2A, except that NaSH and Na_2S were used instead of GYY4137 and Na_2S_4. The experiment was done four times (two times forward, two times reversed) and the mean values and error bars of peptides appearing in at least 3 of 4 experiments are plotted as the log2 value. For normalisation the median of the heavy to light ratio (H/L) of non-cysteine peptides in the total fraction were used and multiplied with the measured H/L ratios. Dashed line: threshold of peptides that were at least 30 % induced (1.3x). The cells were grown in heavy or light SILAC medium allowing the quantification of induced persulfides.

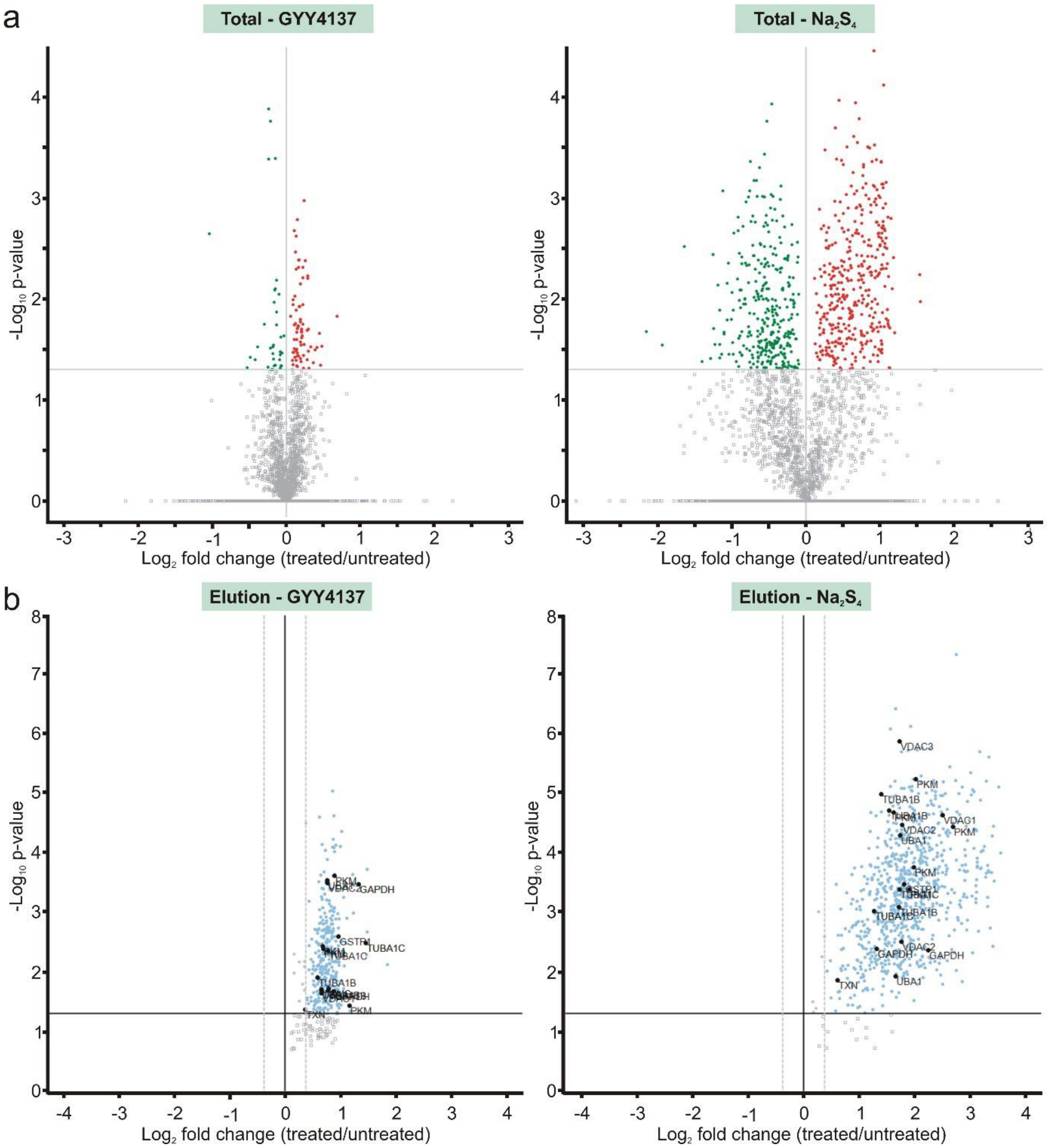

Fig S3: Statistical evaluation of the peptides identified in the total and elution fraction of GYY4137 and Na_2S_4 treated cells. One sample students *t*-test of the log_2-normalised ratios containing all peptides identified in the total fraction excluding cysteine containing peptides **(a)** and the TCEP elution fraction **(b)**. The $-log_{10}$ p-values were plotted against the log_2 mean treated/untreated ratio. Red/green dots: Peptides significantly changed in the total fraction; blue: Peptides in samples of the elution fraction identified in at least 3 of 4 experiments and significantly enriched at least 1.3 fold; grey dashed line: log_2 change of +/- 1.3; black: Peptides/proteins that were further discussed in the

paper. Note that the significant peptides in volcano plots of the elution fraction have a clear orientation to the right side because this fraction is highly enriched by our qPerS-SID strategy.

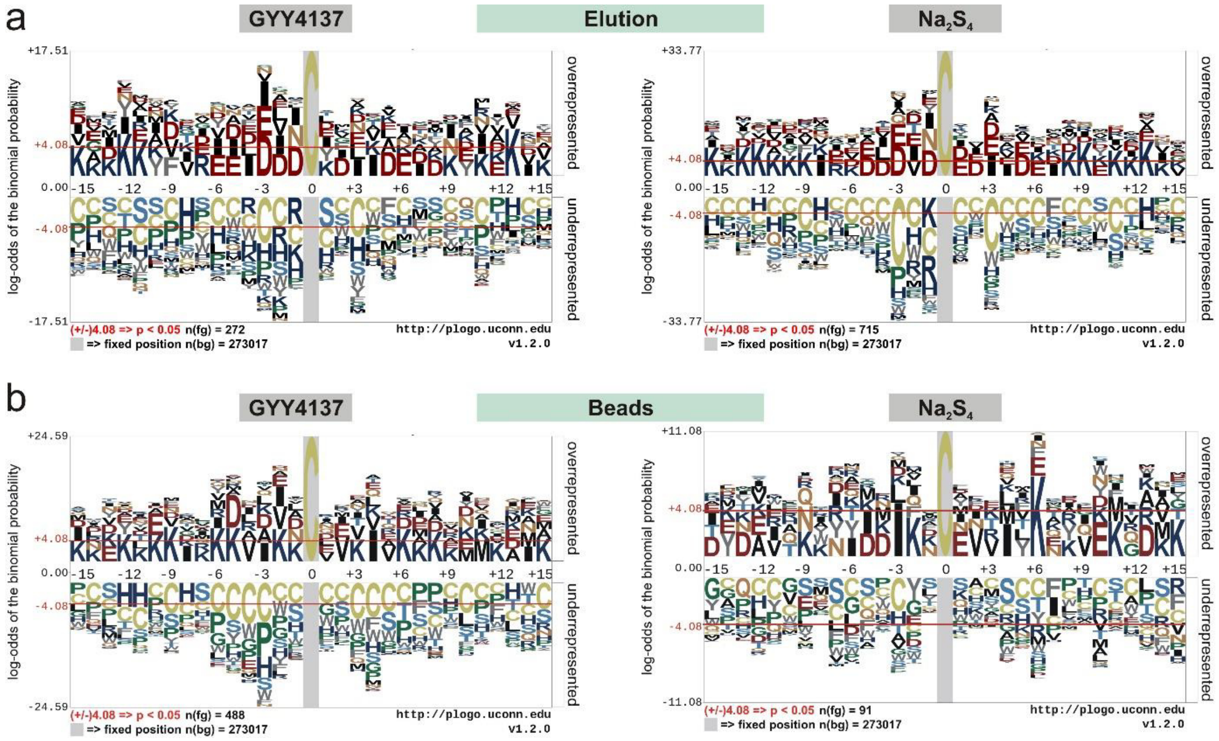

Fig S4: Amino acid enrichment analysis of persulfide containing peptides induced by GYY4137 and Na_2S_4. a) pLogo[24] analysis of enriched amino acids 15 positions before or after the reactive cysteine. Red: negative charged amino acids, blue: positive charged amino acids, black: hydrophobic amino acids. **b)** pLogo analysis of enriched amino acids 15 positions before or after cysteines not affected by H_2S stimulation found at the beads fraction. Red: negative charged amino acids, blue: positive charged amino acids, black: hydrophobic amino acids.

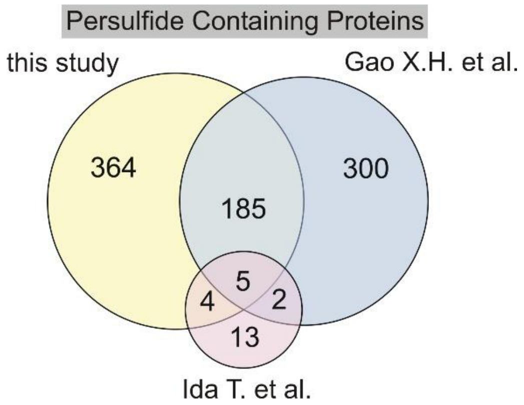

Fig S5: Comparison of different persulfide proteomes. Venn diagram comparing proteins that form persulfides identified to by our approach, a similar strategy invented by Gao X.H. et al. and the tag-switch method applied by Ida T. et al. Note that for the study of Gao X.H. et al. and our study proteins were considered forming persulfides, if the respective peptides were enriched 1.3 fold or more.

[Fig S6 a] PKM2_HUMAN (Carbamidomethyl, qPerS-SID, GYY4137)

NTGIIC[49](+57.02)TIGPASR

#	b	b (2+)	Seq	y	y (2+)	#
1	115.05	58.03	N			13
2	216.10	108.55	T	1245.66	623.33	12
3	273.12	137.06	G	1144.61	572.81	11
4	386.20	193.60	I	1087.59	544.30	10
5	499.28	250.14	I	974.51	487.75	9
6	659.32	330.16	C(+57.02)	861.42	431.21	8
7	760.37	380.68	T	701.39	351.20	7
8	873.45	437.23	I	600.35	300.67	6
9	930.48	465.74	G	487.26	244.13	5
10	1027.52	514.26	P	430.24	215.62	4
11	1098.56	549.78	A	333.19	167.09	3
12	1185.59	593.30	S	262.15	131.58	2
13			R	175.12	88.06	1

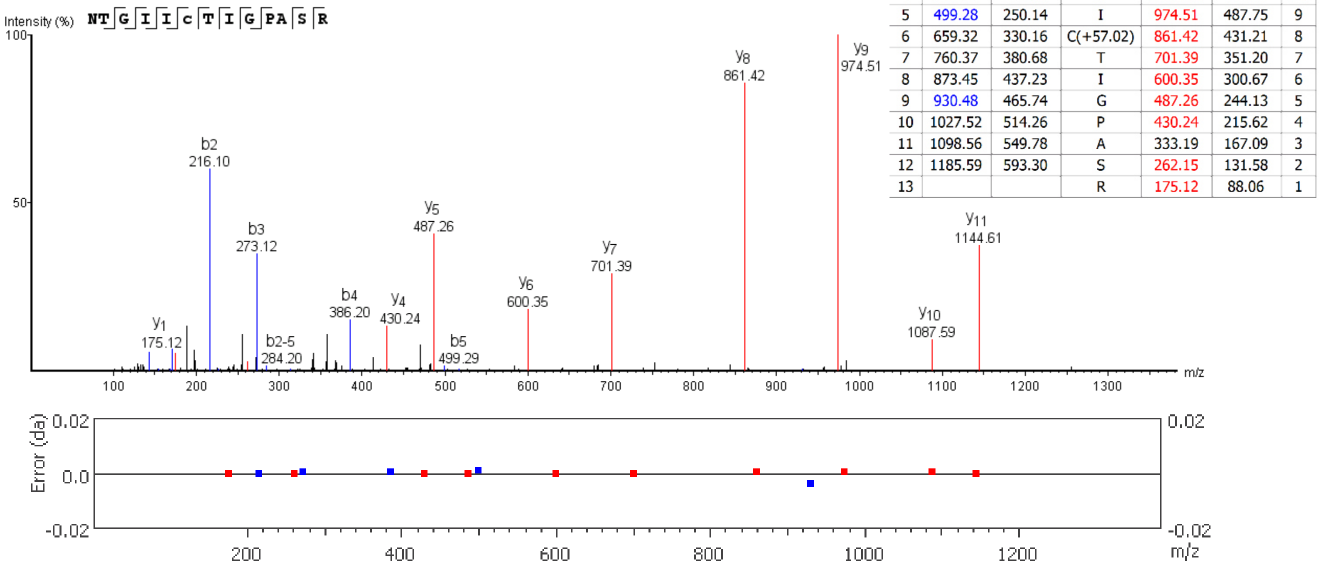

[Fig S6 b] PKM2_HUMAN (Carbamidomethyl, qPerS-SID, Na_2S_4)

NTGIIC[49](+57.02)TIGPASR

#	b	b (2+)	Seq	y	y (2+)	#
1	115.05	58.03	N			13
2	216.10	108.55	T	1245.66	623.33	12
3	273.12	137.06	G	1144.61	572.81	11
4	386.20	193.60	I	1087.59	544.30	10
5	499.29	250.14	I	974.51	487.75	9
6	659.32	330.16	C(+57.02)	861.42	431.21	8
7	760.37	380.68	T	701.39	351.20	7
8	873.45	437.23	I	600.35	300.67	6
9	930.48	465.74	G	487.26	244.13	5
10	1027.52	514.26	P	430.24	215.62	4
11	1098.56	549.78	A	333.19	167.09	3
12	1185.59	593.30	S	262.15	131.58	2
13			R	175.12	88.06	1

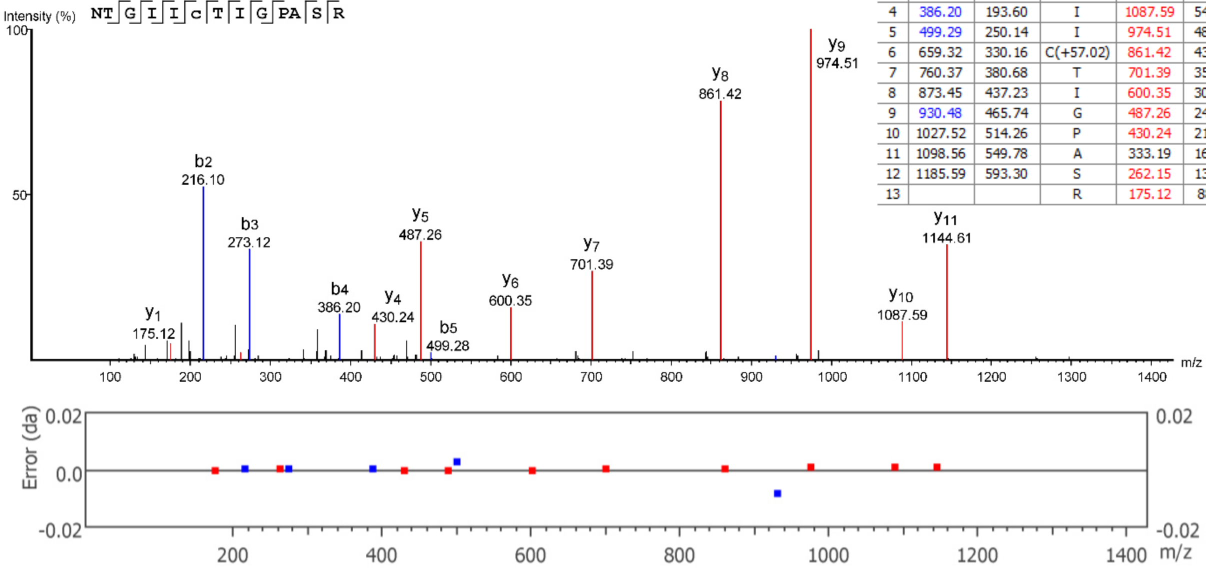

[Fig S6 c] PKM2_RABBIT (Carbamidomethyl, TCEP Elution, 500 µM Na_2S_4)

NTGIIC[49](+57.02)TIGPASR

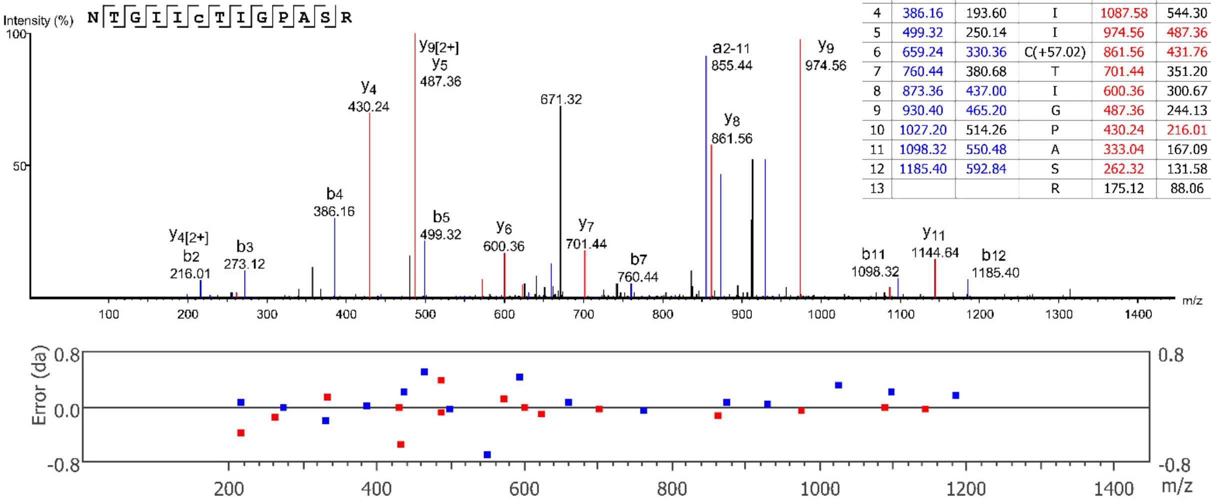

#	b	b (2+)	Seq	y	y (2+)	#
1	115.05	58.03	N			13
2	216.01	108.55	T	1245.66	623.44	12
3	273.12	137.06	G	1144.64	572.68	11
4	386.16	193.60	I	1087.58	544.30	10
5	499.32	250.14	I	974.56	487.36	9
6	659.24	330.36	C(+57.02)	861.56	431.76	8
7	760.44	380.68	T	701.44	351.20	7
8	873.36	437.00	I	600.36	300.67	6
9	930.40	465.20	G	487.36	244.13	5
10	1027.20	514.26	P	430.24	216.01	4
11	1098.32	550.48	A	333.04	167.09	3
12	1185.40	592.84	S	262.32	131.58	2
13			R	175.12	88.06	1

[Fig S6 d] PKM2_RABBIT (S-iodoTMT, TMT-Elution, 500 µM Na_2S_4)

NTGIIC[49](+356.19)TIGPASR

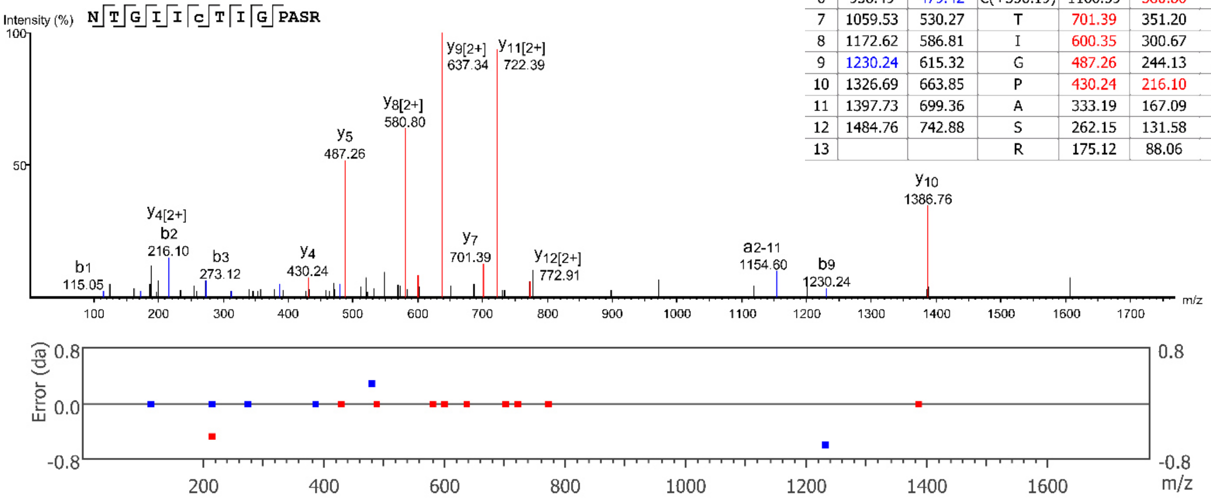

#	b	b (2+)	Seq	y	y (2+)	#
1	115.05	58.03	N			13
2	216.10	108.55	T	1544.83	772.91	12
3	273.12	137.06	G	1443.78	722.39	11
4	386.20	193.60	I	1386.76	693.88	10
5	499.29	250.14	I	1273.68	637.34	9
6	958.49	479.42	C(+356.19)	1160.59	580.80	8
7	1059.53	530.27	T	701.39	351.20	7
8	1172.62	586.81	I	600.35	300.67	6
9	1230.24	615.32	G	487.26	244.13	5
10	1326.69	663.85	P	430.24	216.10	4
11	1397.73	699.36	A	333.19	167.09	3
12	1484.76	742.88	S	262.15	131.58	2
13			R	175.12	88.06	1

[Fig S6 e] PKM2_HUMAN (Carbamidomethyl, qPerS-SID, GYY4137)

C152(+57.02)DENILWLDYK

#	b	b (2+)	Seq	y	y (2+)	#
1	161.04	81.02	C(+57.02)			11
2	276.06	138.53	D	1316.66	658.83	10
3	405.11	203.05	E	1201.63	601.32	9
4	519.15	260.08	N	1072.59	536.80	8
5	632.23	316.62	I	958.55	479.77	7
6	745.32	373.16	L	845.47	423.23	6
7	931.40	466.20	W	732.38	366.69	5
8	1044.48	522.74	L	546.30	273.65	4
9	1159.51	580.25	D	433.21	217.11	3
10	1322.57	661.79	Y	318.19	159.60	2
11			K(+8.01)	155.13	78.06	1

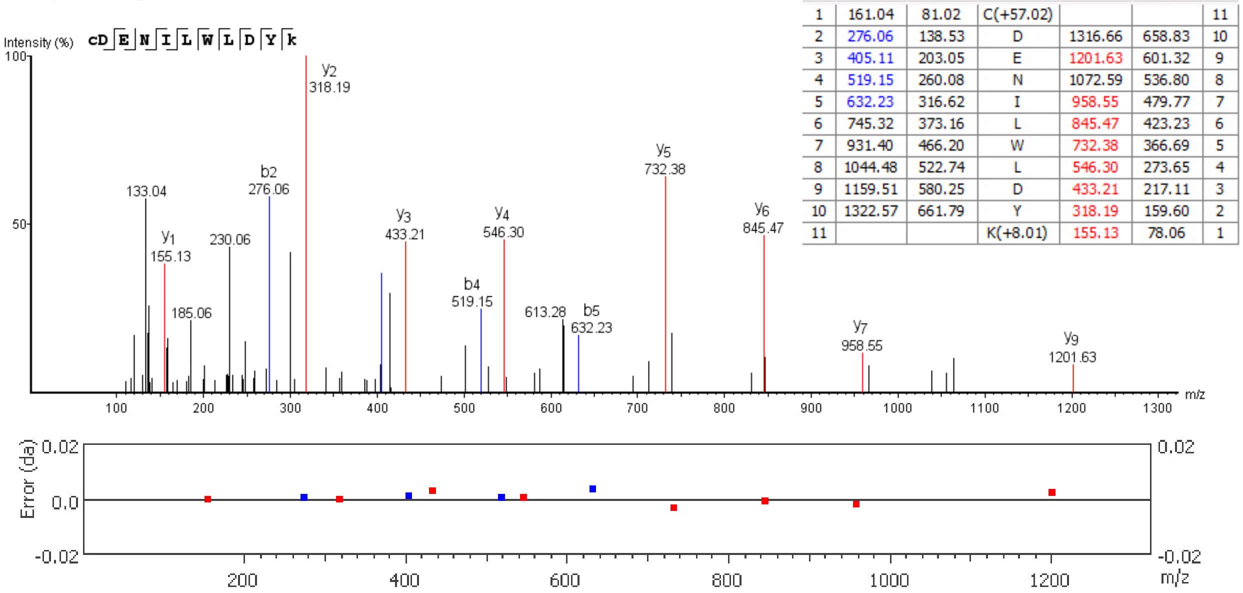

[Fig S6 f] PKM2_HUMAN (Carbamidomethyl, qPerS-SID, Na_2S_4)

C152(+57.02)DENILWLDYK

#	b	b (2+)	Seq	y	y (2+)	#
1	161.04	81.02	C(+57.02)			11
2	276.06	138.53	D	1308.65	654.82	10
3	405.11	203.05	E	1193.62	597.31	9
4	519.15	260.08	N	1064.58	532.79	8
5	632.24	316.62	I	950.53	475.77	7
6	745.32	373.16	L	837.44	419.23	6
7	931.40	466.20	W	724.37	362.68	5
8	1044.48	522.74	L	538.29	269.64	4
9	1159.51	580.25	D	425.20	213.10	3
10	1322.57	661.79	Y	310.18	155.59	2
11			K	147.11	74.06	1

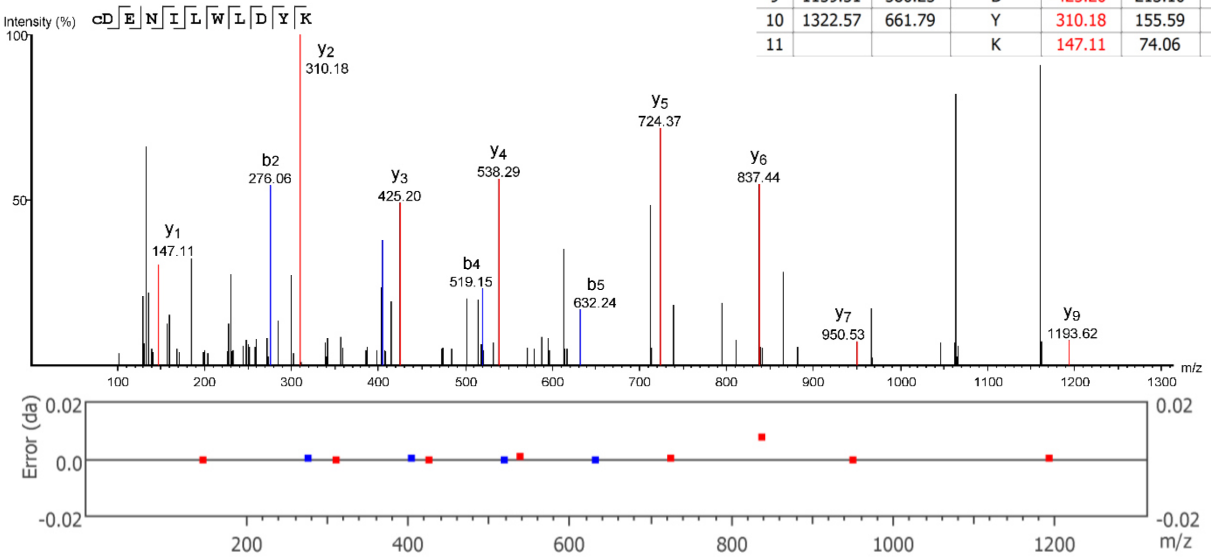

[Fig S6 g] PKM2_RABBIT (Carbamidomethyl, TCEP Elution, 500 µM Na_2S_4)

C152(+57.02)DENILWLDYK

#	b	b (2+)	Seq	y	y (2+)	#
1	161.04	81.02	C(+57.02)			11
2	275.96	138.53	D	1308.56	654.52	10
3	405.00	203.05	E	1193.72	597.24	9
4	519.08	259.96	N	1064.68	532.80	8
5	632.20	316.62	I	950.52	475.77	7
6	745.28	373.16	L	837.56	419.23	6
7	931.40	466.20	W	724.36	362.68	5
8	1044.28	522.31	L	538.32	269.64	4
9	1159.36	580.60	D	425.32	213.10	3
10	1322.44	661.79	Y	310.12	155.59	2
11			K	147.11	74.06	1

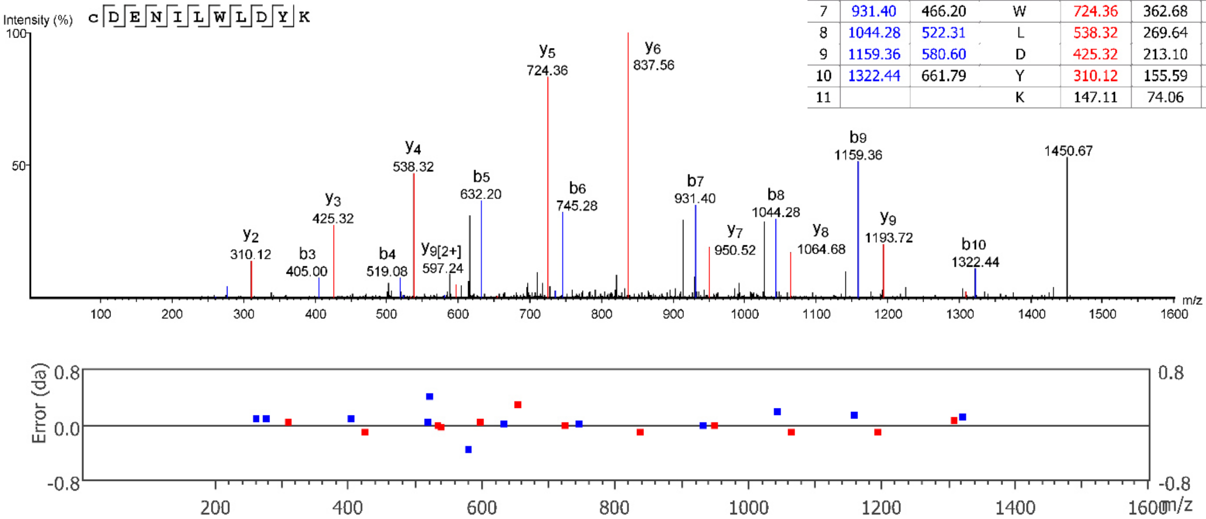

[Fig S6 h] PKM2_RABBIT (S-iodoTMT, TMT Elution, 100 µM Na_2S_4)

C152(+356.19)DENILWLDYK

#	b	b (2+)	Seq	y	y (2+)	#
1	460.92	231.40	C(+356.19)			11
2	575.28	288.12	D	1308.65	654.76	10
3	704.24	352.84	E	1193.62	597.40	9
4	818.44	409.68	N	1064.56	532.80	8
5	931.47	466.20	I	950.54	475.12	7
6	1044.28	522.74	L	837.56	419.32	6
7	1230.51	615.78	W	724.44	362.68	5
8	1343.47	672.32	L	538.20	269.64	4
9	1458.51	729.04	D	425.24	213.10	3
10	1621.71	811.37	Y	310.21	155.59	2
11			K	147.11	74.06	1

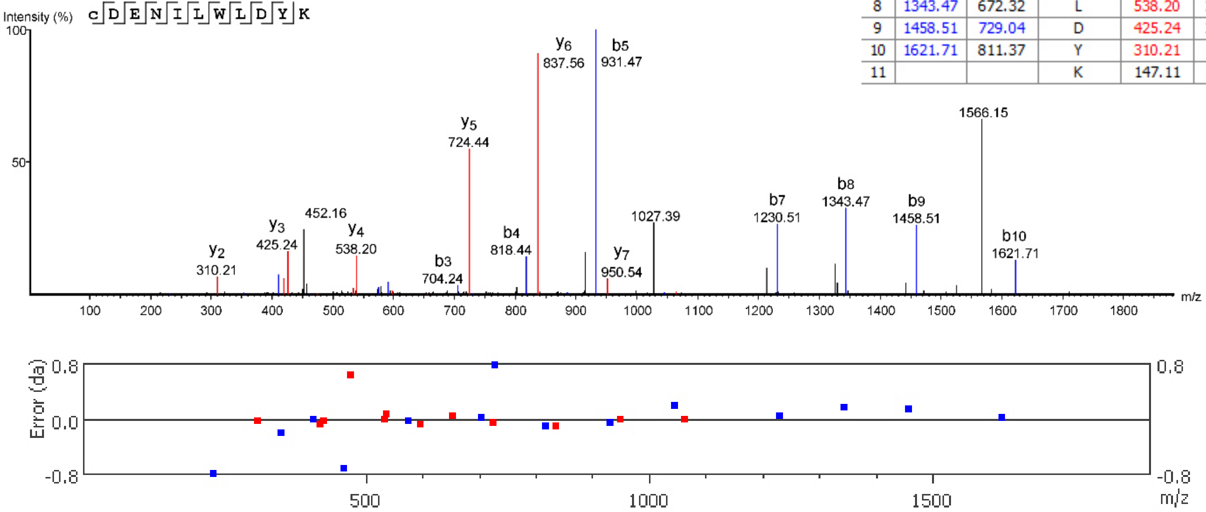

[Fig S6 i] PKM2_HUMAN (Carbamidomethyl, qPerS-SID, GYY4137)

AEGSDVANAVLDGADC^{358}(+57.02)IMLSGETAK

#	b	b (2+)	Seq	y	y (2+)	#
1	72.04	36.52	A			25
2	201.09	101.04	E	2423.11	1212.05	24
3	258.11	129.55	G	2294.06	1147.53	23
4	345.14	173.07	S	2237.04	1119.02	22
5	460.17	230.58	D	2150.01	1075.51	21
6	559.24	280.12	V	2034.98	1017.99	20
7	630.28	315.64	A	1935.90	968.46	19
8	744.32	372.66	N	1864.88	932.94	18
9	815.36	408.18	A	1750.83	875.92	17
10	914.43	457.71	V	1679.81	840.40	16
11	1027.51	514.25	L	1580.74	790.86	15
12	1142.53	571.77	D	1467.64	734.32	14
13	1199.55	600.28	G	1352.62	676.81	13
14	1270.59	635.80	A	1295.60	648.30	12
15	1385.62	693.31	D	1224.56	612.78	11
16	1545.65	773.32	C(+57.02)	1109.53	555.27	10
17	1658.73	829.87	I	949.52	475.25	9
18	1789.77	895.39	M	836.42	418.71	8
19	1902.86	951.93	L	705.38	353.19	7
20	1989.89	995.44	S	592.29	296.65	6
21	2046.91	1023.96	G	505.26	253.13	5
22	2175.95	1088.48	E	448.24	224.62	4
23	2277.00	1139.00	T	319.20	160.10	3
24	2348.04	1174.52	A	218.15	109.57	2
25			K	147.11	74.06	1

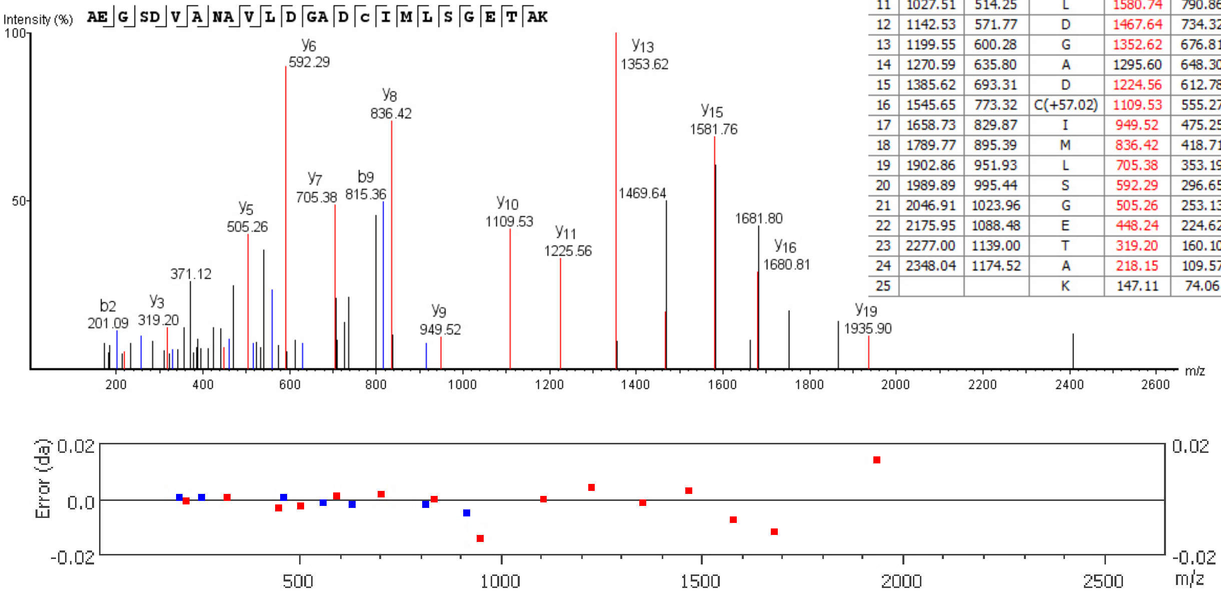

[Fig S6 j] PKM2_HUMAN (Carbamidomethyl, qPerS-SID, Na_2S_4)

AEGSDVANAVLDGADC^{358}(+57.02)IMLSGETAK

#	b	b (2+)	Seq	y	y (2+)	#
1	72.04	36.52	A			25
2	201.09	101.04	E	2423.11	1212.05	24
3	258.11	129.55	G	2294.06	1147.53	23
4	345.14	173.07	S	2237.04	1119.02	22
5	460.16	230.58	D	2150.01	1075.51	21
6	559.24	280.12	V	2034.98	1017.99	20
7	630.28	315.64	A	1935.90	968.46	19
8	744.32	372.66	N	1864.88	932.94	18
9	815.35	408.18	A	1750.83	875.92	17
10	914.42	457.71	V	1679.79	840.40	16
11	1027.51	514.25	L	1580.73	790.86	15
12	1142.53	571.77	D	1467.64	734.32	14
13	1199.55	600.28	G	1352.62	676.81	13
14	1270.59	635.80	A	1295.61	648.30	12
15	1385.62	693.31	D	1224.56	612.78	11
16	1545.65	773.32	C(+57.02)	1109.54	555.27	10
17	1658.73	829.87	I	949.49	475.25	9
18	1789.77	895.39	M	836.42	418.71	8
19	1902.86	951.93	L	705.38	353.19	7
20	1989.89	995.44	S	592.29	296.65	6
21	2046.91	1023.96	G	505.26	253.13	5
22	2175.95	1088.48	E	448.24	224.62	4
23	2277.00	1139.00	T	319.20	160.10	3
24	2348.04	1174.52	A	218.15	109.57	2
25			K	147.11	74.06	1

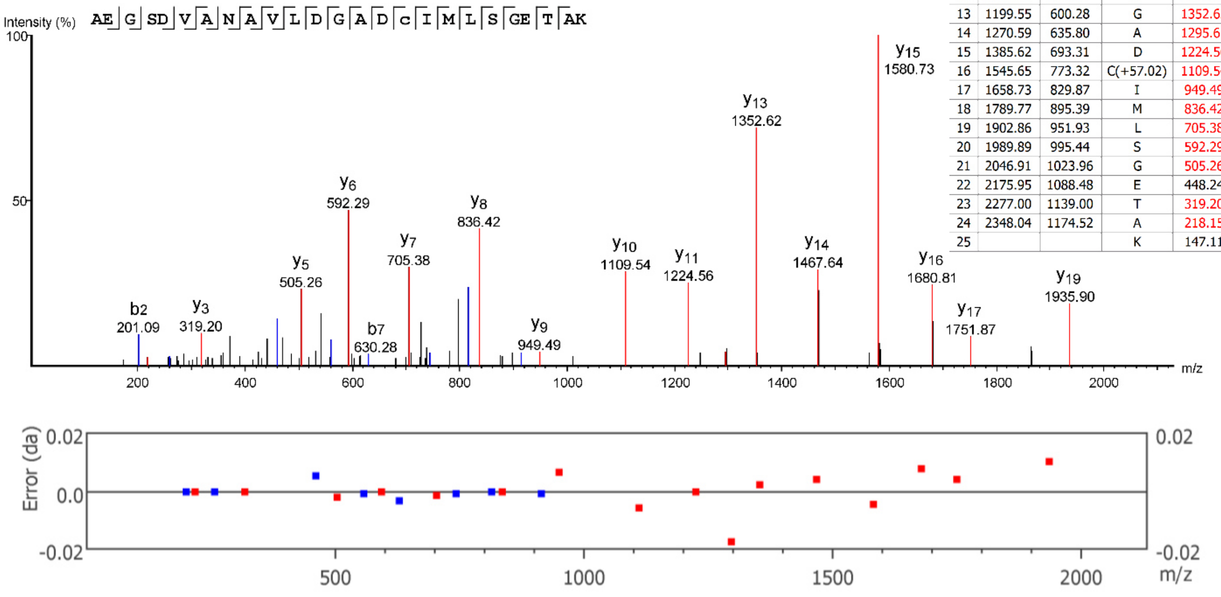

[Fig S6 k] PKM2_RABBIT (Carbamidomethyl, TCEP Elution, 500 µM Na_2S_4)

AEGSDVANAVLDGADC[358](+57.02)IMLSGETAK

#	b	b (2+)	Seq	y	y (2+)	#
1	72.04	36.52	A			25
2	201.09	101.04	E	2423.11	1212.05	24
3	258.11	129.55	G	2294.06	1147.53	23
4	345.14	173.07	S	2237.04	1119.02	22
5	460.17	230.58	D	2150.01	1075.51	21
6	559.24	280.12	V	2034.98	1017.99	20
7	630.27	315.64	A	1935.92	968.46	19
8	744.32	372.66	N	1864.88	932.94	18
9	815.35	408.18	A	1750.85	875.92	17
10	914.42	457.71	V	1679.79	840.40	16
11	1027.50	514.25	L	1580.73	790.86	15
12	1142.53	571.77	D	1467.65	734.32	14
13	1199.55	600.28	G	1352.62	676.05	13
14	1270.59	635.80	A	1295.60	648.30	12
15	1385.62	693.31	D	1224.56	612.78	11
16	1545.65	773.32	C(+57.02)	1109.53	555.27	10
17	1658.73	829.87	I	949.50	475.25	9
18	1789.77	895.39	M	836.41	418.71	8
19	1902.86	951.93	L	705.37	353.19	7
20	1989.89	995.44	S	592.29	296.65	6
21	2046.91	1023.96	G	505.26	253.13	5
22	2175.95	1088.48	E	448.24	224.62	4
23	2277.00	1139.00	T	319.20	160.10	3
24	2348.04	1174.52	A	218.15	109.57	2
25			K	147.11	74.06	1

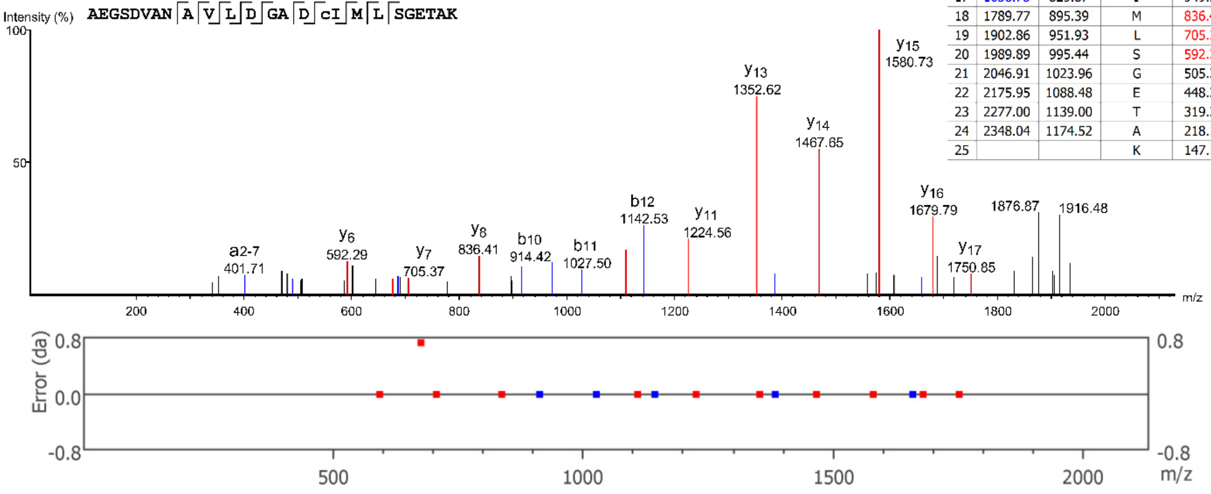

[Fig S6 l] PKM2_RABBIT (S-iodoTMT, TMT Elution, 100 µM Na_2S_4)

AEGSDVANAVLDGADC[358](+356.19)IMLSGETAK

#	b	b (2+)	Seq	y	y (2+)	#
1	72.04	36.52	A			25
2	201.09	101.04	E	2722.27	1361.44	24
3	258.11	129.55	G	2593.11	1297.15	23
4	345.00	173.07	S	2536.21	1268.56	22
5	460.12	230.58	D	2449.18	1225.08	21
6	559.24	279.60	V	2334.15	1167.72	20
7	630.44	315.64	A	2235.08	1118.04	19
8	744.24	372.66	N	2164.04	1082.60	18
9	815.52	408.18	A	2050.00	1025.60	17
10	914.52	457.71	V	1978.96	989.96	16
11	1027.40	514.56	L	1880.00	940.57	15
12	1142.40	571.77	D	1766.76	883.92	14
13	1199.60	600.28	G	1652.52	826.36	13
14	1270.36	636.24	A	1594.24	797.32	12
15	1385.40	693.31	D	1523.64	762.44	11
16	1844.64	922.91	C(+356.19)	1408.75	704.28	10
17	1957.90	979.60	I	949.56	475.25	9
18	2088.94	1044.88	M	836.44	418.71	8
19	2202.02	1101.52	L	705.92	353.08	7
20	2289.06	1144.88	S	592.40	296.65	6
21	2346.08	1173.84	G	505.36	253.13	5
22	2475.12	1237.84	E	448.20	224.62	4
23	2576.17	1288.52	T	319.20	160.10	3
24	2647.21	1323.93	A	218.15	109.57	2
25			K	147.11	74.06	1

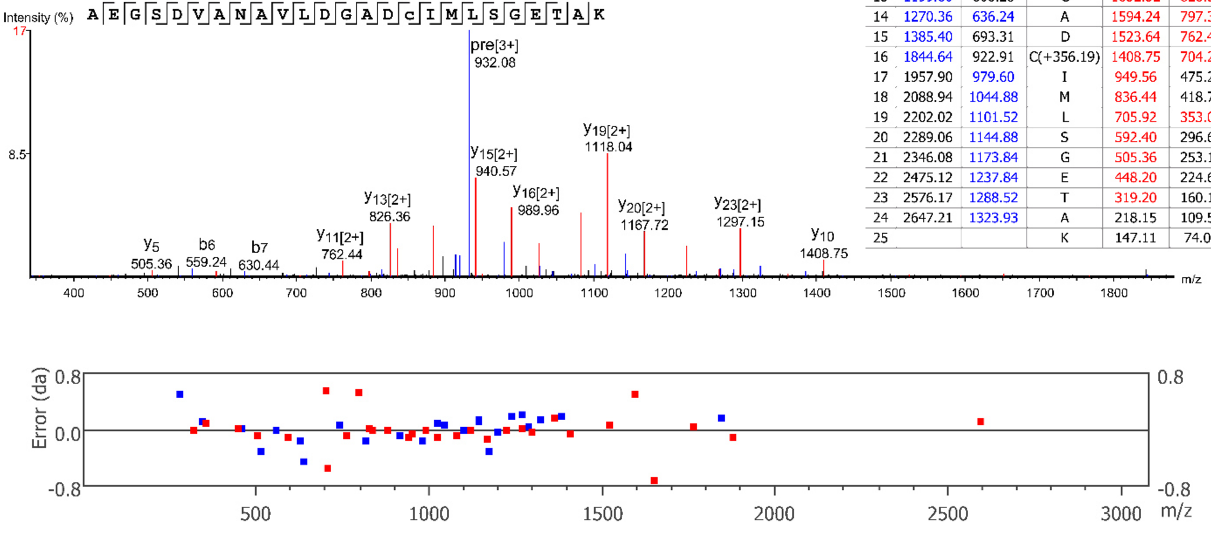

[Fig S6 m] PKM2_HUMAN (Carbamidomethyl, qPerS-SID, GYY4137)

GIFPVLC^{474}(+57.02)K

#	b	b (2+)	Seq	y	y (2+)	#
1	58.03	29.51	G			8
2	171.11	86.06	I	876.50	438.75	7
3	318.18	159.59	F	763.42	382.21	6
4	415.23	208.12	P	616.35	308.67	5
5	514.30	257.65	V	519.29	260.15	4
6	627.39	314.19	L	420.23	210.61	3
7	787.42	394.21	C(+57.02)	307.14	154.07	2
8			K	147.11	74.06	1

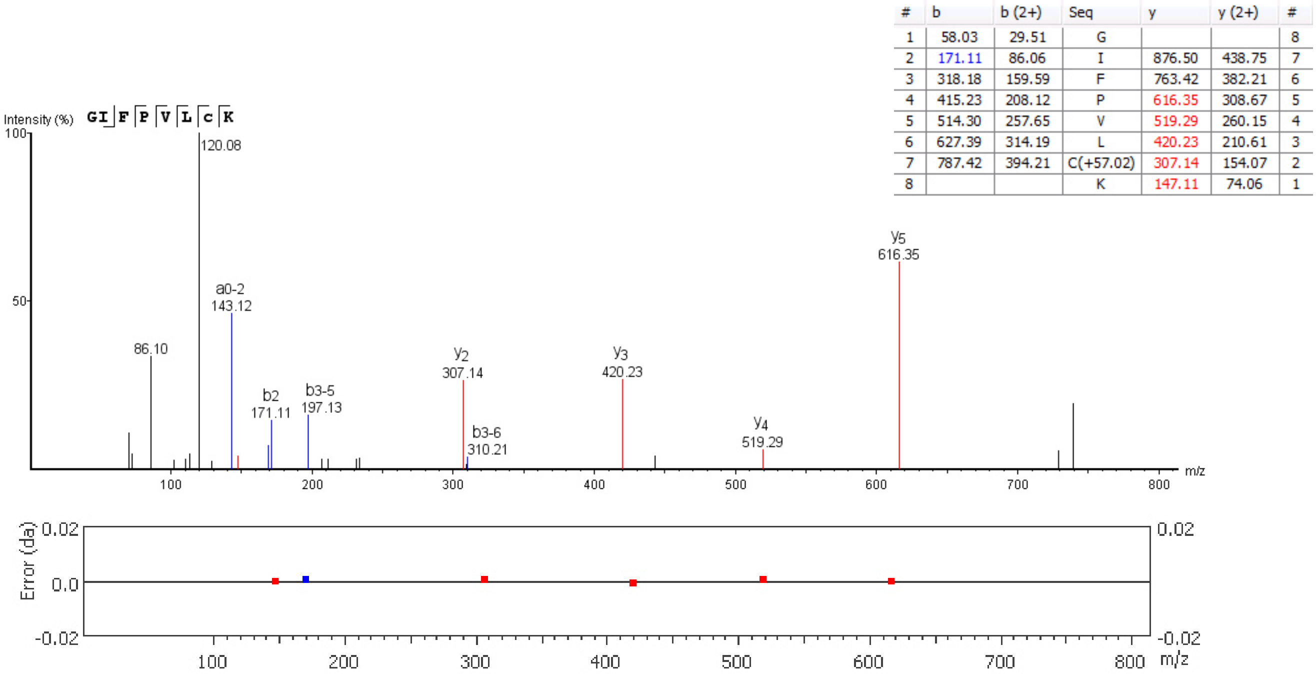

[Fig S6 n] PKM2_HUMAN (Carbamidomethyl, qPerS-SID, Na_2S_4)

GIFPVLC^{474}(+57.02)KDPVQEAWAEDVDLR

#	b	b (2+)	Seq	y	y (2+)	#
1	58.03	29.51	G			22
2	171.11	86.06	I	2500.25	1250.63	21
3	318.18	159.59	F	2387.17	1194.08	20
4	415.24	208.12	P	2240.10	1120.55	19
5	514.30	257.65	V	2143.05	1072.02	18
6	627.39	314.19	L	2043.98	1022.49	17
7	787.42	394.21	C(+57.02)	1930.90	965.94	16
8	915.51	458.26	K	1770.87	885.93	15
9	1030.55	515.77	D	1642.77	821.89	14
10	1127.59	564.30	P	1527.75	764.37	13
11	1226.66	613.83	V	1430.69	715.85	12
12	1354.72	677.86	Q	1331.63	666.31	11
13	1483.76	742.38	E	1203.56	602.28	10
14	1554.79	777.90	A	1074.52	537.76	9
15	1740.88	870.94	W	1003.48	502.24	8
16	1811.92	906.46	A	817.41	409.20	7
17	1940.96	970.98	E	746.37	373.68	6
18	2055.98	1028.49	D	617.32	309.16	5
19	2155.05	1078.03	V	502.30	251.65	4
20	2270.08	1135.54	D	403.23	202.11	3
21	2383.16	1192.08	L	288.20	144.60	2
22			R	175.12	88.06	1

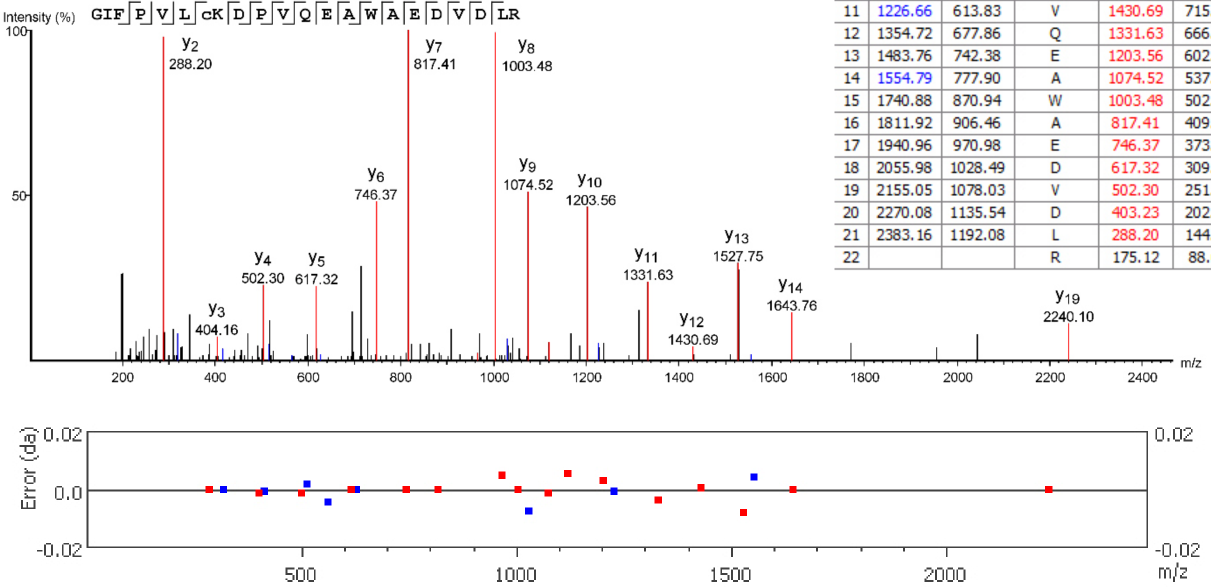

[Fig S6 o] PKM2_RABBIT (Carbamidomethyl, TCEP Elution, 500 µM Na_2S_4)

GIFPVVC474(+57.02)K

#	b	b (2+)	Seq	y	y (2+)	#
1	58.03	29.51	G			8
2	171.11	86.06	I	862.49	431.74	7
3	318.18	159.59	F	749.40	375.20	6
4	415.24	208.04	P	602.33	301.67	5
5	514.30	257.65	V	505.28	253.14	4
6	613.37	307.14	V	406.21	203.61	3
7	773.40	387.20	C(+57.02)	307.14	154.07	2
8			K	147.11	74.06	1

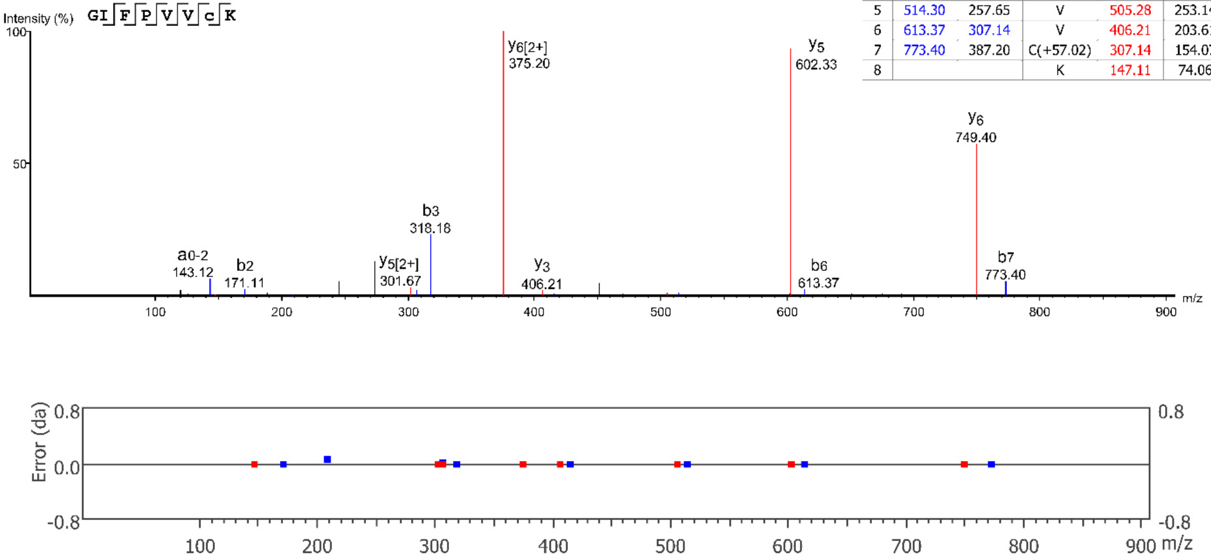

[Fig S6 p] PKM2_RABBIT (S-iodoTMT, TMT Elution, 100 µM Na_2S_4)

GIFPVVC474(+356.19)K

#	b	b (2+)	Seq	y	y (2+)	#
1	58.03	29.51	G			8
2	171.11	86.06	I	1161.65	581.33	7
3	318.18	159.59	F	1048.56	524.71	6
4	415.27	208.12	P	901.49	451.25	5
5	514.30	257.66	V	804.45	402.86	4
6	613.37	307.19	V	705.38	353.19	3
7	1072.57	536.78	C(+356.19)	606.31	303.66	2
8			K	147.11	74.06	1

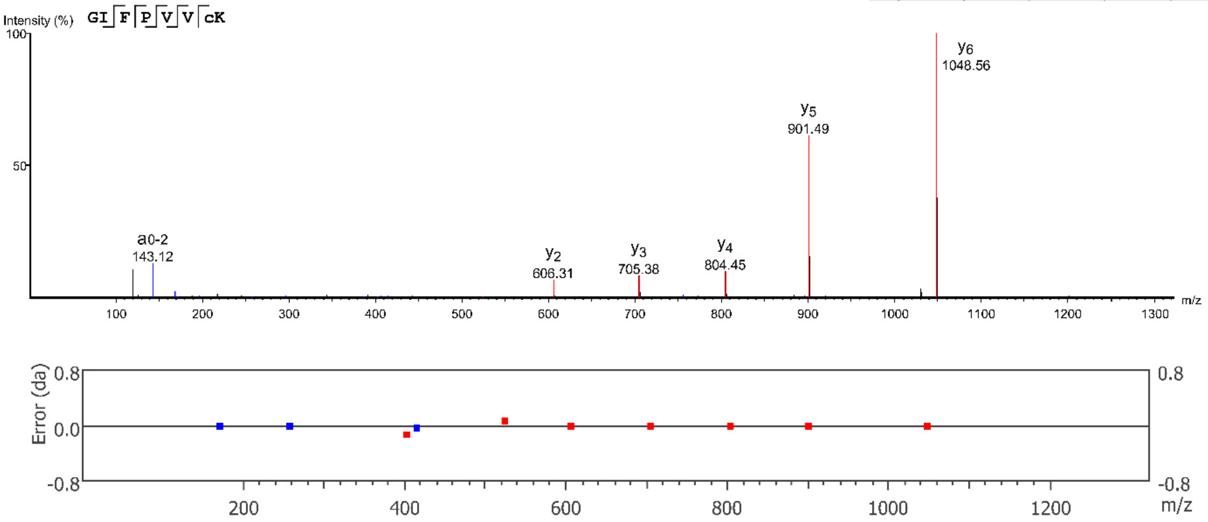

Fig S6: MS spectra of PKM2 persulfide peptides. a-p) The MS/MS fragment ions of the four identified PKM2 persulfide peptides identified in the proteomic approach (qPerS-SID, +57.02 Da) and rabbit PKM2 in the in vitro experiment (TCEP elution +57.02 Da, TMT elution +356.19 Da) are depicted in red and blue, respectively. The inset shows the sequence coverage of the peptides by b, b^{2+}, y and y^{2+} ions. Lower panels indicate the mass error.

Supplementary Figure S7

Spectrum	Gene Name	Protein Name	Peptide	Mass	ppm	m/z	z
S7.1	Q60FE5	Filamin A	ATC(+446.17)APQHGAPGPGPADASK	2177.97	7.9	727.00	3
S7.2	EF2	Elongation factor 2	C(+446.17)LYASVLTAQPR	1766.85	9.5	884.44	2
S7.3	PLST	Plastin-3	ALENDPDC(+446.17)R	1477.60	11.3	739.82	2
S7.4	HSP7C	Heat shock cognate 71 kDa protein	VC(+446.17)NPIITK	1332.66	14.0	667.35	2
S7.5	HSP74	Heat shock 70 kDa protein 4	FLEMC(+446.17)NDLLAR	1769.80	9.4	885.91	2
S7.6	PGK1	Phosphoglycerate kinase 1	DC(+446.17)VGPEVEK	1420.60	12.4	711.32	2
S7.7	DHX9	ATP-dependent RNA helicase A	AAEC(+446.17)NIVVTQPR	1745.86	10.4	873.93	2
S7.8	VIME	Vimentin	QVQSLTC(+446.17)EVDALK	1878.89	9.1	940.46	2
S7.9	MDHM	Malate dehydrogenase, mitochondrial	GC(+446.17)DVVVIPAGVPR	1726.86	11.0	864.45	2
S7.10	TIF1B	Transcription intermediary factor 1-beta	DC(+446.17)QLNAHK	1373.59	12.7	458.88	3
S7.11	ANXA2	Annexin A2	S(+42.01)TVHEILC(+446.17)K	1516.71	10.9	759.37	2
S7.12	HNRPK	Heterogeneous nuclear ribonucleoprotein K	GSDFDC(+446.17)ELR	1486.59	11.6	744.31	2
S7.13	LMNB1	Lamin-B1	C(+446.17)QSLTEDLEFR	1785.77	8.9	893.90	2
S7.14	RS3	40S ribosomal protein S3	GC(+446.17)EVVVSGK	1322.60	14.5	662.32	2
S7.15	VDAC1	Voltage-dependent anion-selective channel protein 1	YQIDPDAC(+446.17)FSAK	1802.77	9.1	902.40	2
S7.16	G3P	Glyceraldehyde-3-phosphate dehydrogenase	VPTANVSVVDLTC(+446.17)R	1918.93	11.8	960.48	2
S7.17	G3V576	Heterogeneous nuclear ribonucleoproteins C1/C2	IVGC(+446.17)SVHK	1287.61	14.0	430.22	3
S7.18	PPIA	Peptidyl-prolyl cis-trans isomerase A	GSC(+446.17)FHR	1151.47	14.8	384.84	3
S7.19	PPIA	Peptidyl-prolyl cis-trans isomerase A	IIPGFMC(+446.17)QGGDFTR	1986.88	9.4	663.31	3
S7.20	COF1	Cofilin-1	AVLFC(+446.17)LSEDK	1569.72	0.1	785.87	2
S7.21	COF1	Cofilin-1	HELQANC(+446.17)YEEVK	1907.82	7.4	954.93	2
S7.22	C9J9K3	40S ribosomal protein SA	YVDIAIPC(+446.17)NNK	1694.78	7.6	848.41	2
S7.23	HS90B	Heat shock protein HSP 90-alpha	FENLC(+446.17)K	1198.52	14.0	600.27	2
S7.24	NDKA	Nucleoside diphosphate kinase A	GDFC(+446.17)IQVGR	1439.64	11.5	720.83	2
S7.25	RS8	40S ribosomal protein S8	NC(+446.17)IVLIDSTPYR	1838.91	0.7	920.45	2
S7.26	PROF1	Profilin-1	C(+446.17)YEMASHLR	1554.65	12.1	519.23	3
S7.27	PEBP1	Phosphatidylethanolamine-binding protein 1	C(+446.17)DEPILSNR	1491.65	12.7	746.84	2
S7.28	A0A0A0MR02	Voltage-dependent anion-selective channel protein 2	WC(+446.17)EYGLTFTEK	1821.78	5.3	911.90	2
S7.29	RS16	40S ribosomal protein S16	TATAVAHC(+446.17)K	1346.61	12.4	674.32	2
S7.30	SPSY	Spermine synthase	LYC(+446.17)PVEFSK	1530.69	5.9	766.36	2
S7.31	RAN	GTP-binding nuclear protein Ran	VC(+414.19)ENIPIVLC(+446.17)GNK	2261.08	7.5	1131.55	2
S7.32	SMD2	Small nuclear ribonucleoprotein Sm D2	NNTQVLIN(+446.17)CR	1619.78	-3.9	810.89	2
S7.33	G3V203	60S ribosomal protein L18	GC(+446.17)GTVLLSGPR	1504.72	13.5	753.38	2
S7.34	RL13A	60S ribosomal protein L13a	C(+446.17)EGINISGNFYR	1817.79	8.3	909.91	2
S7.35	CNBP	Cellular nucleic acid-binding protein	C(+446.17)GESGHLAR	1374.58	11.4	459.21	3
S7.36	RL12	60S ribosomal protein L12	C(+446.17)TGGEVGATSALAPK	1806.83	11.0	904.43	2
S7.37	FETUA	Alpha-2-HS-glycoprotein	C(+446.17)DSSPDSAEDVR	1725.66	0.7	863.84	2
S7.38	MIF	Macrophage migration inhibitory factor	LLC(+446.17)GLLAER	1432.72	11.7	717.38	2
S7.39	A0A0C4DGL3	DUTP pyrophosphatase, isoform CRA_c	IAQLIC(+446.17)ER	1390.68	13.9	696.36	2
S7.40	HSP71	Heat shock 70 kDa protein 1A/1B	FEELC(+446.17)SDLFR	1703.74	8.5	852.88	2
S7.41	HNRPM	Heterogeneous nuclear ribonucleoprotein M	GC(+446.17)AVVEFK	1297.59	13.1	649.81	2
S7.42	PLST	Plastin-3	AVGDGIVLC(+446.17)K	1419.69	10.0	710.86	2
S7.43	PARP1	Poly [ADP-ribose] polymerase 1	VC(+446.17)STNDLK	1324.58	11.6	663.31	2
S7.44	KPYM	Pyruvate kinase PKM	NTGIIC(+446.17)TIGPASR	1747.84	11.5	874.94	2
S7.45	KPYM	Pyruvate kinase PKM	C(+446.17)C(+414.19)SGAIIVLTK	1966.94	7.9	984.49	2
S7.46	UCHL1	Ubiquitin carboxyl-terminal hydrolase isozyme L1	FSAVALC(+446.17)K	1283.61	13.5	642.82	2
S7.47	TERA	Transitional endoplasmic reticulum ATPase	LGDVISIQPC(+446.17)PDVK	1928.94	8.9	965.49	2
S7.48	RL11	60S ribosomal protein L11	IAVHC(+446.17)TVR	1343.65	13.9	672.84	2
S7.49	MCM3	DNA replication licensing factor MCM3	C(+446.17)SVLAAANPVYGR	1765.83	8.1	883.93	2
S7.50	MDHM	Malate dehydrogenase, mitochondrial	EGVVEC(+446.17)SFVK	1541.69	11.5	771.86	2
S7.51	NASP	Nuclear autoantigenic sperm protein	SVSGTDVQEEC(+446.17)R	1754.73	9.7	878.38	2
S7.52	EF1A3	Putative elongation factor 1-alpha-like 3	SGDAAIVDMVPGKPMC(+446.17)VESFSDYPPLGR	3383.54	-0.8	1128.85	3
S7.53	HNRPK	Heterogeneous nuclear ribonucleoprotein K	LFQEC(+446.17)C(+414.19)PHSTDR	2294.96	7.7	766.00	3
S7.54	STIP1	Stress-induced-phosphoprotein 1	ALDLDSSC(+446.17)K	1396.60	11.6	699.32	2
S7.55	HNRPR	Heterogeneous nuclear ribonucleoprotein R	SAFLC(+446.17)GVMK	1400.63	12.9	701.33	2
S7.56	PRDX6	Peroxiredoxin-6	DFTPVC(+446.17)TTELGR	1783.79	10.2	892.91	2
S7.57	SAHH	Adenosylhomocysteinase	FDNLYGC(+446.17)R	1432.59	12.3	717.31	2
S7.58	Q5HYB6	Tropomyosin alpha-3 chain	C(+446.17)LSAAEEK	1295.56	12.5	648.79	2
S7.59	PAIRB	Plasminogen activator inhibitor 1 RNA-binding protein	PGHLQEGFGC(+446.17)VVTNR	2058.94	6.0	687.33	3
S7.60	D6RAT0	40S ribosomal protein S3a	AC(+446.17)QSIYPLHDVFVR	2092.99	8.1	698.68	3
S7.61	DEST	Destrin	C(+446.17)STPEEIKK	1479.68	9.8	494.24	3
S7.62	RS4X	40S ribosomal protein S4, X isoform	FDTGNLC(+446.17)MVTGGANLGR	2170.96	8.3	1086.50	2
S7.63	PRDX1	Peroxiredoxin-1	HGEVC(+446.17)PAGWKPGSDTIKPDVQK	2794.32	4.1	699.59	4
S7.64	HNRL2	Heterogeneous nuclear ribonucleoprotein U	EGC(+446.17)TEVSLLR	1551.71	9.9	776.87	2
S7.65	PARK7	Protein deglycase DJ-1	DVVIC(+446.17)PDASLEDAKK	2047.96	8.4	683.67	3
S7.66	PDCD4	Programmed cell death protein 4	GTVDC(+446.17)VQAR	1393.62	12.5	697.82	2
S7.67	SMD2	Small nuclear ribonucleoprotein Sm D2	HC(+446.17)NMVLENVK	1631.76	-3.8	816.88	2
S7.68	H0YA96	Heterogeneous nuclear ribonucleoprotein D0	GFC(+446.17)FITFK	1407.64	12.0	704.84	2
S7.69	GSTO1	Glutathione S-transferase omega-1	LNEC(+446.17)VDHTPK	1600.70	8.9	801.37	2
S7.70	RAC1	Ras-related C3 botulinum toxin substrate 1	AVLC(+446.17)PPPVK	1368.70	-0.6	685.36	2
S7.71	E9PJD9	60S ribosomal protein L27a	NQSFC(+446.17)PTVNLDK	1810.81	9.6	906.42	2
S7.72	E9PC52	Histone-binding protein RBBP7	HPAKPDPSGEC(+446.17)NPDLR	2177.97	9.2	727.00	3
S7.73	TEBP	Prostaglandin E synthase 3	LTFSC(+446.17)LGGSDNFK	1833.81	7.2	917.92	2
S7.74	RL36	60S ribosomal protein L36	EVC(+446.17)GFAPYER	1615.68	11.5	808.86	2
S7.75	NUCL	Nucleolin	EALNSC(+446.17)NK	1323.56	13.3	662.80	2
S7.76	ENPL	Endoplasmin	G(+42.01)YEVIYLTEPVDEYC(+446.17)IQ(+.98)ALPEFDGK	3379.52	11.9	1127.53	3
S7.77	PPIA	Peptidyl-prolyl cis-trans isomerase A	KITIADC(+446.17)GQLE	1635.80	11.6	818.90	2
S7.78	HNRPU	Heterogeneous nuclear ribonucleoprotein U	MC(+446.17)LFAGFQR	1517.49	-3.1	759.75	2

Legend	
**+42.01**	Acetylation
**+.98**	Deamidation
**+414.19**	IAMBio
**+446.17**	S-IAMBio
**Mass**	Nominal Mass without charge
**ppm**	Mass Deviation from theoretical mass
**m/z**	Mass to Charge Ratio (Precursor mass detected)
**z**	Charge of the Precursor peptide
**RT**	Retention Time
**Scan**	Scan Number in the LC-MSMSM run

Supplementary Figure S7

[S7.1] FilaminA

ATC(+446.17)APQHGAPGPGPADASK

m/z=727.0016 z=3

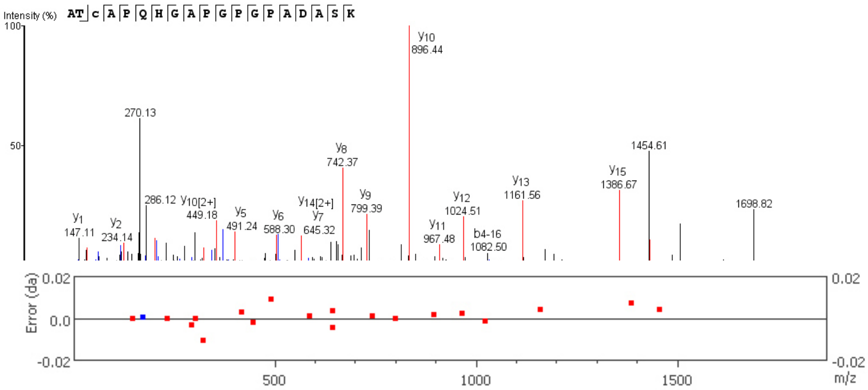

#	b	b (2+)	Seq	y	y (2+)	#
1	72.04	36.52	A			19
2	173.09	87.05	T	2107.94	1054.47	18
3	722.27	361.63	C(+446.17)	2006.89	1003.94	17
4	793.30	397.15	A	1457.71	729.36	16
5	890.36	445.68	P	1386.67	693.84	15
6	1018.42	509.71	Q	1289.62	645.32	14
7	1155.47	578.24	H	1161.56	581.28	13
8	1212.50	606.75	G	1024.51	512.75	12
9	1283.53	642.27	A	967.48	484.24	11
10	1380.59	690.79	P	896.44	448.73	10
11	1437.61	719.30	G	799.39	400.20	9
12	1534.66	767.83	P	742.37	371.69	8
13	1591.68	796.34	G	645.32	323.17	7
14	1688.73	844.87	P	588.30	294.65	6
15	1759.77	880.39	A	491.24	246.12	5
16	1874.80	937.90	D	420.21	210.60	4
17	1945.84	973.42	A	305.18	153.09	3
18	2032.87	1016.93	S	234.14	117.57	2
19			K	147.11	74.06	1

[S7.2] ElongationFactor2

C(+446.17)LYASVLTAQPR

m/z=884.4415 z=2

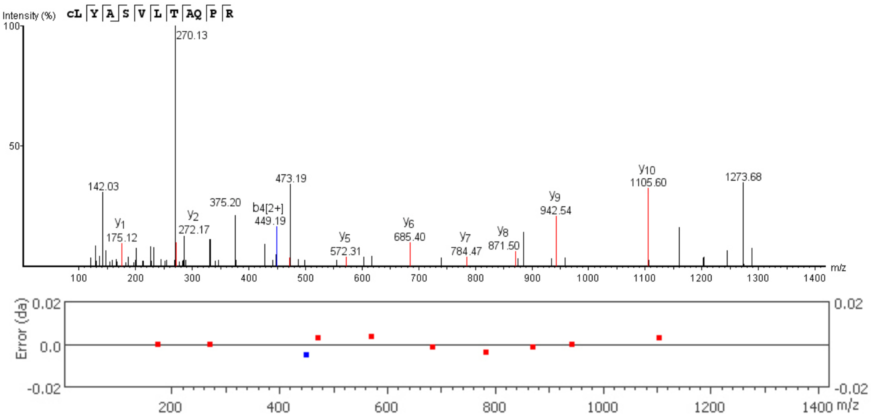

#	b	b (2+)	Seq	y	y (2+)	#
1	550.18	275.59	C(+446.17)			12
2	663.27	332.13	L	1218.68	609.84	11
3	826.33	413.67	Y	1105.60	553.30	10
4	897.37	449.19	A	942.54	471.77	9
5	984.40	492.70	S	871.50	436.25	8
6	1083.47	542.23	V	784.47	392.73	7
7	1196.55	598.78	L	685.40	343.20	6
8	1297.60	649.30	T	572.31	286.66	5
9	1368.64	684.82	A	471.26	236.13	4
10	1496.70	748.85	Q	400.23	200.62	3
11	1593.75	797.37	P	272.17	136.59	2
12			R	175.12	88.06	1

[S7.3] Plastin-3

ALENDPDC(+446.17)R

m/z=739.8156 z=2

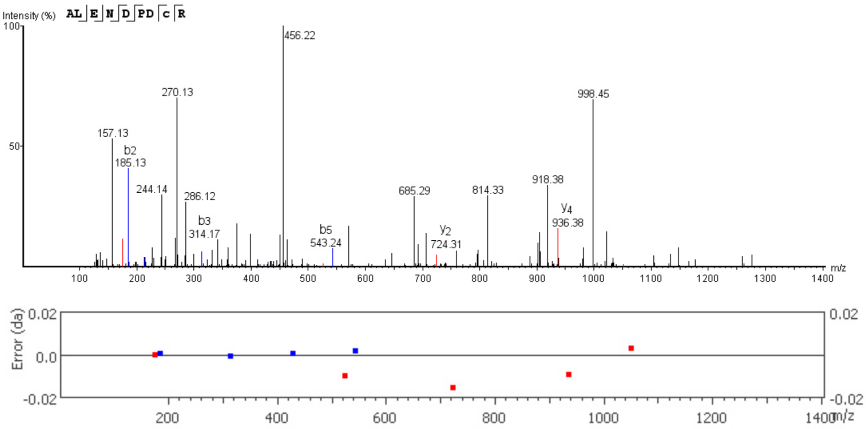

#	b	b (2+)	Seq	y	y (2+)	#
1	72.04	36.52	A			9
2	185.13	93.06	L	1407.57	704.29	8
3	314.17	157.59	E	1294.49	647.74	7
4	428.21	214.61	N	1165.44	583.22	6
5	543.24	272.12	D	1051.40	526.21	5
6	640.29	320.65	P	936.38	468.69	4
7	755.32	378.16	D	839.32	420.16	3
8	1304.50	652.75	C(+446.17)	724.31	362.65	2
9			R	175.12	88.06	1

Supplementary Figure S7

[S7.4] Heat shock cognate 71 kDa protein

VC(+446.17)NPIITK

m/z=667.3468 z=2

#	b	b (2+)	Seq	y	y (2+)	#
1	100.08	50.54	V			8
2	649.25	325.13	C(+446.17)	1234.60	617.80	7
3	763.29	382.15	N	685.42	343.21	6
4	860.35	430.68	P	571.38	286.19	5
5	973.43	487.22	I	474.33	237.66	4
6	1086.52	543.76	I	361.24	181.12	3
7	1187.56	594.28	T	248.16	124.58	2
8			K	147.11	74.06	1

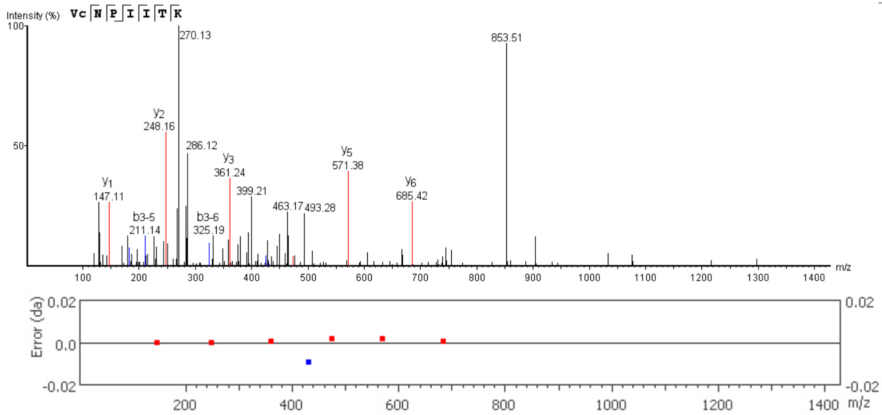

[S7.5] Heat shock 70 kDa protein 4

FLEMC(+446.17)NDLLAR

m/z=885.91425 z=2

#	b	b (2+)	Seq	y	y (2+)	#
1	148.08	74.54	F			11
2	261.16	131.08	L	1623.74	812.37	10
3	390.20	195.60	E	1510.65	755.83	9
4	521.24	261.12	M	1381.61	691.30	8
5	1070.42	535.71	C(+446.17)	1250.57	625.78	7
6	1184.46	592.73	N	701.39	351.20	6
7	1299.49	650.24	D	587.35	294.18	5
8	1412.57	706.79	L	472.32	236.66	4
9	1525.66	763.33	L	359.24	180.12	3
10	1596.69	798.85	A	246.15	123.58	2
11			R	175.12	88.06	1

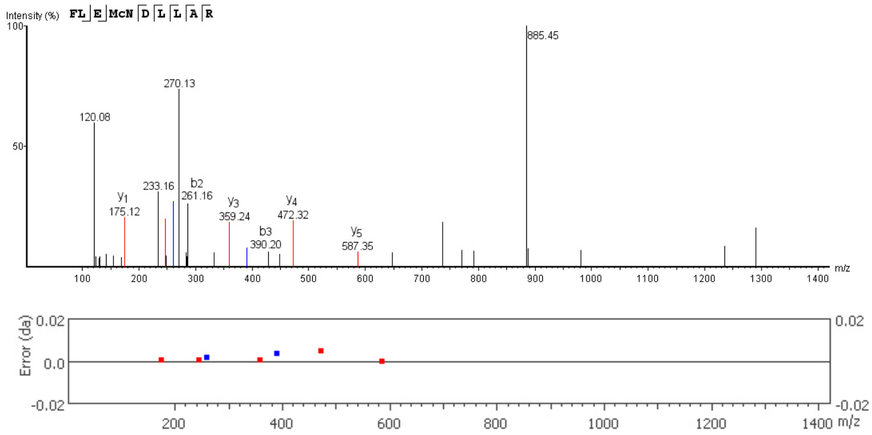

[S7.6] Phosphoglycerate kinase 1

DC(+446.17)VGPEVEK

m/z=711.3179 z=2

#	b	b (2+)	Seq	y	y (2+)	#
1	116.03	58.52	D			9
2	665.21	333.10	C(+446.17)	1306.58	653.79	8
3	764.28	382.64	V	757.41	379.20	7
4	821.30	411.17	G	658.34	329.67	6
5	918.35	459.68	P	601.32	301.16	5
6	1047.40	524.20	E	504.27	252.63	4
7	1146.46	573.73	V	375.22	188.11	3
8	1275.51	638.25	E	276.15	138.58	2
9			K	147.11	74.06	1

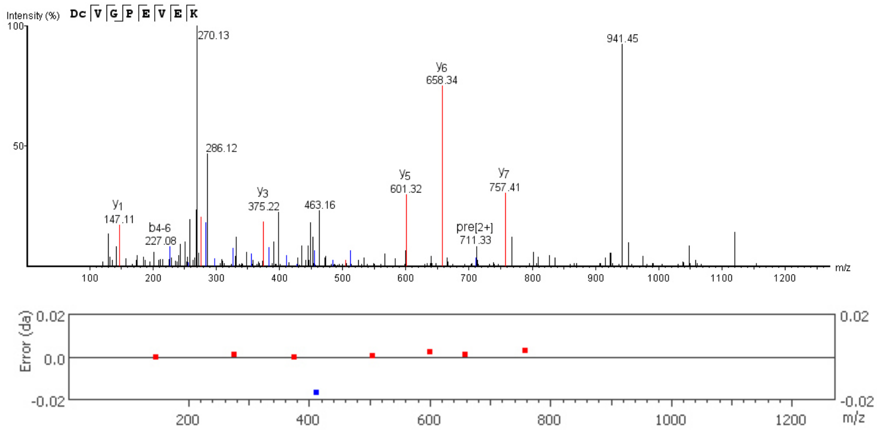

Supplementary Figure S7

[S7.7] ATP-dependent RNA helicase A

AAEC(+446.17)NIVVTQPR

m/z=873.9295 z=2

#	b	b (2+)	Seq	y	y (2+)	#
1	72.04	36.52	A			12
2	143.08	72.04	A	1675.80	838.40	11
3	272.13	136.56	E	1604.76	802.88	10
4	821.30	411.15	C(+446.17)	1475.72	738.36	9
5	935.34	468.17	N	926.54	463.77	8
6	1048.43	524.71	I	812.50	406.75	7
7	1147.49	574.25	V	699.41	350.21	6
8	1246.56	623.78	V	600.34	300.67	5
9	1347.61	674.31	T	501.28	251.14	4
10	1475.67	738.33	Q	400.23	200.62	3
11	1572.72	786.86	P	272.17	136.59	2
12			R	175.12	88.06	1

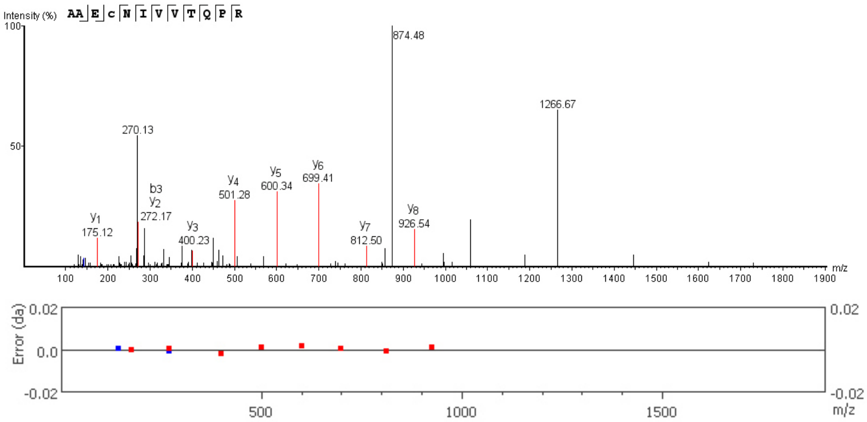

[S7.8] Vimentin

QVQSLTC(+446.17)EVDALK

m/z=940.4598 z=2

#	b	b (2+)	Seq	y	y (2+)	#
1	129.07	65.03	Q			13
2	228.13	114.57	V	1751.84	876.42	12
3	356.19	178.60	Q	1652.77	826.88	11
4	443.23	222.11	S	1524.71	762.86	10
5	556.31	278.65	L	1437.68	719.34	9
6	657.36	329.18	T	1324.59	662.80	8
7	1206.53	603.77	C(+446.17)	1223.55	612.27	7
8	1335.57	668.29	E	674.37	337.69	6
9	1434.64	717.82	V	545.33	273.16	5
10	1549.67	775.34	D	446.26	223.63	4
11	1620.71	810.85	A	331.23	166.12	3
12	1733.79	867.40	L	260.20	130.60	2
13			K	147.11	74.06	1

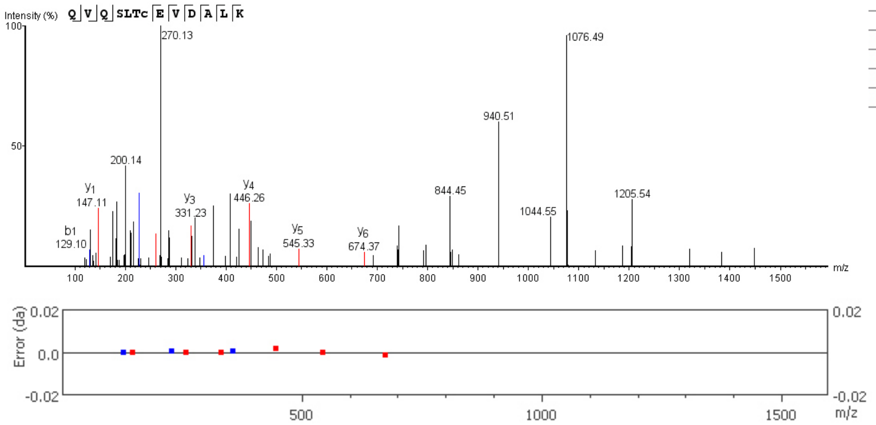

[S7.9] Malate dehydrogenase, mitochondrial

GC(+446.17)DVVVIPAGVPR

m/z=864.4452z= 2

#	b	b (2+)	Seq	y	y (2+)	#
1	58.03	29.51	G			13
2	607.20	304.10	C(+446.17)	1670.84	835.92	12
3	722.23	361.62	D	1121.67	561.33	11
4	821.30	411.17	V	1006.64	503.82	10
5	920.37	460.68	V	907.57	454.29	9
6	1019.44	510.22	V	808.50	404.75	8
7	1132.52	566.76	I	709.44	355.22	7
8	1229.57	615.29	P	596.35	298.68	6
9	1300.61	650.81	A	499.30	250.15	5
10	1357.63	679.32	G	428.26	214.63	4
11	1456.70	728.85	V	371.24	186.12	3
12	1553.75	777.38	P	272.17	136.59	2
13			R	175.12	88.06	1

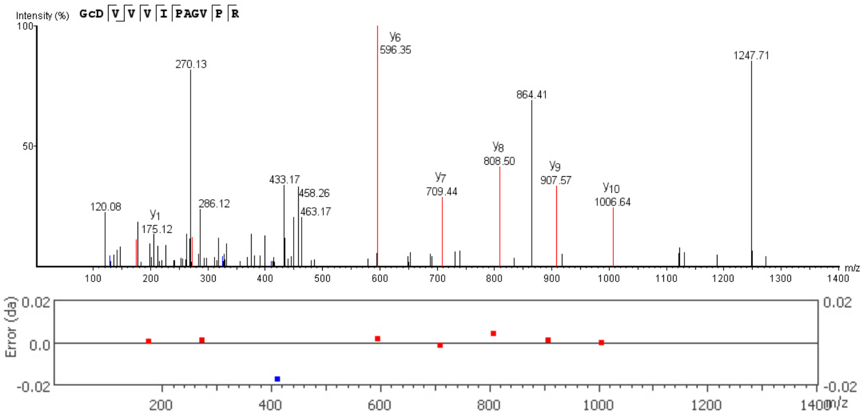

Supplementary Figure S7

[S7.10] Transcription intermediary factor 1-beta

DC(+446.17)QLNAHK

m/z=458.8761 z=3

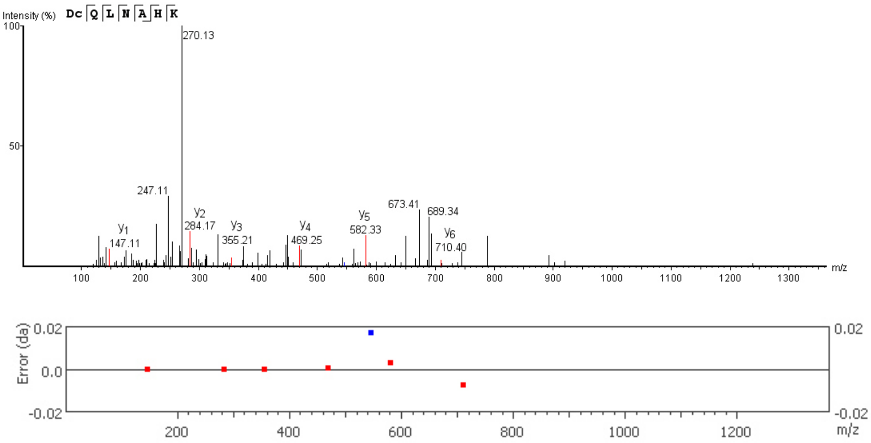

#	b	b (2+)	Seq	y	y (2+)	#
1	116.03	58.52	D			8
2	665.21	333.10	C(+446.17)	1259.57	630.28	7
3	793.27	397.13	Q	710.40	355.70	6
4	906.35	453.68	L	582.33	291.67	5
5	1020.40	510.70	N	469.25	235.13	4
6	1091.43	546.20	A	355.21	178.10	3
7	1228.49	614.75	H	284.17	142.59	2
8			K	147.11	74.06	1

[S7.11] Annexin A2

S(+42.01)TVHEILC(+446.17)K

m/z=759.3701 z=2

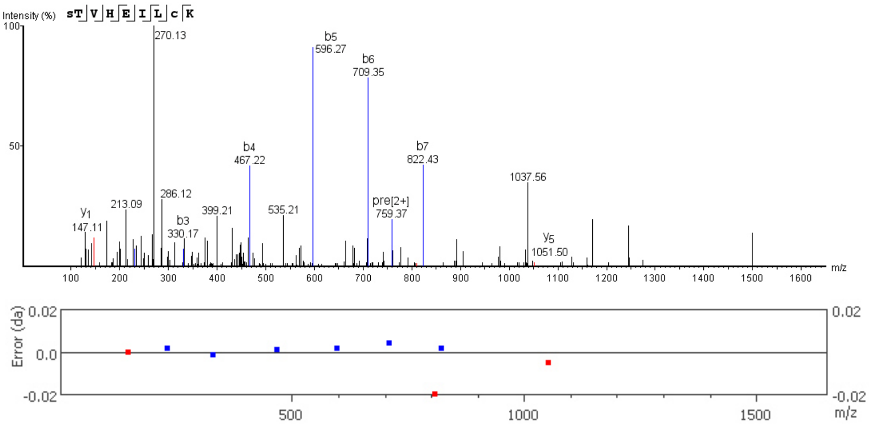

#	b	b (2+)	Seq	y	y (2+)	#
1	130.05	65.53	S(+42.01)			9
2	231.10	116.05	T	1388.67	694.84	8
3	330.17	165.58	V	1287.63	644.31	7
4	467.22	234.11	H	1188.56	594.78	6
5	596.27	298.63	E	1051.50	526.25	5
6	709.35	355.18	I	922.46	461.73	4
7	822.43	411.72	L	809.39	405.19	3
8	1371.61	686.31	C(+446.17)	696.29	348.64	2
9			K	147.11	74.06	1

[S7.12] Heterogeneous nuclear ribonucleoprotein K

GSDFDC(+446.17)ELR

m/z=744.31067 z=2

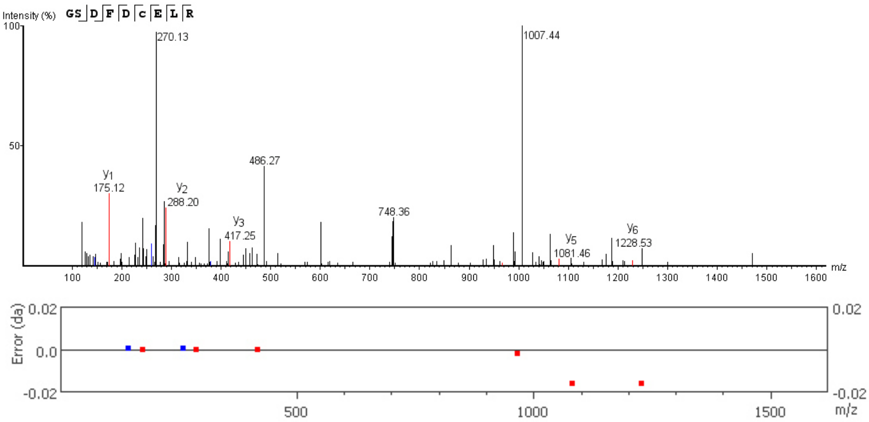

#	b	b (2+)	Seq	y	y (2+)	#
1	58.03	29.51	G			9
2	145.06	73.03	S	1430.57	715.79	8
3	260.09	130.54	D	1343.54	672.27	7
4	407.16	204.08	F	1228.53	614.76	6
5	522.18	261.59	D	1081.46	541.22	5
6	1071.36	536.18	C(+446.17)	966.42	483.71	4
7	1200.40	600.70	E	417.25	209.12	3
8	1313.49	657.24	L	288.20	144.60	2
9			R	175.12	88.06	1

Supplementary Figure S7

[S7.13] Lamin-B1

C(+446.17)QSLTEDLEFR

m/z=893.90204 z=2

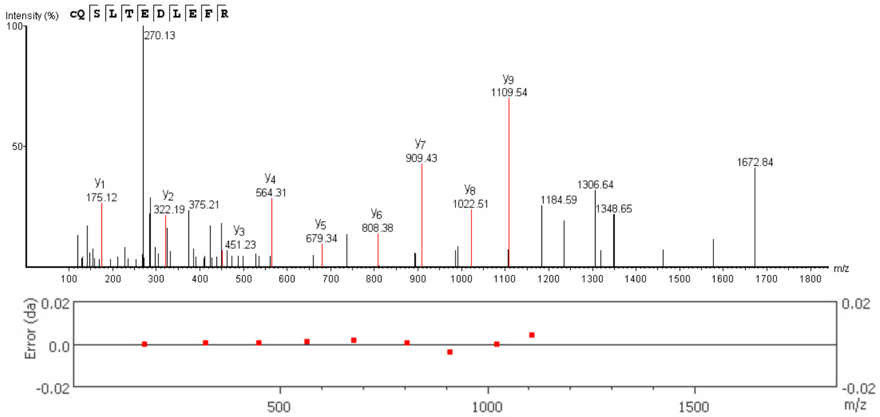

#	b	b (2+)	Seq	y	y (2+)	#
1	550.18	275.59	C(+446.17)			11
2	678.24	339.62	Q	1237.61	619.30	10
3	765.27	383.14	S	1109.54	555.27	9
4	878.36	439.68	L	1022.51	511.76	8
5	979.41	490.20	T	909.43	455.22	7
6	1108.45	554.72	E	808.38	404.69	6
7	1223.47	612.24	D	679.34	340.17	5
8	1336.56	668.78	L	564.31	282.66	4
9	1465.60	733.30	E	451.23	226.11	3
10	1612.67	806.83	F	322.19	161.59	2
11			R	175.12	88.06	1

[S7.14] 40S ribosomal protein S3

GC(+446.17)EVVVSGK

m/z=662.3185 z=2

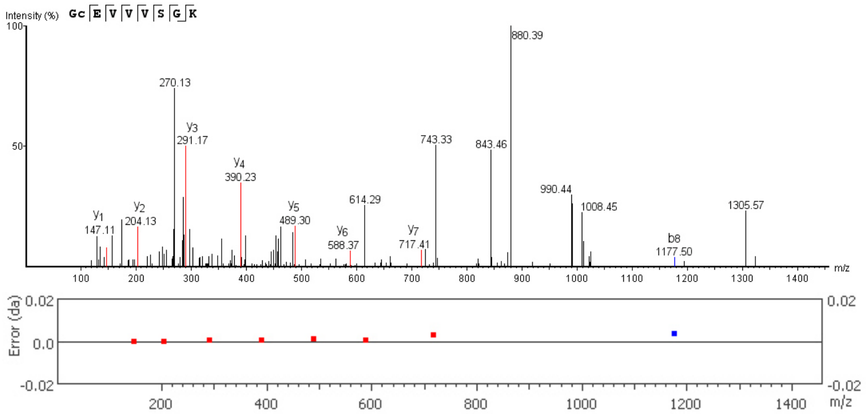

#	b	b (2+)	Seq	y	y (2+)	#
1	58.03	29.51	G			9
2	607.20	304.10	C(+446.17)	1266.59	633.79	8
3	736.25	368.62	E	717.41	359.21	7
4	835.32	418.16	V	588.37	294.69	6
5	934.38	467.69	V	489.30	245.15	5
6	1033.45	517.23	V	390.23	195.62	4
7	1120.48	560.74	S	291.17	146.08	3
8	1177.50	589.25	G	204.13	102.57	2
9			K	147.11	74.06	1

[S7.15] Voltage-dependent anion-selective channel protein 1

YQIDPDAC(+446.17)FSAK

m/z=902.39935 z=2

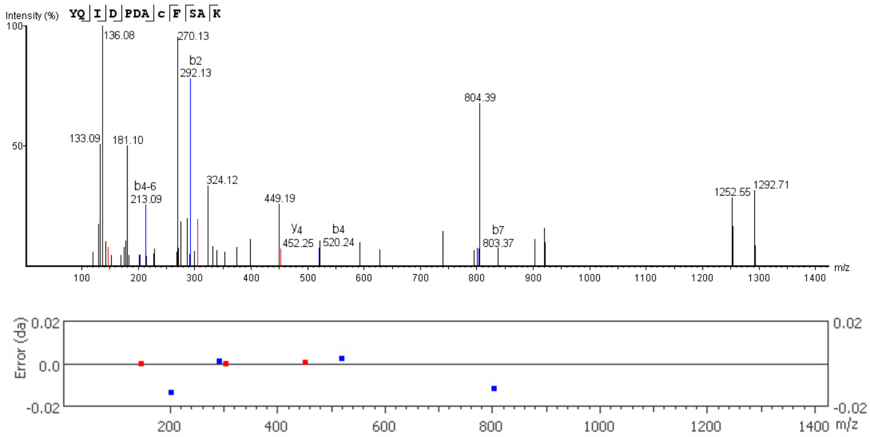

#	b	b (2+)	Seq	y	y (2+)	#
1	164.07	82.54	Y			12
2	292.13	146.56	Q	1640.71	820.86	11
3	405.21	203.12	I	1512.65	756.83	10
4	520.24	260.62	D	1399.57	700.28	9
5	617.29	309.15	P	1284.54	642.77	8
6	732.32	366.66	D	1187.49	594.24	7
7	803.37	402.18	A	1072.46	536.73	6
8	1352.53	676.77	C(+446.17)	1001.43	501.21	5
9	1499.60	750.30	F	452.25	226.63	4
10	1586.63	793.82	S	305.18	153.09	3
11	1657.67	829.34	A	218.15	109.57	2
12			K	147.11	74.06	1

Supplementary Figure S7

[S7.16] Glyceraldehyd-3-Phosphat Dehydrogenase

VPTANVSVVDLTC(+446.17)R

m/z=640.6566 z=3

#	b	b (2+)	Seq	y	y (2+)	#
1	100.08	50.54	V			14
2	197.13	99.06	P	1820.87	910.94	13
3	298.17	149.59	T	1723.82	862.41	12
4	369.21	185.11	A	1622.77	811.88	11
5	483.26	242.13	N	1551.73	776.37	10
6	582.33	291.66	V	1437.69	719.34	9
7	669.36	335.18	S	1338.62	669.81	8
8	768.43	384.71	V	1251.59	626.29	7
9	867.49	434.25	V	1152.52	576.76	6
10	982.52	491.76	D	1053.45	527.23	5
11	1095.60	548.30	L	938.43	469.71	4
12	1196.65	598.83	T	825.34	413.17	3
13	1745.83	873.41	C(+446.17)	724.29	362.65	2
14			R	175.12	88.06	1

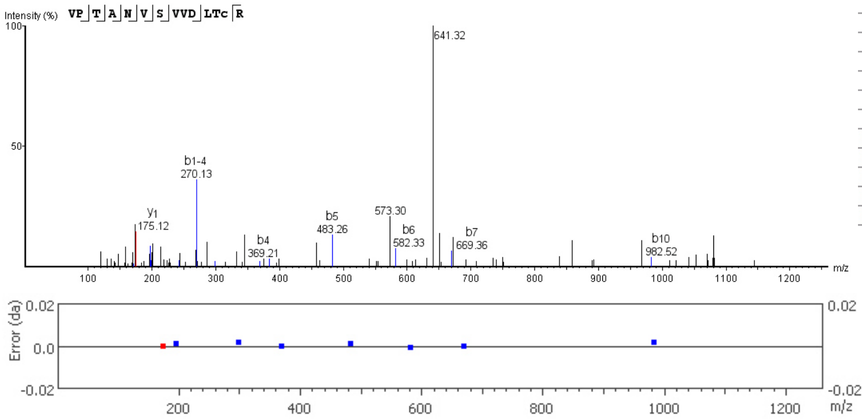

[S7.17] Heterogeneous nuclear ribonucleoproteins C1/C2

IVGC(+446.17)SVHK

m/z=430.2179 z=3

#	b	b (2+)	Seq	y	y (2+)	#
1	114.09	57.55	I			8
2	213.16	107.08	V	1175.54	588.27	7
3	270.18	135.59	G	1076.47	538.73	6
4	819.36	410.18	C(+446.17)	1019.45	510.22	5
5	906.39	453.69	S	470.27	235.64	4
6	1005.46	503.23	V	383.24	192.12	3
7	1142.52	571.76	H	284.17	142.59	2
8			K	147.11	74.06	1

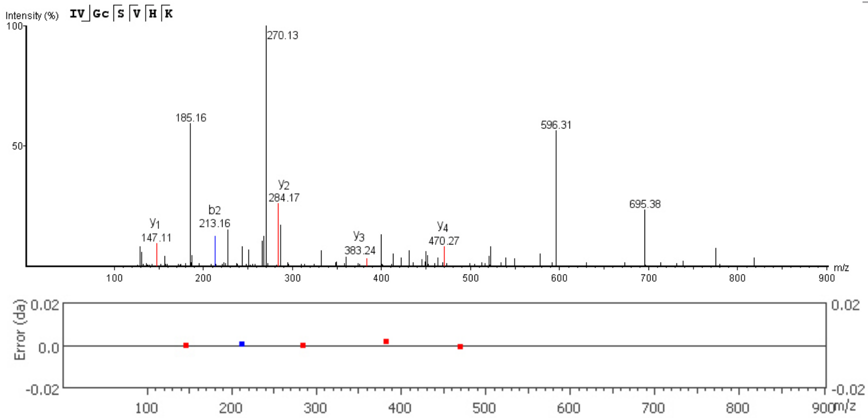

[S7.18] Peptidyl-prolyl cis-trans isomerase A

GSC(+446.17)FHR

m/z=384.83542 z=3

#	b	b (2+)	Seq	y	y (2+)	#
1	58.03	29.51	G			6
2	145.06	73.03	S	1095.45	548.23	5
3	694.24	347.62	C(+446.17)	1008.42	504.71	4
4	841.30	421.15	F	459.25	230.12	3
5	978.36	489.68	H	312.18	156.59	2
6			R	175.12	88.06	1

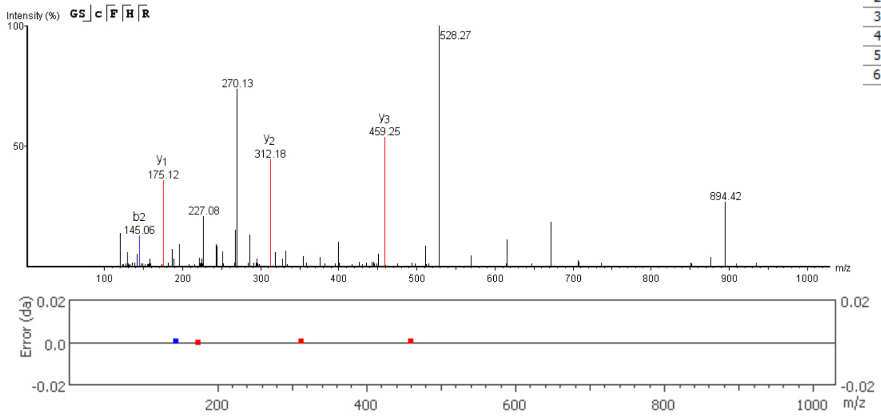

Supplementary Figure S7

[S7.19] PeptidylProlylCisTransIsomerase

IIPGFMC(+446.17)QGGDFTR

m/z=994.4574 z=2

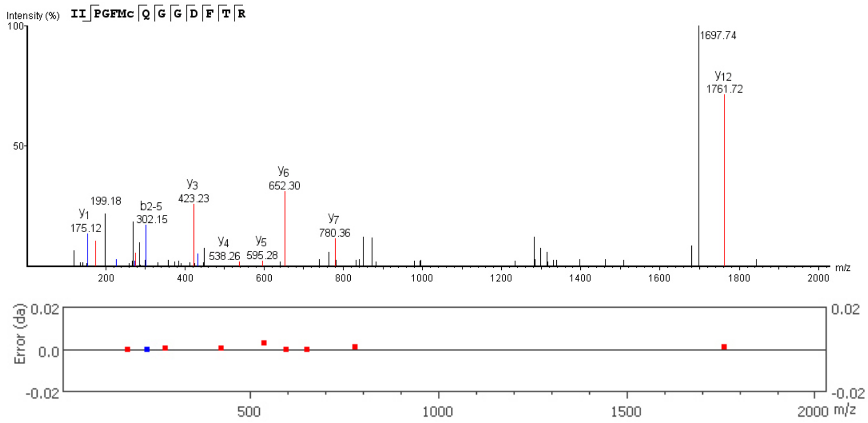

#	b	b (2+)	Seq	y	y (2+)	#
1	114.09	57.55	I			14
2	227.18	114.09	I	1874.81	937.90	13
3	324.23	162.61	P	1761.72	881.36	12
4	381.25	191.13	G	1664.67	832.83	11
5	528.32	264.66	F	1607.65	804.32	10
6	659.36	330.18	M	1460.58	730.79	9
7	1208.53	604.77	C(+446.17)	1329.54	665.27	8
8	1336.59	668.80	Q	780.36	390.68	7
9	1393.61	697.31	G	652.30	326.65	6
10	1450.64	725.82	G	595.28	298.14	5
11	1565.66	783.33	D	538.26	269.63	4
12	1712.73	856.87	F	423.23	212.12	3
13	1813.78	907.39	T	276.17	138.58	2
14			R	175.12	88.06	1

[S7.20] Colfin-1

AVLFC(+446.17)LSEDKK

m/z=566.95306 z=3

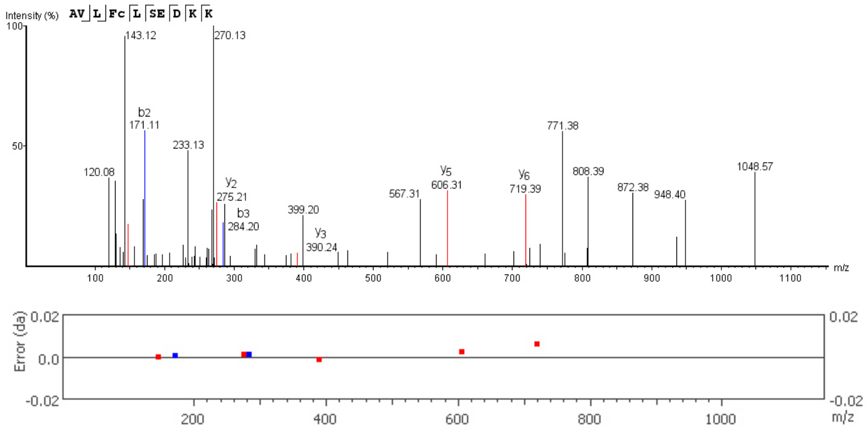

#	b	b (2+)	Seq	y	y (2+)	#
1	72.04	36.52	A			11
2	171.11	86.06	V	1627.79	814.39	10
3	284.20	142.60	L	1528.72	764.86	9
4	431.27	216.13	F	1415.64	708.32	8
5	980.44	490.72	C(+446.17)	1268.57	634.78	7
6	1093.52	547.26	L	719.39	360.20	6
7	1180.56	590.78	S	606.31	303.65	5
8	1309.60	655.30	E	519.28	260.14	4
9	1424.63	712.81	D	390.24	195.62	3
10	1552.72	776.86	K	275.21	138.10	2
11			K	147.11	74.06	1

[S7.21] Colfin-1

HELQANC(+446.17)YEEVK

m/z=954.9251 z=2

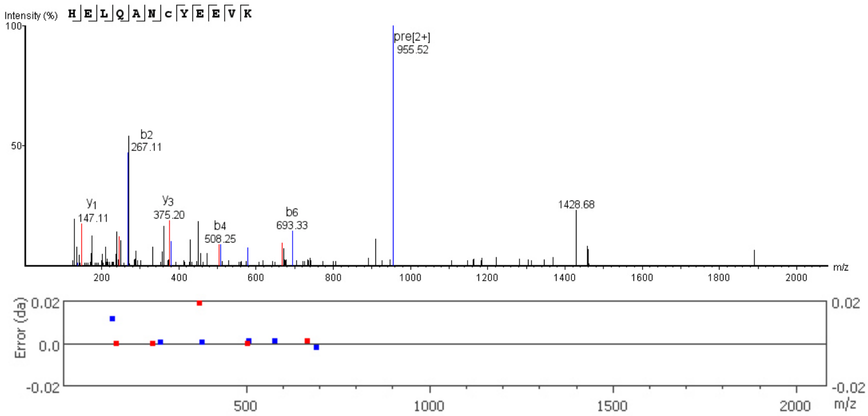

#	b	b (2+)	Seq	y	y (2+)	#
1	138.05	69.53	H			12
2	267.11	134.05	E	1771.77	886.38	11
3	380.19	190.60	L	1642.73	821.86	10
4	508.25	254.63	Q	1529.64	765.32	9
5	579.29	290.14	A	1401.58	701.29	8
6	693.33	347.17	N	1330.55	665.77	7
7	1242.51	621.75	C(+446.17)	1216.50	608.75	6
8	1405.57	703.29	Y	667.33	334.16	5
9	1534.61	767.81	E	504.27	252.63	4
10	1663.66	832.33	E	375.20	188.11	3
11	1762.72	881.86	V	246.18	123.59	2
12			K	147.11	74.06	1

Supplementary Figure S7

[S7.22] 40S ribosomal protein SA

YVDIAIPC(+446.17)NNK

m/z=848.4052 z=2

#	b	a	b (2+)	Seq	y	y (2+)	#
1	164.07	136.08	82.54	Y			11
2	263.14	235.14	132.07	V	1532.73	766.86	10
3	378.16	350.17	189.58	D	1433.66	717.33	9
4	491.25	463.26	246.13	I	1318.63	659.82	8
5	562.29	534.29	281.64	A	1205.55	603.27	7
6	675.37	647.38	338.19	I	1134.51	567.76	6
7	772.42	744.43	386.71	P	1021.43	511.21	5
8	1321.60	1293.60	661.30	C(+446.17)	924.37	462.69	4
9	1435.64	1407.65	718.32	N	375.20	188.10	3
10	1549.69	1521.69	775.34	N	261.15	131.08	2
11				K	147.11	74.06	1

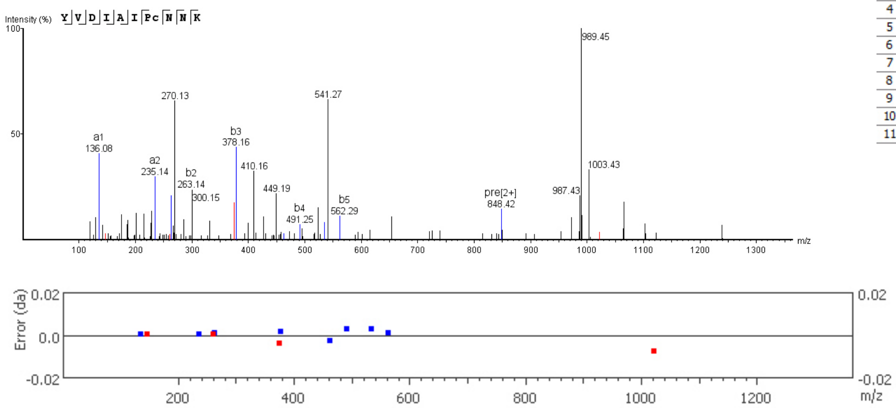

[S7.23] Heat shock protein HSP 90-alpha

FEELC(+446.17)SDLFR

m/z=852.884 z=2

#	b	b (2+)	Seq	y	y (2+)	#
1	148.08	74.54	F			10
2	277.12	139.06	E	1557.67	779.34	9
3	406.16	203.58	E	1428.63	714.82	8
4	519.25	260.12	L	1299.59	650.29	7
5	1068.42	534.71	C(+446.17)	1186.52	593.75	6
6	1155.45	578.23	S	637.33	319.17	5
7	1270.48	635.74	D	550.30	275.65	4
8	1383.56	692.28	L	435.27	218.14	3
9	1530.63	765.82	F	322.19	161.59	2
10			R	175.12	88.06	1

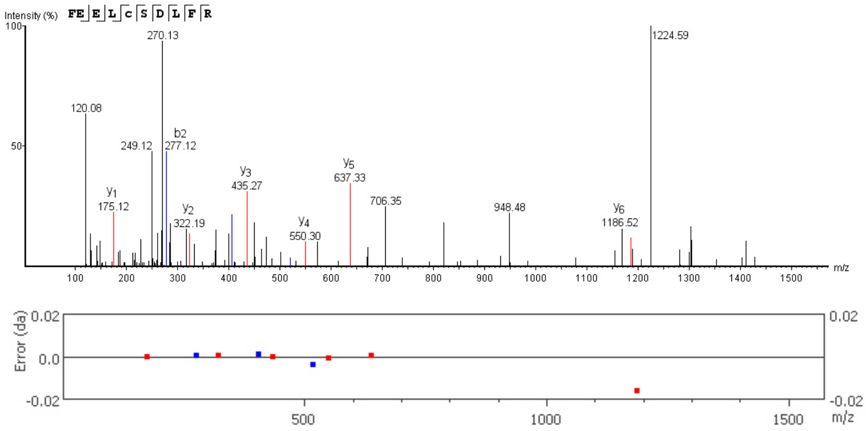

[S7.24] Nucleoside diphosphate kinase A

GDFC(+446.17)IQVGR

m/z=720.83356 z=2

#	b	b (2+)	Seq	y	y (2+)	#
1	58.03	29.51	G			9
2	173.06	87.03	D	1383.62	692.31	8
3	320.12	160.56	F	1268.59	634.80	7
4	869.30	435.15	C(+446.17)	1121.53	561.26	6
5	982.38	491.69	I	572.35	286.68	5
6	1110.44	555.72	Q	459.27	230.13	4
7	1209.51	605.26	V	331.21	166.10	3
8	1266.53	633.77	G	232.14	116.57	2
9			R	175.12	88.06	1

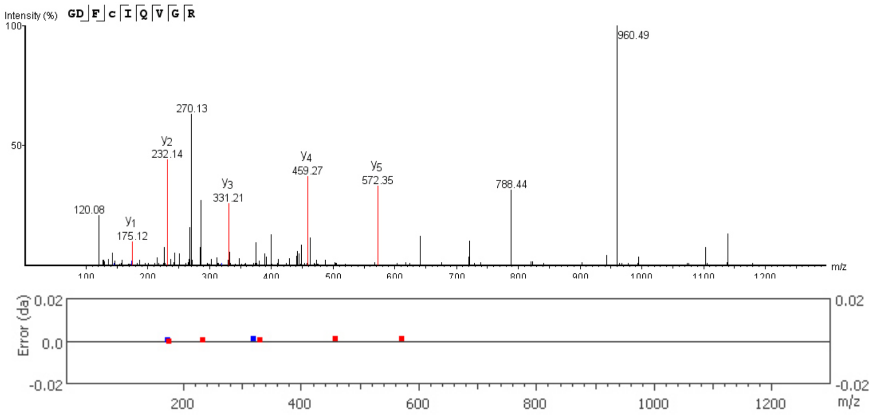

Supplementary Figure S7

[S7.25] 40S ribosomal protein S8

NC(+446.17)IVLIDSTPYR

m/z=920.4532 z=2

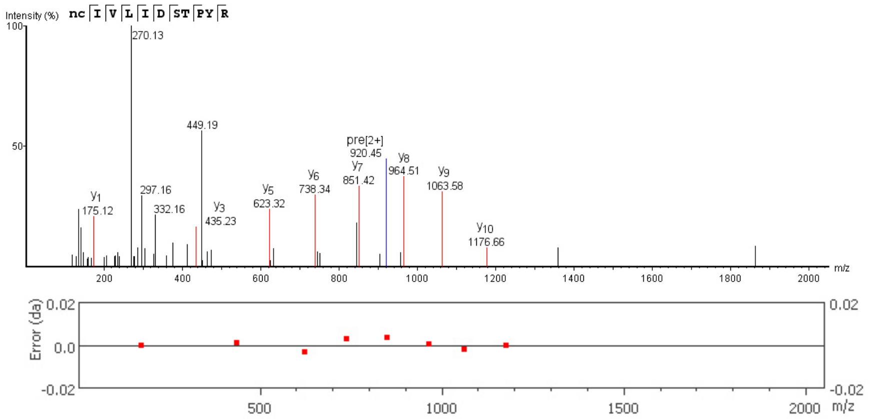

#	b	b (2+)	Seq	y	y (2+)	#
1	115.05	58.03	N			12
2	664.23	332.61	C(+446.17)	1725.84	863.42	11
3	777.31	389.15	I	1176.66	588.83	10
4	876.38	438.69	V	1063.58	532.29	9
5	989.46	495.23	L	964.51	482.75	8
6	1102.55	551.77	I	851.42	426.21	7
7	1217.57	609.29	D	738.34	369.67	6
8	1304.61	652.80	S	623.32	312.16	5
9	1405.65	703.33	T	536.28	268.64	4
10	1502.71	751.85	P	435.23	218.12	3
11	1665.77	833.38	Y	338.18	169.59	2
12			R	175.12	88.06	1

[S7.26] Profilin-1

C(+446.17)YEMASHLR

m/z=519.228 z=3

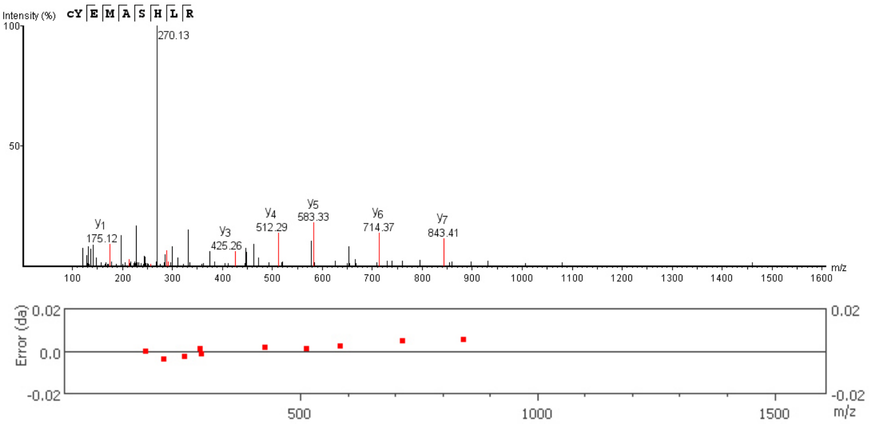

#	b	b (2+)	Seq	y	y (2+)	#
1	550.18	275.59	C(+446.17)			9
2	713.25	357.12	Y	1006.48	503.74	8
3	842.29	421.64	E	843.41	422.21	7
4	973.33	487.16	M	714.37	357.69	6
5	1044.37	522.68	A	583.33	292.17	5
6	1131.40	566.20	S	512.29	256.65	4
7	1268.46	634.73	H	425.26	213.13	3
8	1381.54	691.27	L	288.20	144.60	2
9			R	175.12	88.06	1

[S7.27] Phosphatidylethanolamine-binding protein 1

C(+446.17)DEPILSNR

m/z=746.8427 z=2

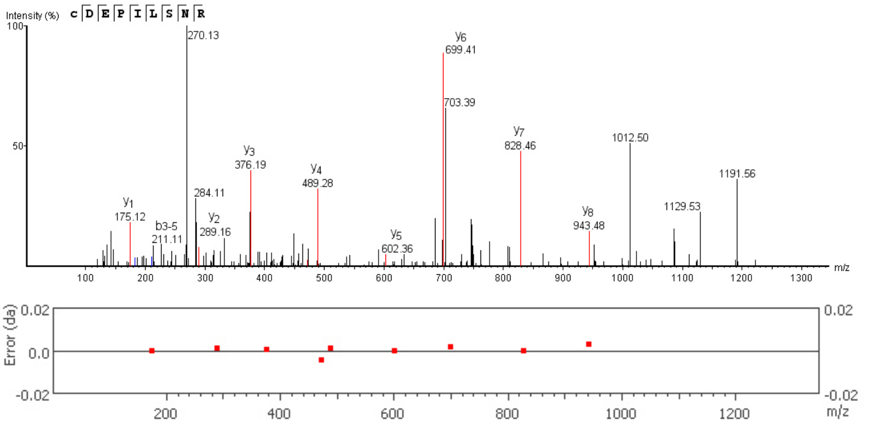

#	b	b (2+)	Seq	y	y (2+)	#
1	550.18	275.59	C(+446.17)			9
2	665.21	333.10	D	943.48	472.25	8
3	794.25	397.63	E	828.46	414.73	7
4	891.31	446.15	P	699.41	350.21	6
5	1004.39	502.69	I	602.36	301.68	5
6	1117.47	559.24	L	489.28	245.14	4
7	1204.51	602.75	S	376.19	188.60	3
8	1318.55	659.77	N	289.16	145.08	2
9			R	175.12	88.06	1

Supplementary Figure S7

[S7.28] Voltage-dependent anion-selective channel protein 2

WC(+446.17)EYGLTFTEK

m/z=911.90094 z=2

#	b	b (2+)	Seq	y	y (2+)	#
1	187.09	94.04	W			11
2	736.26	368.63	C(+446.17)	1636.71	818.85	10
3	865.30	433.15	E	1087.54	544.27	9
4	1028.37	514.68	Y	958.48	479.74	8
5	1085.39	543.19	G	795.42	398.21	7
6	1198.47	599.74	L	738.40	369.70	6
7	1299.52	650.26	T	625.32	313.16	5
8	1446.59	723.79	F	524.27	262.64	4
9	1547.64	774.32	T	377.20	189.10	3
10	1676.68	838.84	E	276.16	138.58	2
11			K	147.11	74.06	1

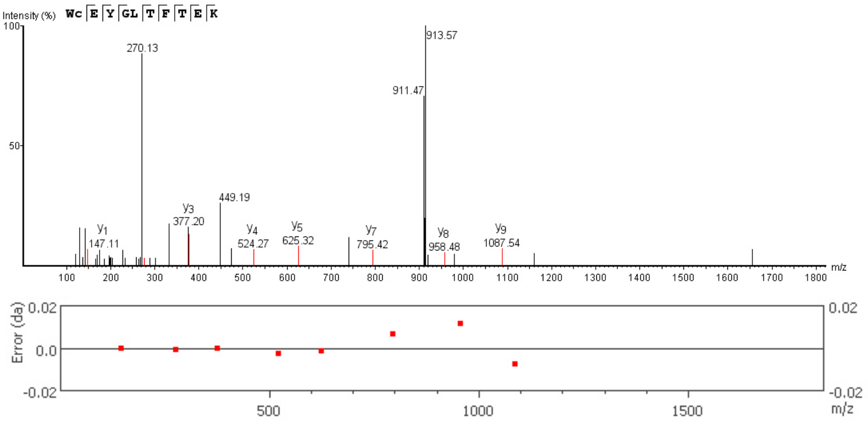

[S7.29] 40S ribosomal protein S16

TATAVAHC(+446.17)K

m/z=674.3229 z=2

#	b	b (2+)	Seq	y	y (2+)	#
1	102.06	51.53	T			9
2	173.09	87.05	A	1246.57	623.79	8
3	274.14	137.57	T	1175.54	588.27	7
4	345.18	173.09	A	1074.49	537.74	6
5	444.25	222.62	V	1003.45	502.23	5
6	515.28	258.14	A	904.40	452.69	4
7	652.34	326.67	H	833.36	417.17	3
8	1201.52	601.26	C(+446.17)	696.29	348.64	2
9			K	147.11	74.06	1

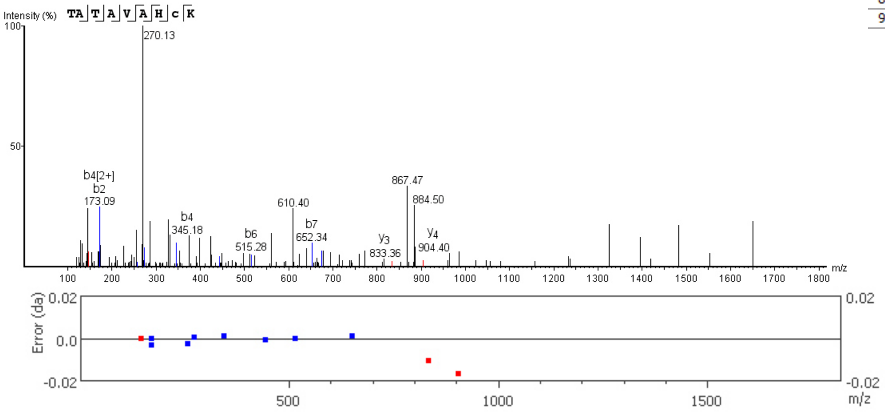

[S7.30] Spermine Synthase

LYC(+446.17)PVEFSK

m/z=766.3578 z=2

#	b	a	b (2+)	Seq	y	y (2+)	#
1	114.09	86.10	57.55	L			9
2	277.16	249.16	139.08	Y	1418.62	709.81	8
3	826.33	798.34	413.67	C(+446.17)	1255.55	628.28	7
4	923.38	895.39	462.19	P	706.38	353.69	6
5	1022.45	994.46	511.73	V	609.32	305.16	5
6	1151.49	1123.50	576.25	E	510.25	255.63	4
7	1298.56	1270.57	649.78	F	381.21	191.11	3
8	1385.59	1357.60	693.30	S	234.14	117.57	2
9				K	147.11	74.06	1

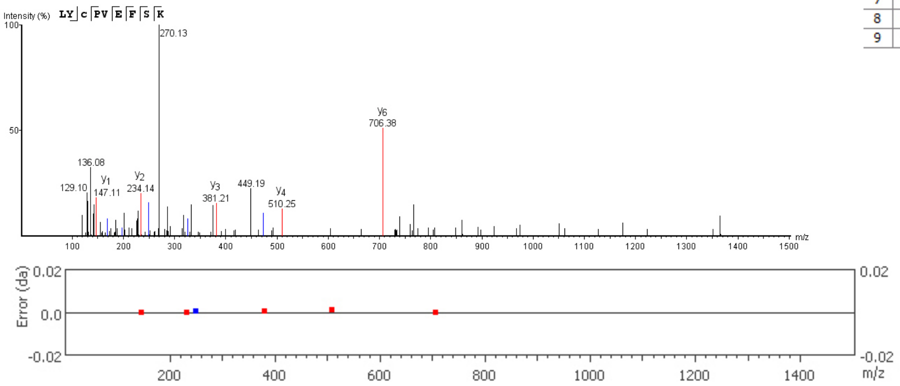

Supplementary Figure S7

[S7.31] GTP-binding nuclear protein Ran

VC(+446.17)ENIPIVLC(+414.19)GNK

m/z=1131.553 z=2

#	b	b (2+)	Seq	y	y (2+)	#
1	100.08	50.54	V			13
2	649.25	325.13	C(+446.17)	2163.01	1082.01	12
3	778.29	389.65	E	1613.84	807.42	11
4	892.34	446.67	N	1484.80	742.90	10
5	1005.42	503.21	I	1370.75	685.88	9
6	1102.47	551.74	P	1257.67	629.33	8
7	1215.56	608.28	I	1160.62	580.81	7
8	1314.63	657.81	V	1047.52	524.27	6
9	1427.71	714.36	L	948.47	474.73	5
10	1944.91	972.96	C(+414.19)	835.38	418.19	4
11	2001.93	1001.47	G	318.18	159.59	3
12	2115.98	1058.49	N	261.16	131.08	2
13			K	147.11	74.06	1

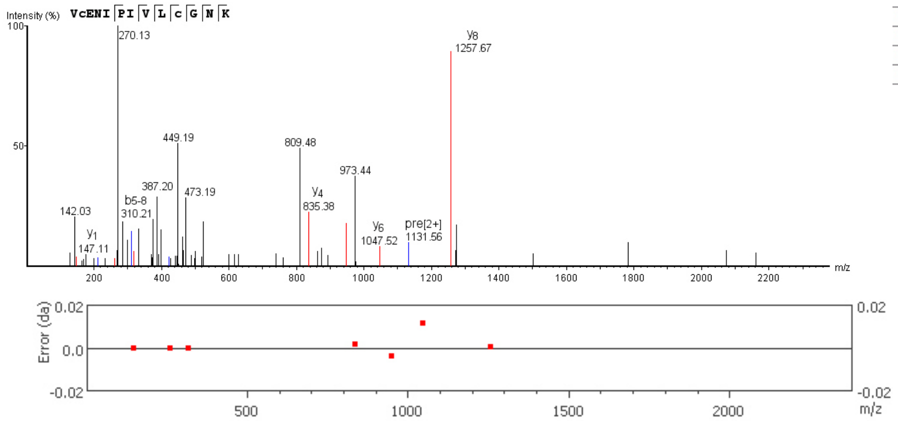

[S7.32] Small nuclear ribonucleoprotein Sm D2

NNTQVLINC(+446.17)R

m/z=810.89215 z=2

#	b	b (2+)	Seq	y	y (2+)	#
1	115.05	58.03	N			10
2	229.09	115.05	N	1506.72	753.86	9
3	330.14	165.57	T	1392.68	696.84	8
4	458.20	229.60	Q	1291.63	646.32	7
5	557.27	279.13	V	1163.57	582.29	6
6	670.35	335.68	L	1064.51	532.75	5
7	783.44	392.22	I	951.44	476.21	4
8	897.48	449.24	N	838.35	419.67	3
9	1446.65	723.83	C(+446.17)	724.29	362.65	2
10			R	175.12	88.06	1

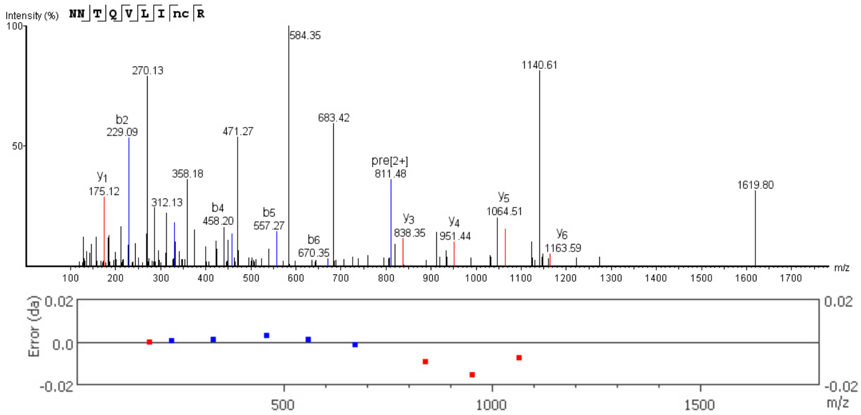

[S7.33] 60S ribosomal protein L18

GC(+446.17)GTVLLSGPR

m/z=753.37744 z=2

#	b	b (2+)	Seq	y	y (2+)	#
1	58.03	29.51	G			11
2	607.20	304.10	C(+446.17)	1448.71	724.85	10
3	664.23	332.61	G	899.53	450.27	9
4	765.27	383.14	T	842.50	421.75	8
5	864.34	432.67	V	741.46	371.23	7
6	977.43	489.21	L	642.39	321.70	6
7	1090.51	545.75	L	529.31	265.15	5
8	1177.54	589.27	S	416.22	208.61	4
9	1234.56	617.78	G	329.19	165.10	3
10	1331.62	666.31	P	272.17	136.59	2
11			R	175.12	88.06	1

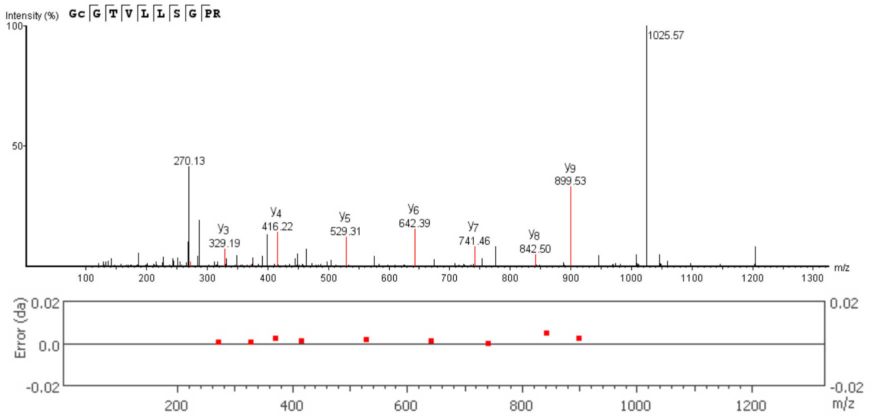

Supplementary Figure S7

[S7.34] 60S ribosomal protein L13a

C(+446.17)EGINISGNFYR

m/z=909.9098 z=2

#	b	b (2+)	Seq	y	y (2+)	#
1	550.18	275.59	C(+446.17)			12
2	679.23	340.11	E	1269.62	635.31	11
3	736.25	368.62	G	1140.57	570.79	10
4	849.33	425.17	I	1083.56	542.28	9
5	963.37	482.19	N	970.48	485.74	8
6	1076.46	538.73	I	856.43	428.72	7
7	1163.49	582.24	S	743.34	372.17	6
8	1220.51	610.76	G	656.31	328.66	5
9	1334.55	667.78	N	599.29	300.15	4
10	1481.62	741.31	F	485.25	243.13	3
11	1644.69	822.84	Y	338.18	169.59	2
12			R	175.12	88.06	1

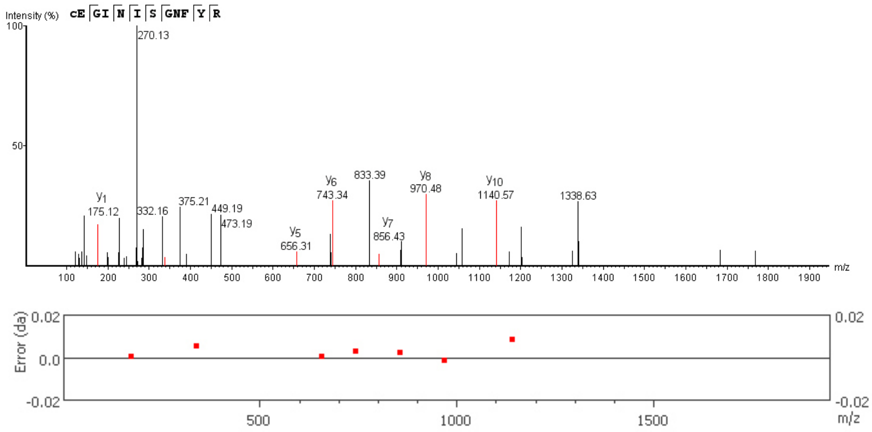

[S7.35] Cellular nucleic acid-binding protein

C(+446.17)GESGHLAR

m/z=459.20724 z=3

#	b	b (2+)	Seq	y	y (2+)	#
1	550.18	275.59	C(+446.17)			9
2	607.20	304.10	G	826.41	413.71	8
3	736.25	368.62	E	769.39	385.20	7
4	823.28	412.14	S	640.35	320.68	6
5	880.30	440.65	G	553.32	277.16	5
6	1017.36	509.18	H	496.30	248.65	4
7	1130.44	565.72	L	359.24	180.12	3
8	1201.48	601.24	A	246.16	123.58	2
9			R	175.12	88.06	1

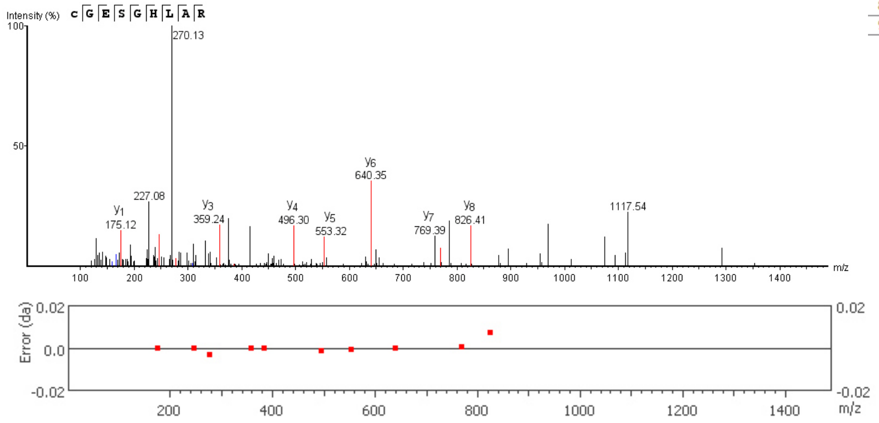

[S7.36] 60S ribosomal Protein L12

C(+446.17)TGGEVGATSALAPK

m/z=904.43085 z=2

#	b	b (2+)	Seq	y	y (2+)	#
1	550.18	275.59	C(+446.17)			15
2	651.23	326.12	T	1258.66	629.83	14
3	708.25	354.63	G	1157.61	579.31	13
4	765.27	383.14	G	1100.59	550.80	12
5	894.32	447.66	E	1043.57	522.29	11
6	993.38	497.19	V	914.52	457.77	10
7	1050.41	525.70	G	815.46	408.23	9
8	1121.44	561.22	A	758.43	379.72	8
9	1222.49	611.75	T	687.40	344.20	7
10	1309.52	655.26	S	586.36	293.68	6
11	1380.56	690.78	A	499.32	250.16	5
12	1493.64	747.32	L	428.29	214.64	4
13	1564.68	782.84	A	315.20	158.10	3
14	1661.73	831.37	P	244.16	122.58	2
15			K	147.11	74.06	1

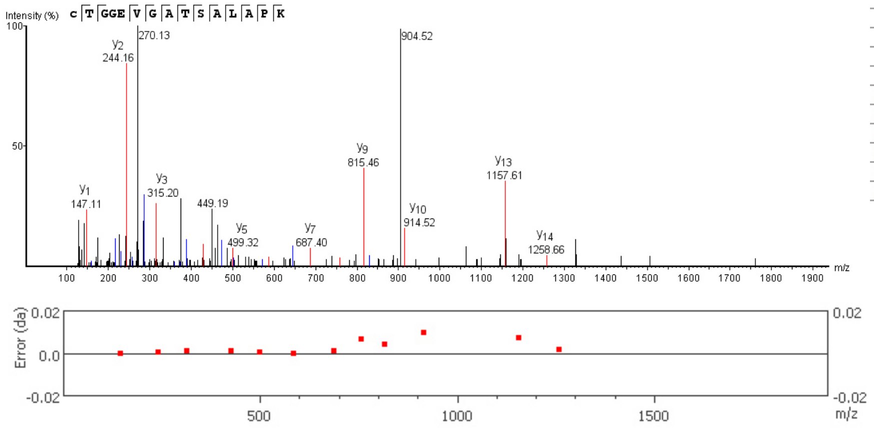

Supplementary Figure S7

[S7.37] Alpha-2-HS-glycoprotein

C(+446.17)DSSPDSAEDVR

m/z=863.83984 z=2

#	b	b (2+)	Seq	y	y (2+)	#
1	550.18	275.59	C(+446.17)			12
2	665.21	333.10	D	1177.49	589.25	11
3	752.24	376.62	S	1062.47	531.73	10
4	839.27	420.14	S	975.44	488.22	9
5	936.33	468.66	P	888.40	444.70	8
6	1051.35	526.18	D	791.35	396.18	7
7	1138.39	569.69	S	676.32	338.66	6
8	1209.42	605.21	A	589.29	295.15	5
9	1338.47	669.73	E	518.25	259.63	4
10	1453.49	727.25	D	389.21	195.11	3
11	1552.56	776.78	V	274.19	137.59	2
12			R	175.12	88.06	1

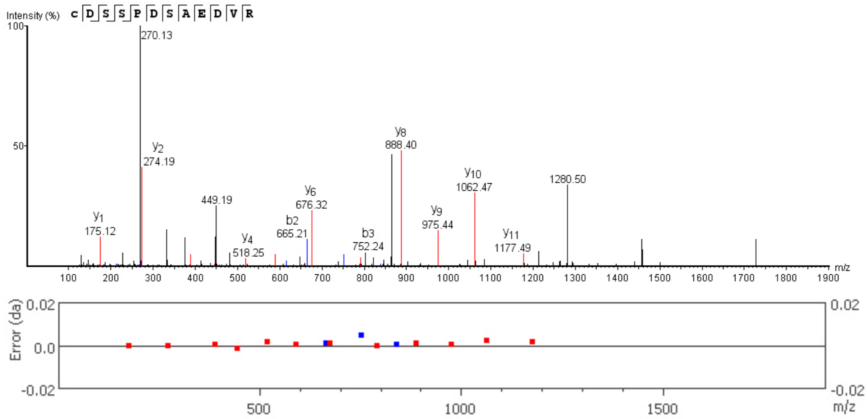

[S7.38] Macrophage migration inhibitory factor

LLC(+446.17)GLLAER

m/z=717.3777 z=2

#	b	b (2+)	Seq	y	y (2+)	#
1	114.09	57.55	L			9
2	227.17	114.09	L	1320.65	660.82	8
3	776.35	388.68	C(+446.17)	1207.58	604.28	7
4	833.37	417.19	G	658.39	329.69	6
5	946.46	473.73	L	601.37	301.18	5
6	1059.54	530.27	L	488.28	244.64	4
7	1130.58	565.79	A	375.20	188.10	3
8	1259.62	630.31	E	304.16	152.58	2
9			R	175.12	88.06	1

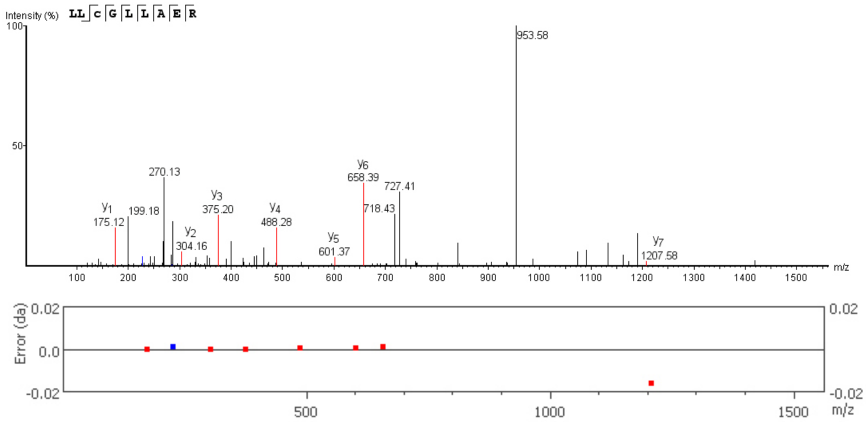

[S7.39] DUTP pyrophosphatase, isoform CRA_c

IAQLIC(+446.17)ER

m/z=696.35547 z=2

#	b	a	b (2+)	Seq	y	y (2+)	#
1	114.09	86.10	57.55	I			8
2	185.13	157.13	93.06	A	1278.60	639.80	7
3	313.19	285.19	157.09	Q	1207.56	604.28	6
4	426.27	398.28	213.64	L	1079.52	540.26	5
5	539.36	511.36	270.18	I	966.42	483.71	4
6	1088.53	1060.54	544.77	C(+446.17)	853.35	427.17	3
7	1217.57	1189.57	609.29	E	304.16	152.58	2
8				R	175.12	88.06	1

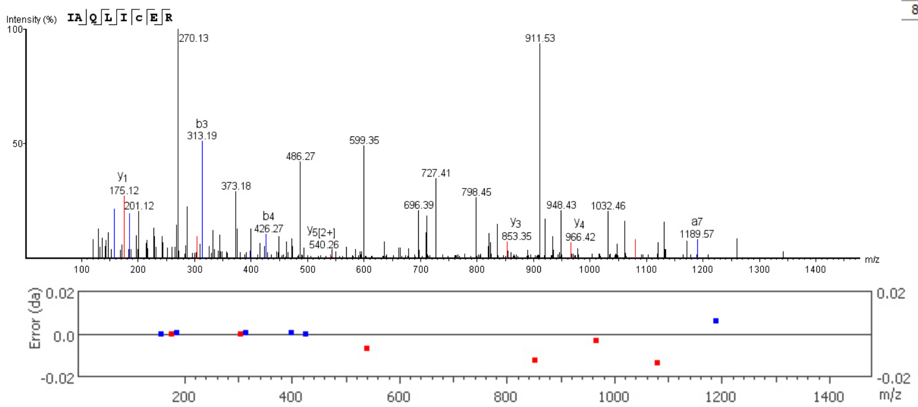

Supplementary Figure S7

[S7.40] Heat shock 70 kDa protein 1A/1B

FEELC(+446.17)SDLFR

m/z=852.8824 z=2

#	b	b (2+)	Seq	y	y (2+)	#
1	148.08	74.54	F			10
2	277.12	139.06	E	1557.67	779.34	9
3	406.16	203.58	E	1428.63	714.82	8
4	519.25	260.12	L	1299.59	650.29	7
5	1068.42	534.71	C(+446.17)	1186.52	593.75	6
6	1155.45	578.23	S	637.33	319.17	5
7	1270.48	635.74	D	550.30	275.65	4
8	1383.56	692.28	L	435.27	218.14	3
9	1530.63	765.82	F	322.19	161.59	2
10			R	175.12	88.06	1

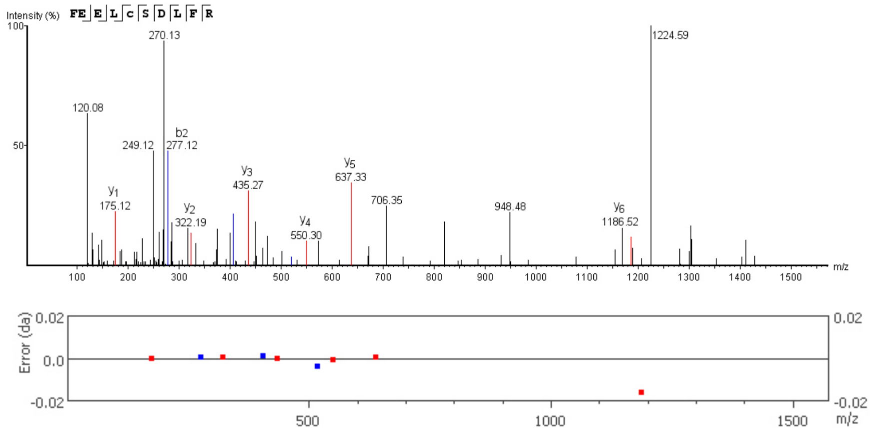

[S7.41] Heterogeneous nuclear ribonucleoprotein M

GC(+446.17)AVVEFK

m/z=649.8092 z=2

#	b	b (2+)	Seq	y	y (2+)	#
1	58.03	29.51	G			8
2	607.20	304.10	C(+446.17)	1241.57	621.29	7
3	678.24	339.62	A	692.40	346.70	6
4	777.31	389.15	V	621.36	311.18	5
5	876.38	438.69	V	522.29	261.65	4
6	1005.42	503.21	E	423.22	212.11	3
7	1152.49	576.74	F	294.18	147.59	2
8			K	147.11	74.06	1

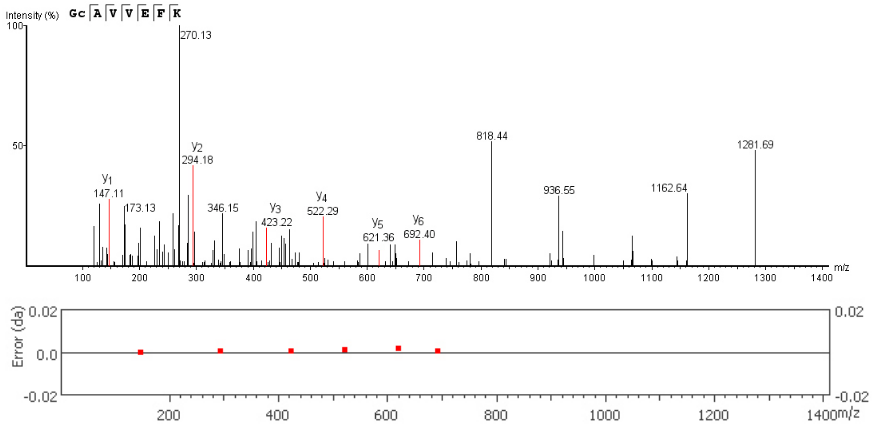

[S7.42] Plastin-3

AVGDGIVLC(+446.17)K

m/z=710.8606 z=2

#	b	b (2+)	Seq	y	y (2+)	#
1	72.04	36.52	A			10
2	171.11	86.06	V	1349.66	675.33	9
3	228.13	114.57	G	1250.59	625.80	8
4	343.16	172.08	D	1193.59	597.29	7
5	400.18	200.59	G	1078.55	539.77	6
6	513.26	257.13	I	1021.52	511.26	5
7	612.33	306.67	V	908.44	454.72	4
8	725.42	363.21	L	809.37	405.19	3
9	1274.59	637.80	C(+446.17)	696.29	348.64	2
10			K	147.11	74.06	1

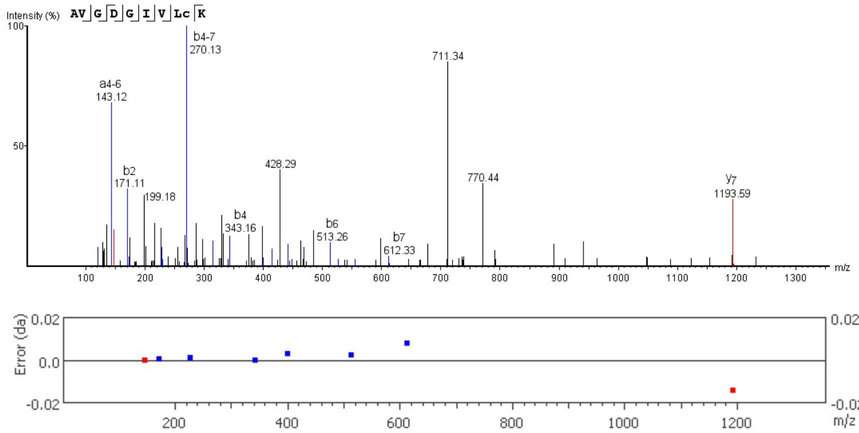

Supplementary Figure S7

[S7.43] Poly [ADP-ribose] polymerase 1

VC(+446.17)STNDLK

m/z=663.3062 z=2

#	b	b (2+)	Seq	y	y (2+)	#
1	100.08	50.54	V			8
2	649.25	325.13	C(+446.17)	1226.52	613.76	7
3	736.28	368.64	S	677.35	339.17	6
4	837.33	419.17	T	590.31	295.66	5
5	951.37	476.19	N	489.27	245.13	4
6	1066.40	533.70	D	375.20	188.11	3
7	1179.48	590.24	L	260.20	130.60	2
8			K	147.11	74.06	1

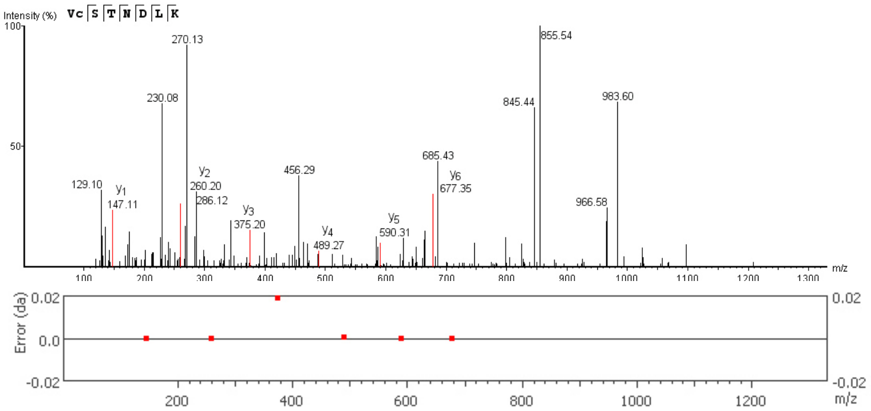

[S7.44] Pyruvate kinase PKM

NTGIIC(+446.17)TIGPASR

m/z=874.9383 z=2

#	b	b (2+)	Seq	y	y (2+)	#
1	115.05	58.03	N			13
2	216.10	108.55	T	1634.81	817.90	12
3	273.12	137.06	G	1533.76	767.38	11
4	386.20	193.60	I	1476.74	738.87	10
5	499.29	250.14	I	1363.65	682.33	9
6	1048.46	524.73	C(+446.17)	1250.57	625.78	8
7	1149.51	575.26	T	701.39	351.20	7
8	1262.59	631.80	I	600.34	300.67	6
9	1319.62	660.31	G	487.26	244.13	5
10	1416.67	708.83	P	430.24	215.62	4
11	1487.71	744.35	A	333.19	167.09	3
12	1574.74	787.87	S	262.15	131.58	2
13			R	175.12	88.06	1

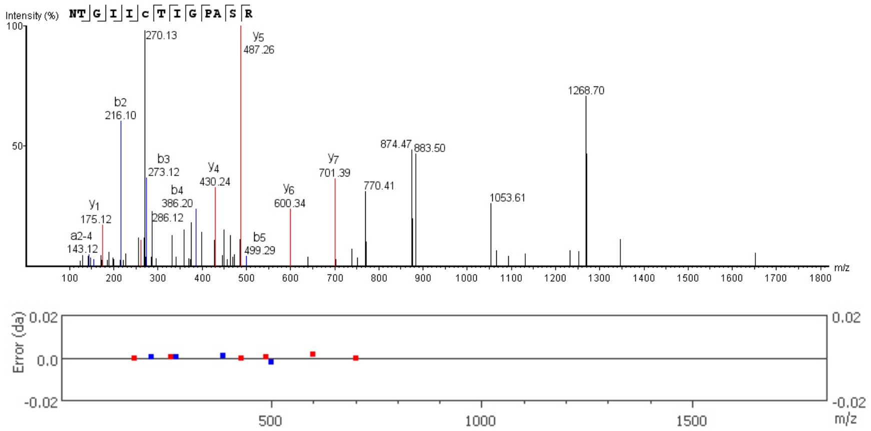

[S7.45] Pyruvate Kinase PKM

C(+446.17)C(+414.19)SGAIIVLTK

m/z=984.4862 z=2

#	b	b (2+)	Seq	y	y (2+)	#
1	550.18	275.59	C(+446.17)			11
2	1067.39	534.19	C(+414.19)	1418.77	709.89	10
3	1154.42	577.71	S	901.57	451.29	9
4	1211.44	606.22	G	814.54	407.77	8
5	1282.48	641.74	A	757.52	379.26	7
6	1395.56	698.28	I	686.48	343.74	6
7	1508.64	754.82	I	573.40	287.20	5
8	1607.71	804.36	V	460.31	230.66	4
9	1720.80	860.90	L	361.24	181.12	3
10	1821.84	911.42	T	248.16	124.58	2
11			K	147.11	74.06	1

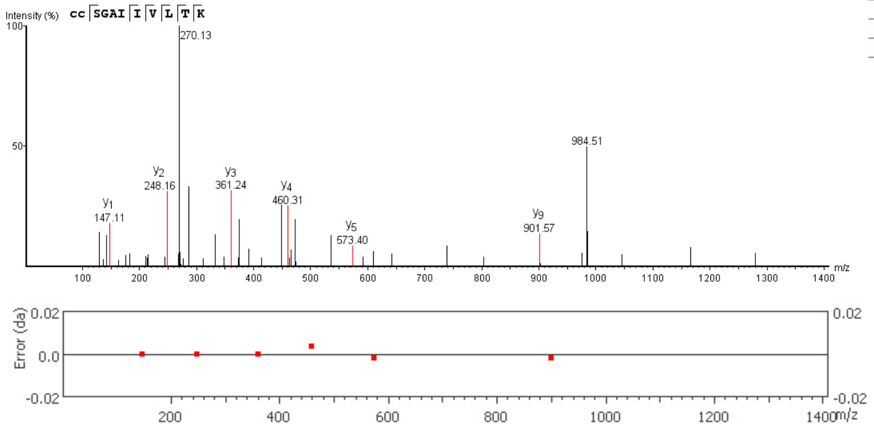

Supplementary Figure S7

[S7.46] Ubiquitin carboxyl-terminal hydrolase isozyme L1

FSAVALC(+446.17)K

m/z=642.81976 z=2

#	b	a	b (2+)	Seq	y	y (2+)	#
1	148.08	120.08	74.54	F			8
2	235.10	207.11	118.05	S	1137.55	569.27	7
3	306.14	278.15	153.57	A	1050.51	525.76	6
4	405.21	377.22	203.11	V	979.48	490.24	5
5	476.25	448.26	238.63	A	880.41	440.70	4
6	589.33	561.34	295.17	L	809.37	405.19	3
7	1138.51	1110.51	569.75	C(+446.17)	696.30	348.64	2
8				K	147.11	74.06	1

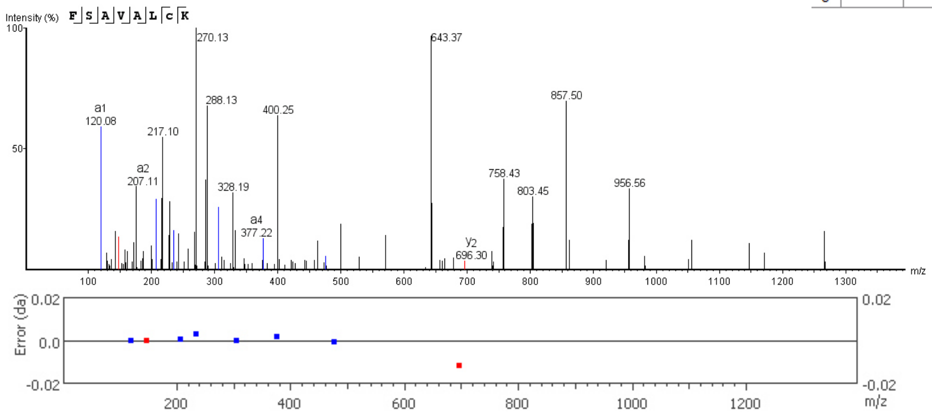

[S7.47] Transitional endoplasmic reticulum ATPase

LGDVISIQPC(+446.17)PDVK

m/z=965.4863 z=2

#	b	b (2+)	Seq	y	y (2+)	#
1	114.09	57.55	L			14
2	171.11	86.06	G	1816.86	908.93	13
3	286.14	143.57	D	1759.84	880.42	12
4	385.21	193.10	V	1644.82	822.91	11
5	498.29	249.65	I	1545.75	773.37	10
6	585.32	293.16	S	1432.66	716.83	9
7	698.41	349.70	I	1345.63	673.32	8
8	826.47	413.73	Q	1232.55	616.77	7
9	923.52	462.26	P	1104.49	552.76	6
10	1472.70	736.85	C(+446.17)	1007.44	504.22	5
11	1569.75	785.37	P	458.26	229.63	4
12	1684.77	842.89	D	361.21	181.10	3
13	1783.84	892.42	V	246.18	123.59	2
14			K	147.11	74.06	1

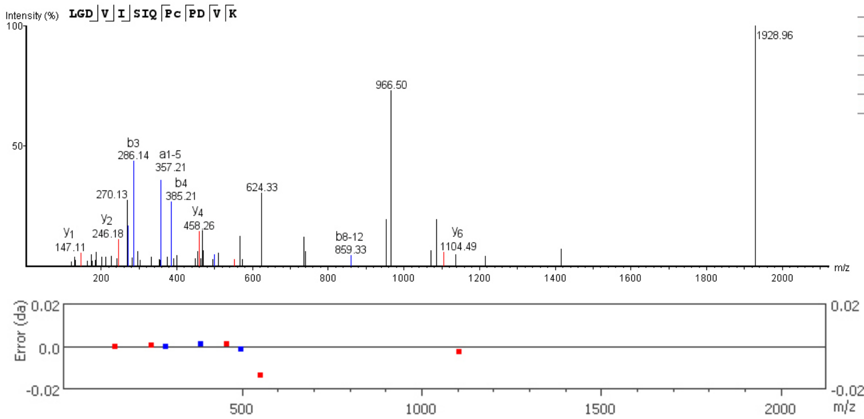

[S7.48] 60S ribosomal Protein L11

IAVHC(+446.17)TVR

m/z=672.8422 z=2

#	b	b (2+)	Seq	y	y (2+)	#
1	114.09	57.55	I			8
2	185.13	93.06	A	1231.57	616.30	7
3	284.20	142.60	V	1160.54	580.78	6
4	421.26	211.11	H	1061.48	531.23	5
5	970.43	485.72	C(+446.17)	924.43	462.71	4
6	1071.48	536.24	T	375.24	188.12	3
7	1170.55	585.77	V	274.19	137.59	2
8			R	175.12	88.06	1

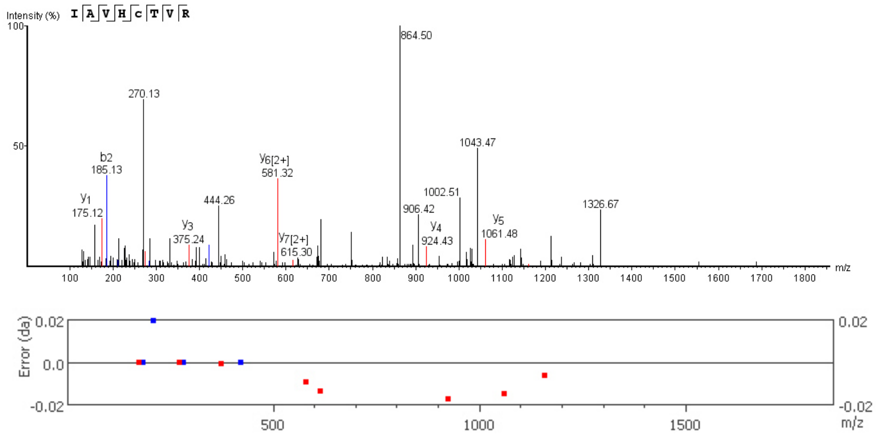

Supplementary Figure S7

[S7.49] DNA replication licensing factor MCM3

C(+446.17)SVLAAANPVYGR

m/z=883.93005 z=2

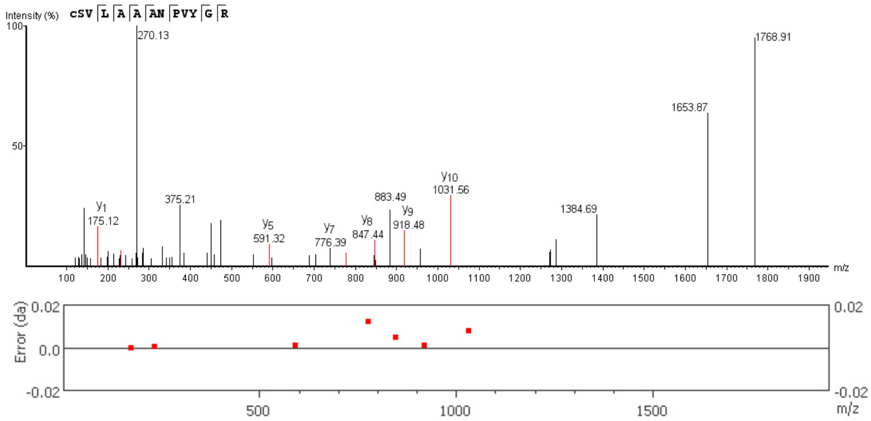

#	b	b (2+)	Seq	y	y (2+)	#
1	550.18	275.59	C(+446.17)			13
2	637.21	319.11	S	1217.66	609.33	12
3	736.28	368.64	V	1130.63	565.82	11
4	849.37	425.18	L	1031.56	516.28	10
5	920.40	460.70	A	918.48	459.74	9
6	991.44	496.22	A	847.44	424.22	8
7	1062.48	531.74	A	776.39	388.70	7
8	1176.52	588.76	N	705.37	353.18	6
9	1273.57	637.29	P	591.32	296.16	5
10	1372.64	686.82	V	494.27	247.64	4
11	1535.71	768.35	Y	395.20	198.10	3
12	1592.73	796.86	G	232.14	116.57	2
13			R	175.12	88.06	1

[S7.50] Malate dehydrogenase, mitochondrial

EGVVEC(+446.17)SFVK

m/z=771.8625 z=2

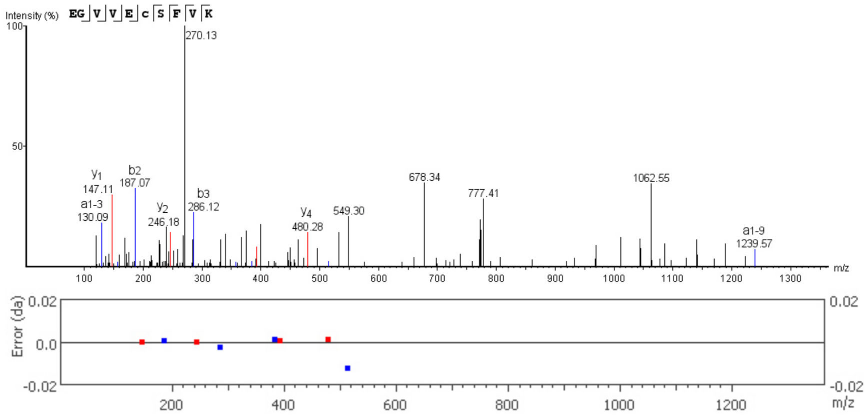

#	b	b (2+)	Seq	y	y (2+)	#
1	130.05	65.53	E			10
2	187.07	94.04	G	1413.66	707.33	9
3	286.14	143.57	V	1356.64	678.82	8
4	385.21	193.10	V	1257.57	629.28	7
5	514.26	257.63	E	1158.50	579.75	6
6	1063.43	532.21	C(+446.17)	1029.46	515.23	5
7	1150.46	575.73	S	480.28	240.64	4
8	1297.53	649.26	F	393.25	197.12	3
9	1396.60	698.80	V	246.18	123.59	2
10			K	147.11	74.06	1

[S7.51] Nuclear autoantigenic sperm protein

SVSGTDVQEEC(+446.17)R

m/z=878.3794 z=2

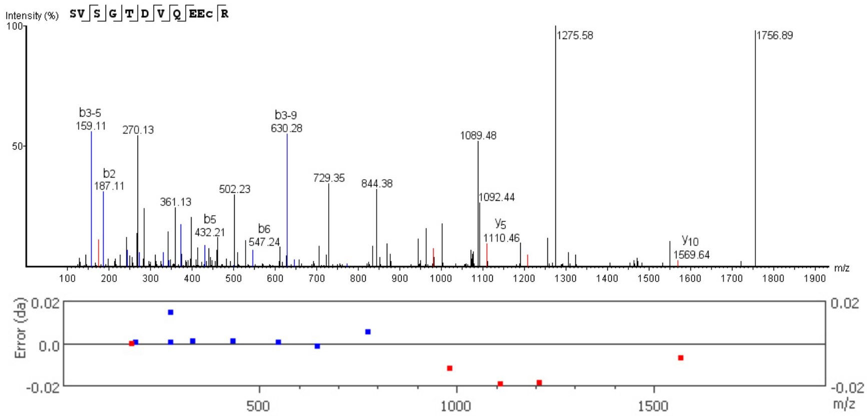

#	b	b (2+)	Seq	y	y (2+)	#
1	88.04	44.52	S			12
2	187.11	94.05	V	1668.70	834.85	11
3	274.14	137.57	S	1569.64	785.32	10
4	331.16	166.08	G	1482.60	741.80	9
5	432.21	216.60	T	1425.58	713.29	8
6	547.24	274.10	D	1324.53	662.77	7
7	646.31	323.65	V	1209.52	605.25	6
8	774.36	387.68	Q	1110.46	555.72	5
9	903.41	452.20	E	982.39	491.69	4
10	1032.45	516.72	E	853.34	427.17	3
11	1581.62	791.31	C(+446.17)	724.29	362.65	2
12			R	175.12	88.06	1

Supplementary Figure S7

[S7.52] Putative elongation factor 1-alpha-like 3

SGDAAIVDMVPGKPMC(+446.17)VESFSDYPPLGR

m/z=1128.852 z=3

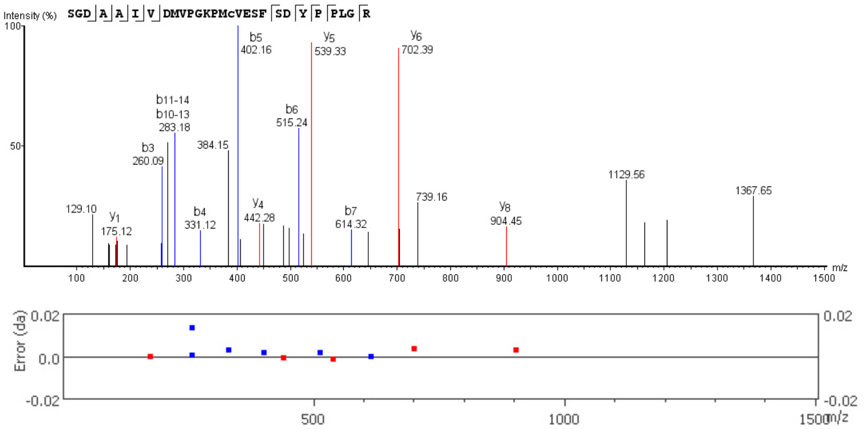

#	b	b (2+)	Seq	y	y (2+)	#
1	88.04	44.52	S			28
2	145.06	73.03	G	3297.51	1649.26	27
3	260.09	130.54	D	3240.49	1620.75	26
4	331.12	166.06	A	3125.46	1563.23	25
5	402.16	201.58	A	3054.43	1527.71	24
6	515.24	258.11	I	2983.39	1492.19	23
7	614.32	307.66	V	2870.31	1435.65	22
8	729.34	365.17	D	2771.24	1386.12	21
9	860.38	430.69	M	2656.21	1328.60	20
10	959.45	480.23	V	2525.17	1263.08	19
11	1056.50	528.75	P	2426.10	1213.55	18
12	1113.53	557.26	G	2329.05	1165.02	17
13	1241.62	621.31	K	2272.03	1136.51	16
14	1338.67	669.84	P	2143.93	1072.47	15
15	1469.71	735.36	M	2046.88	1023.94	14
16	2018.89	1009.94	C(+446.17)	1915.84	958.42	13
17	2117.96	1059.48	V	1366.66	683.83	12
18	2247.00	1124.00	E	1267.60	634.30	11
19	2334.03	1167.52	S	1138.55	569.78	10
20	2481.10	1241.05	F	1051.52	526.26	9
21	2568.13	1284.57	S	904.45	452.73	8
22	2683.16	1342.08	D	817.42	409.21	7
23	2846.22	1423.61	Y	702.39	351.70	6
24	2943.27	1472.14	P	539.33	270.16	5
25	3040.33	1520.66	P	442.28	221.64	4
26	3153.41	1577.21	L	345.22	173.11	3
27	3210.43	1605.72	G	232.14	116.57	2
28			R	175.12	88.06	1

[S7.53] Heterogeneous nuclear ribonucleoprotein K

LFQEC(+446.17)C(+414.19)PHSTDR

m/z=766.0003 z=3

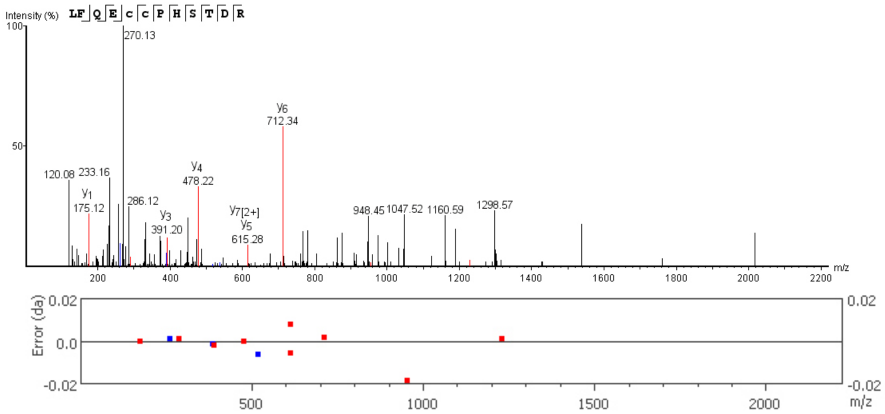

#	b	b (2+)	Seq	y	y (2+)	#
1	114.09	57.55	L			12
2	261.16	131.08	F	2182.88	1091.94	11
3	389.22	195.11	Q	2035.82	1018.41	10
4	518.27	259.63	E	1907.76	954.40	9
5	1067.44	534.22	C(+446.17)	1778.72	889.86	8
6	1584.64	792.82	C(+414.19)	1229.54	615.28	7
7	1681.69	841.35	P	712.34	356.67	6
8	1818.75	909.88	H	615.28	308.14	5
9	1905.78	953.39	S	478.22	239.61	4
10	2006.83	1003.92	T	391.20	196.10	3
11	2121.86	1061.43	D	290.14	145.57	2
12			R	175.12	88.06	1

[S7.54] Stress-induced-phosphoprotein 1

ALDLDSSC(+446.17)K

m/z=699.3172 z=2

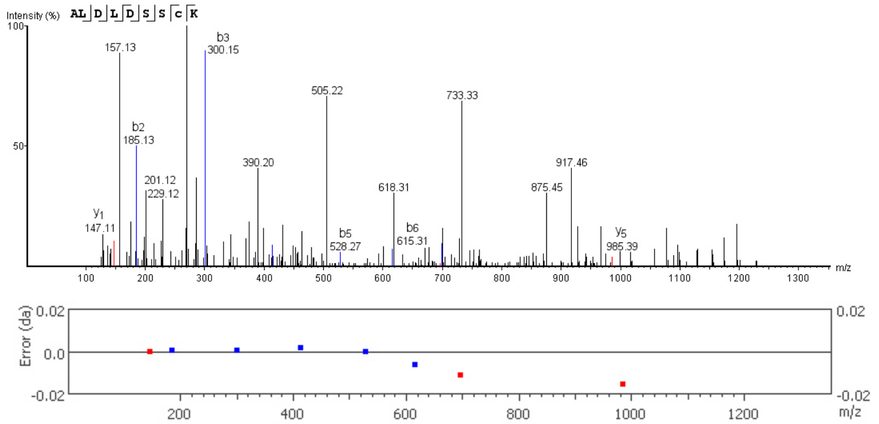

#	b	b (2+)	Seq	y	y (2+)	#
1	72.04	36.52	A			9
2	185.13	93.06	L	1326.57	663.79	8
3	300.15	150.58	D	1213.49	607.24	7
4	413.24	207.12	L	1098.46	549.73	6
5	528.27	264.63	D	985.39	493.19	5
6	615.31	308.15	S	870.35	435.68	4
7	702.33	351.67	S	783.32	392.16	3
8	1251.51	626.25	C(+446.17)	696.30	348.64	2
9			K	147.11	74.06	1

Supplementary Figure S7

[S7.55] Heterogeneous nuclear ribonucleoprotein R

SAFLC(+446.17)GVMK

m/z=701.3325z=2

#	b	b (2+)	Seq	y	y (2+)	#
1	88.04	44.52	S			9
2	159.08	80.04	A	1314.61	657.80	8
3	306.14	153.57	F	1243.57	622.30	7
4	419.23	210.11	L	1096.51	548.75	6
5	968.40	484.70	C(+446.17)	983.43	492.21	5
6	1025.43	513.21	G	434.24	217.62	4
7	1124.49	562.75	V	377.22	189.11	3
8	1255.53	628.27	M	278.15	139.58	2
9			K	147.11	74.06	1

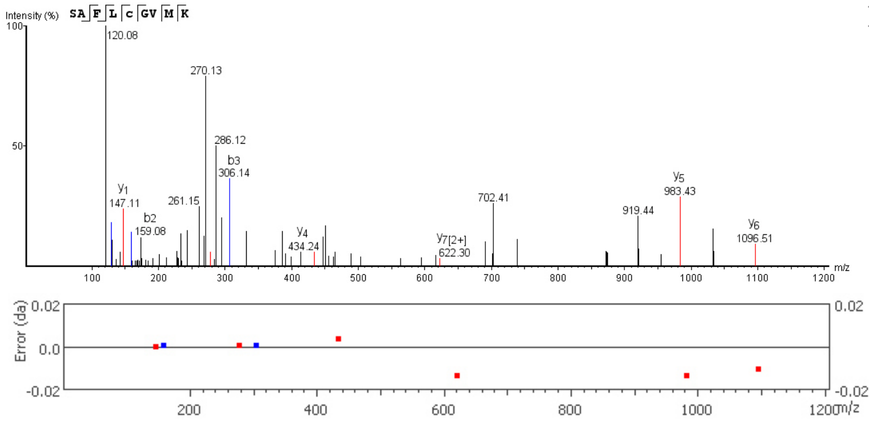

[S7.56] Peridoxin-6

DFTPVC(+446.17)TTELGR

m/z=892.9135 z=2

#	b	b (2+)	Seq	y	y (2+)	#
1	116.03	58.52	D			12
2	263.10	132.05	F	1669.77	835.39	11
3	364.15	182.58	T	1522.71	761.85	10
4	461.20	231.10	P	1421.65	711.33	9
5	560.27	280.64	V	1324.61	662.80	8
6	1109.45	555.22	C(+446.17)	1225.54	613.27	7
7	1210.49	605.75	T	676.36	338.68	6
8	1311.54	656.27	T	575.31	288.16	5
9	1440.58	720.79	E	474.27	237.63	4
10	1553.67	777.33	L	345.22	173.11	3
11	1610.69	805.85	G	232.14	116.57	2
12			R	175.12	88.06	1

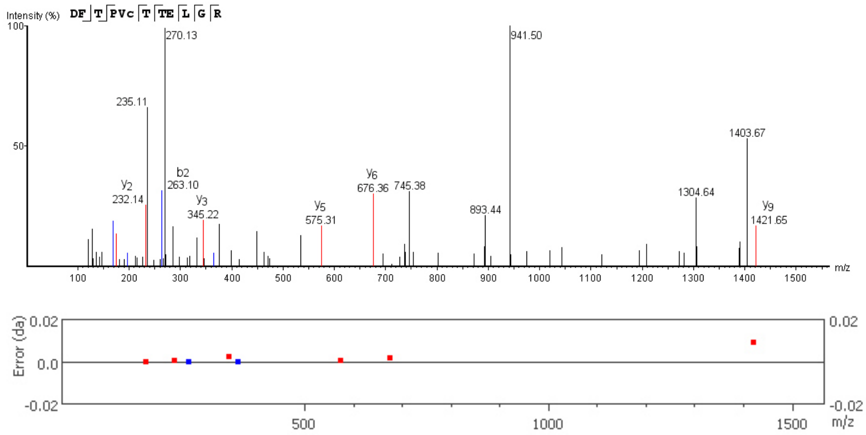

[S7.57] Adenosylhomocysteinase

FDNLYGC(+446.17)R

m/z=717.3129 z=2

#	b	b (2+)	Seq	y	y (2+)	#
1	148.08	74.54	F			8
2	263.10	132.05	D	1286.53	643.77	7
3	377.14	189.07	N	1171.51	586.25	6
4	490.23	245.62	L	1057.46	529.23	5
5	653.29	327.15	Y	944.39	472.69	4
6	710.31	355.66	G	781.32	391.16	3
7	1259.49	630.24	C(+446.17)	724.29	362.65	2
8			R	175.12	88.06	1

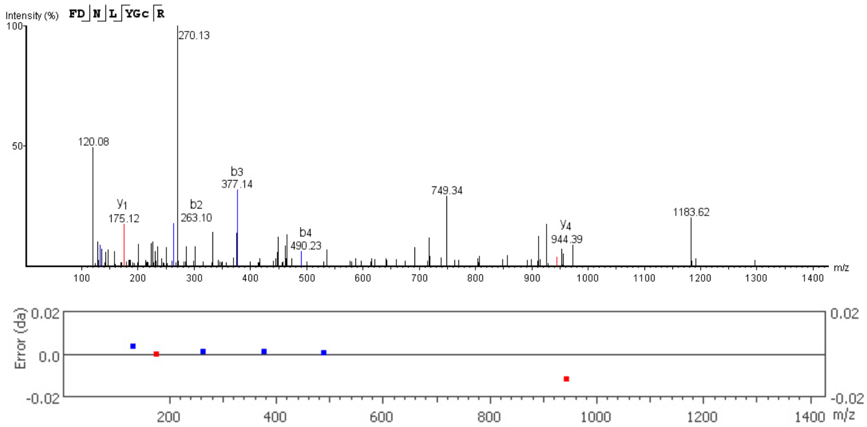

Supplementary Figure S7

[S7.58] Tropomyosin alpha-3 chain

C(+446.17)LSAAEEK

m/z=648.79333 z=2

#	b	b (2+)	Seq	y	y (2+)	#
1	550.18	275.59	C(+446.17)			8
2	663.27	332.13	L	747.39	374.19	7
3	750.30	375.65	S	634.30	317.65	6
4	821.34	411.17	A	547.27	274.14	5
5	892.37	446.69	A	476.23	238.62	4
6	1021.42	511.21	E	405.20	203.10	3
7	1150.46	575.73	E	276.16	138.58	2
8			K	147.11	74.06	1

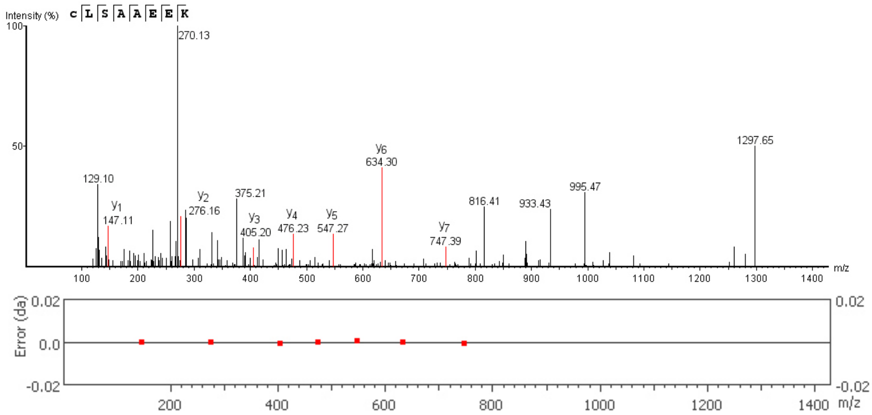

[S7.59] Plasminogen activator inhibitor 1 RNA-binding protein

PGHLQEGFGC(+446.17)VVTNR

m/z=687.326 z=3

#	b	b (2+)	Seq	y	y (2+)	#
1	98.06	49.53	P			15
2	155.08	78.04	G	1962.90	981.95	14
3	292.14	146.57	H	1905.88	953.44	13
4	405.22	203.11	L	1768.82	884.91	12
5	533.28	267.14	Q	1655.73	828.37	11
6	662.33	331.66	E	1527.68	764.34	10
7	719.35	360.17	G	1398.63	699.82	9
8	866.41	433.71	F	1341.61	671.31	8
9	923.44	462.22	G	1194.54	597.77	7
10	1472.61	736.81	C(+446.17)	1137.52	569.26	6
11	1571.68	786.34	V	588.35	294.67	5
12	1670.75	835.87	V	489.28	245.14	4
13	1771.80	886.40	T	390.21	195.60	3
14	1885.84	943.42	N	289.16	145.08	2
15			R	175.12	88.06	1

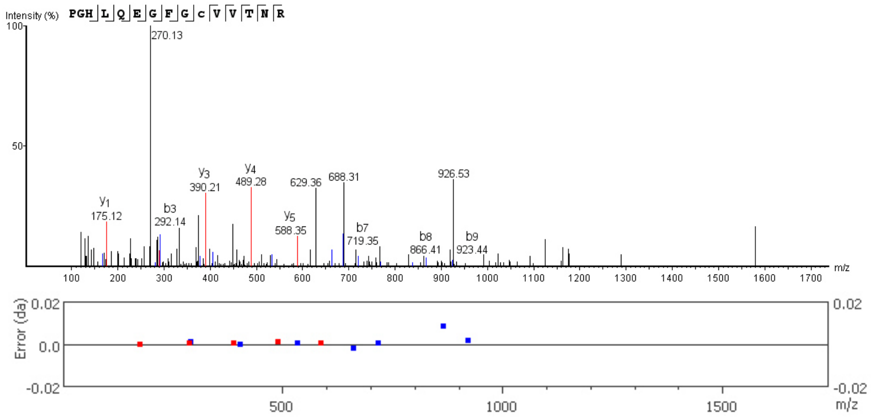

[S7.60] 40S ribosomal protein S3a

AC(+446.17)QSIYPLHDVFVR

m/z=698.67615 z=3

#	b	b (2+)	Seq	y	y (2+)	#
1	72.04	36.52	A			14
2	621.22	311.11	C(+446.17)	2022.96	1011.98	13
3	749.28	375.14	Q	1473.78	737.39	12
4	836.31	418.66	S	1345.73	673.36	11
5	949.39	475.20	I	1258.69	629.85	10
6	1112.46	556.73	Y	1145.61	573.31	9
7	1209.51	605.26	P	982.55	491.77	8
8	1322.59	661.80	L	885.49	443.25	7
9	1459.65	730.33	H	772.41	386.71	6
10	1574.68	787.84	D	635.36	318.18	5
11	1673.75	837.37	V	520.32	260.66	4
12	1820.82	910.91	F	421.26	211.14	3
13	1919.89	960.44	V	274.19	137.59	2
14			R	175.12	88.06	1

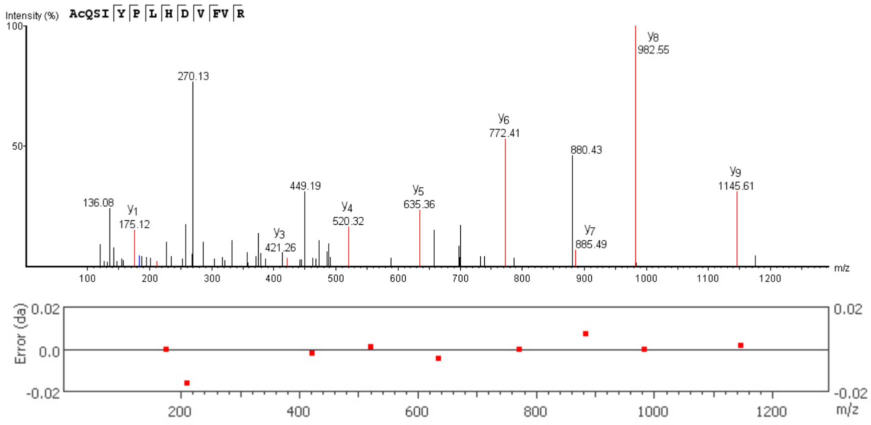

Supplementary Figure S7

[S7.61] Destrin

C(+446.17)STPEEIKK

m/z=494.2378 z=3

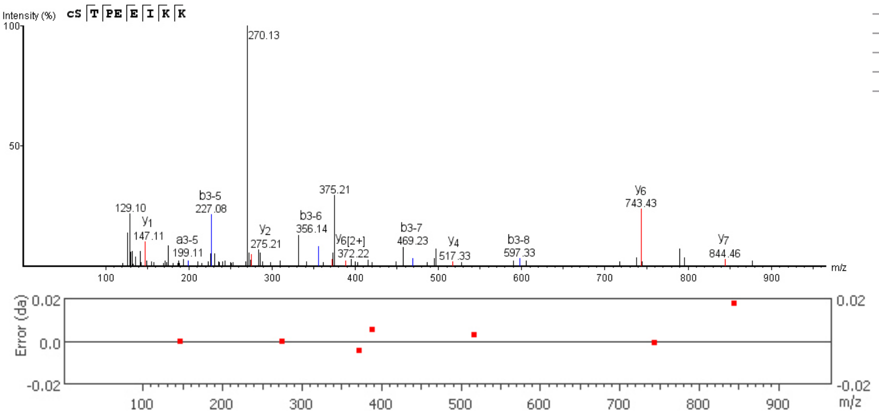

#	b	b (2+)	Seq	y	y (2+)	#
1	550.18	275.59	C(+446.17)			9
2	637.21	319.11	S	931.51	466.25	8
3	738.26	369.63	T	844.46	422.74	7
4	835.32	418.16	P	743.43	372.22	6
5	964.36	482.68	E	646.38	323.69	5
6	1093.40	547.20	E	517.33	259.17	4
7	1206.48	603.74	I	388.29	194.65	3
8	1334.58	667.79	K	275.21	138.10	2
9			K	147.11	74.06	1

[S7.62] 40S ribosomal protein S4, X isoform

FDTGNLC(+446.17)MVTGGANLGR

m/z=1086.4978 z=2

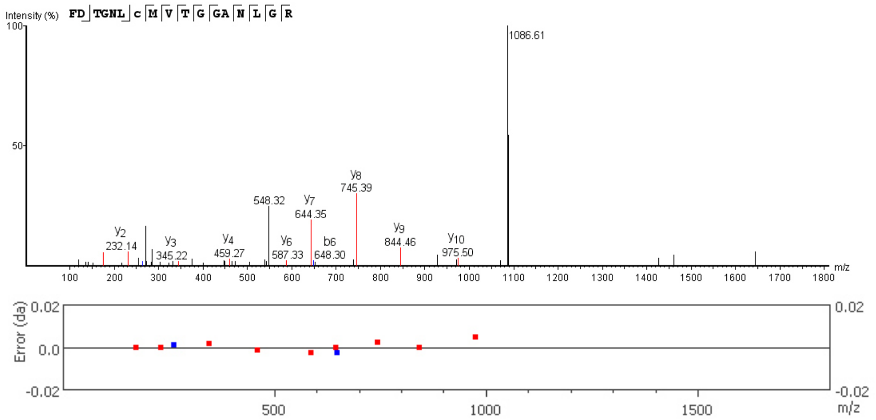

#	b	b (2+)	Seq	y	y (2+)	#
1	148.08	74.54	F			17
2	263.10	132.05	D	2024.90	1012.95	16
3	364.15	182.58	T	1909.88	955.44	15
4	421.17	211.09	G	1808.83	904.91	14
5	535.22	268.11	N	1751.81	876.40	13
6	648.30	324.65	L	1637.76	819.38	12
7	1197.47	599.24	C(+446.17)	1524.68	762.84	11
8	1328.51	664.76	M	975.50	488.25	10
9	1427.58	714.29	V	844.46	422.73	9
10	1528.63	764.82	T	745.39	373.20	8
11	1585.65	793.33	G	644.35	322.67	7
12	1642.67	821.84	G	587.33	294.16	6
13	1713.71	857.36	A	530.30	265.65	5
14	1827.75	914.38	N	459.27	230.13	4
15	1940.84	970.92	L	345.22	173.11	3
16	1997.86	999.43	G	232.14	116.57	2
17			R	175.12	88.06	1

[S7.63] Peredoxin-1

HGEVC(+446.17)PAGWKPGSDTIKPDVQK

m/z=699.5911 z=4

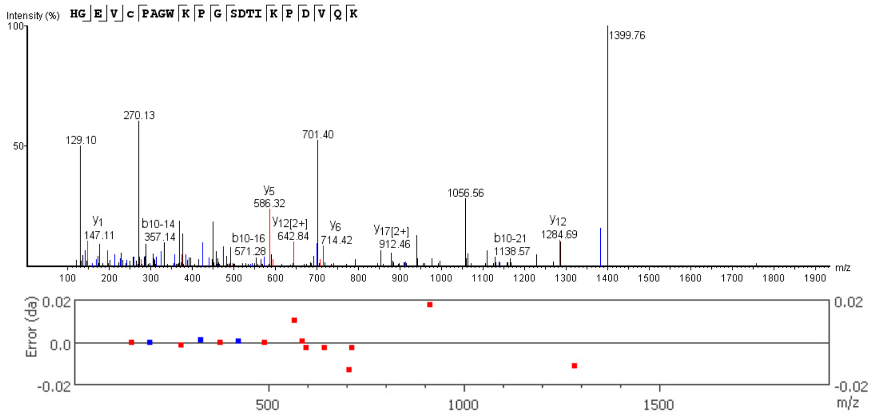

#	b	b (2+)	Seq	y	y (2+)	#
1	138.07	69.53	H			22
2	195.09	98.04	G	2658.27	1329.64	21
3	324.13	162.57	E	2601.25	1301.13	20
4	423.20	212.10	V	2472.21	1236.60	19
5	972.37	486.69	C(+446.17)	2373.14	1187.07	18
6	1069.43	535.21	P	1823.96	912.46	17
7	1140.46	570.73	A	1726.91	863.96	16
8	1197.49	599.24	G	1655.88	828.44	15
9	1383.56	692.28	W	1598.85	799.93	14
10	1511.66	756.33	K	1412.77	706.90	13
11	1608.71	804.86	P	1284.69	642.84	12
12	1665.73	833.37	G	1187.63	594.32	11
13	1752.77	876.88	S	1130.61	565.79	10
14	1867.79	934.40	D	1043.57	522.29	9
15	1968.84	984.92	T	928.55	464.77	8
16	2081.92	1041.46	I	827.50	414.25	7
17	2210.02	1105.51	K	714.42	357.71	6
18	2307.07	1154.04	P	586.32	293.66	5
19	2422.10	1211.55	D	489.27	245.13	4
20	2521.17	1261.08	V	374.24	187.62	3
21	2649.23	1325.11	Q	275.17	138.09	2
22			K	147.11	74.06	1

Supplementary Figure S7

[S7.64] Heterogeneous nuclear ribonucleoprotein U

EGC(+446.17)TEVSLLR

m/z=776.8697 z=2

#	b	b (2+)	Seq	y	y (2+)	#
1	130.05	65.53	E			10
2	187.07	94.04	G	1423.67	712.34	9
3	736.25	368.62	C(+446.17)	1366.65	683.83	8
4	837.29	419.15	T	817.48	409.24	7
5	966.34	483.67	E	716.44	358.72	6
6	1065.41	533.20	V	587.39	294.19	5
7	1152.44	576.72	S	488.32	244.66	4
8	1265.52	633.26	L	401.29	201.14	3
9	1378.61	689.80	L	288.20	144.60	2
10			R	175.12	88.06	1

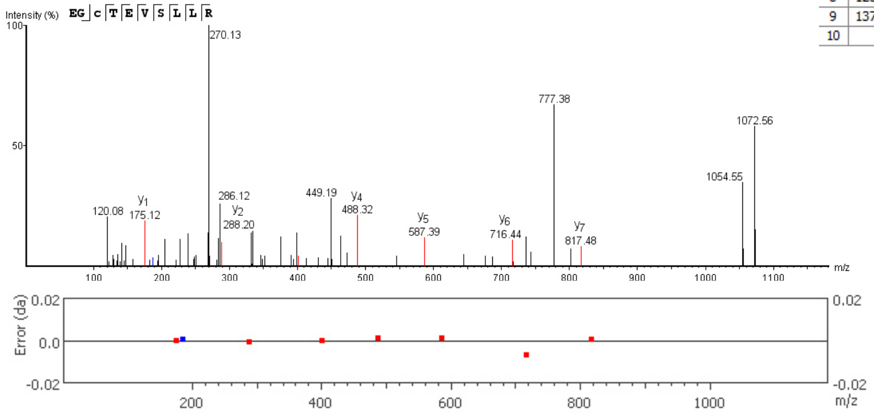

[S7.65] Protein deglycase DJ-1

DVVIC(+446.17)PDASLEDAKK

m/z=683.6673 z=3

#	b	b (2+)	Seq	y	y (2+)	#
1	116.03	58.52	D			15
2	215.10	108.05	V	1933.94	967.47	14
3	314.17	157.59	V	1834.87	917.94	13
4	427.26	214.13	I	1735.81	868.40	12
5	976.43	488.72	C(+446.17)	1622.72	811.86	11
6	1073.48	537.24	P	1073.55	537.27	10
7	1188.51	594.76	D	976.50	488.75	9
8	1259.55	630.27	A	861.47	431.23	8
9	1346.58	673.79	S	790.43	395.72	7
10	1459.66	730.34	L	703.40	352.20	6
11	1588.71	794.85	E	590.31	295.66	5
12	1703.73	852.37	D	461.27	231.14	4
13	1774.77	887.89	A	346.25	173.62	3
14	1902.87	951.93	K	275.21	138.10	2
15			K	147.11	74.06	1

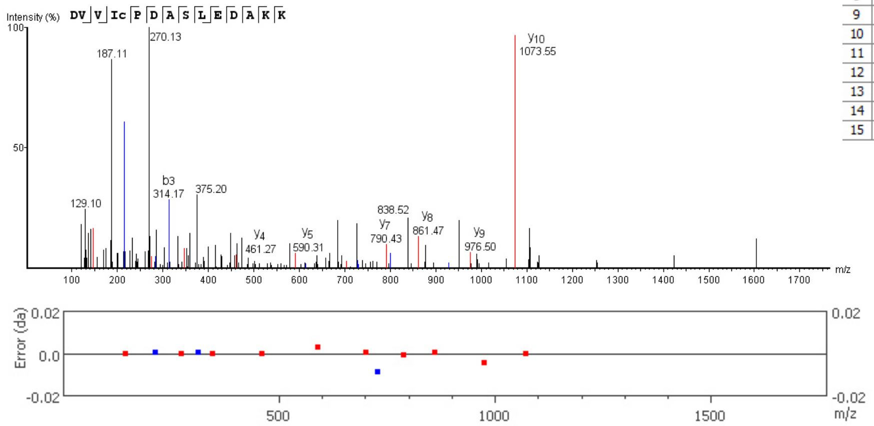

[S7.66] Programmed cell death protein 4

GTVDC(+446.17)VQAR

m/z=697.8236 z=2

#	b	a	b (2+)	Seq	y	y (2+)	#
1	58.03	30.03	29.51	G			9
2	159.08	131.08	80.04	T	1337.60	669.30	8
3	258.14	230.15	129.57	V	1236.55	618.78	7
4	373.19	345.18	187.09	D	1137.48	569.24	6
5	922.35	894.35	461.67	C(+446.17)	1022.45	511.73	5
6	1021.42	993.42	511.21	V	473.29	237.14	4
7	1149.47	1121.48	575.24	Q	374.21	187.61	3
8	1220.51	1192.51	610.76	A	246.16	123.58	2
9				R	175.12	88.06	1

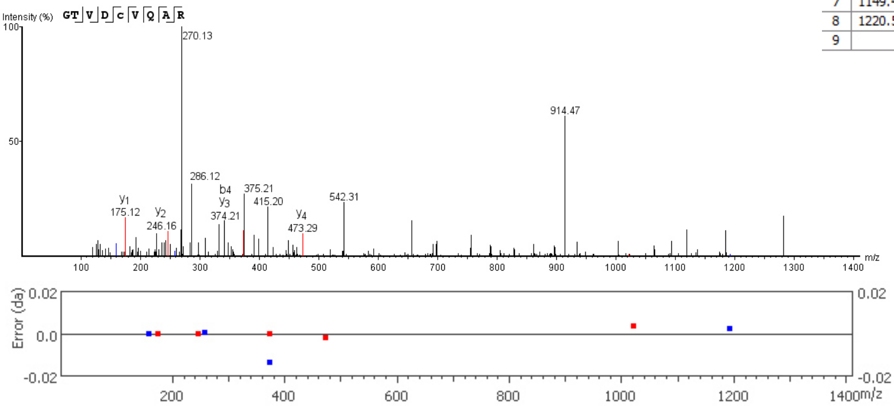

Supplementary Figure S7

[S7.67] Small nuclear ribonucleoprotein Sm D2

HC(+446.17)NMVLENVK

m/z=816.8777 z=2

#	b	b (2+)	Seq	y	y (2+)	#
1	138.07	69.53	H			10
2	687.24	344.12	C(+446.17)	1495.68	748.34	9
3	801.28	401.14	N	946.49	473.75	8
4	932.34	466.66	M	832.46	416.73	7
5	1031.39	516.20	V	701.42	351.21	6
6	1144.48	572.74	L	602.35	301.68	5
7	1273.52	637.26	E	489.27	245.13	4
8	1387.56	694.28	N	360.22	180.61	3
9	1486.63	743.82	V	246.18	123.59	2
10			K	147.11	74.06	1

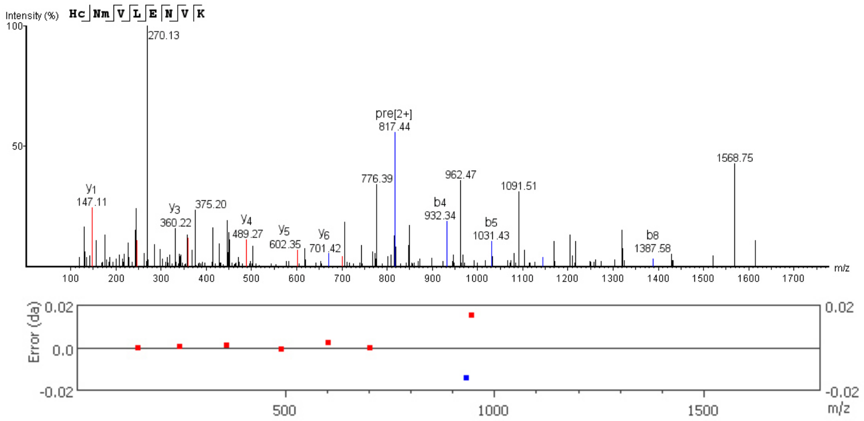

[S7.68] Heterogeneous nuclear ribonucleoprotein D0

GFC(+446.17)FITFK

m/z=704.8352 z=2

#	b	b (2+)	Seq	y	y (2+)	#
1	58.03	29.51	G			8
2	205.10	103.05	F	1351.62	676.31	7
3	754.27	377.64	C(+446.17)	1204.56	602.78	6
4	901.34	451.17	F	655.38	328.19	5
5	1014.43	507.71	I	508.31	254.66	4
6	1115.47	558.24	T	395.23	198.11	3
7	1262.54	631.77	F	294.18	147.59	2
8			K	147.11	74.06	1

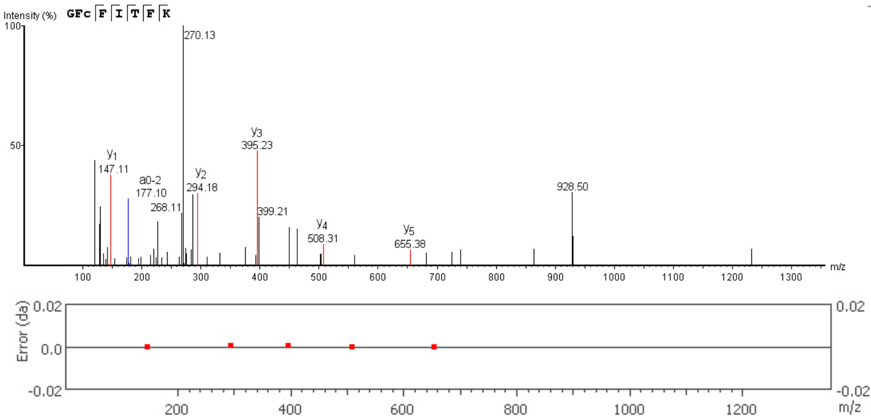

[S7.69] Glutathione S-transferase omega-1

LNEC(+446.17)VDHTPK

m/z=801.36676 z=2

#	b	b (2+)	Seq	y	y (2+)	#
1	114.09	57.55	L			10
2	228.13	114.57	N	1488.63	744.81	9
3	357.18	179.09	E	1374.59	687.79	8
4	906.35	453.68	C(+446.17)	1245.54	623.27	7
5	1005.42	503.21	V	696.36	348.68	6
6	1120.45	560.72	D	597.30	299.15	5
7	1257.51	629.25	H	482.27	241.64	4
8	1358.55	679.78	T	345.21	173.11	3
9	1455.61	728.30	P	244.17	122.58	2
10			K	147.11	74.06	1

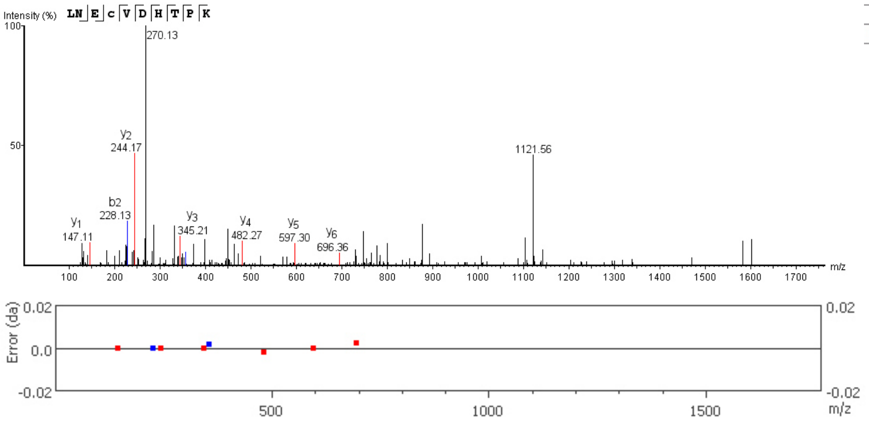

Supplementary Figure S7

[S7.70] Ras-related C3 botulinum toxin substrate 1

AVLC(+446.17)PPPVK

m/z=685.3553 z=2

#	b	b (2+)	Seq	y	y (2+)	#
1	72.04	36.52	A			9
2	171.11	86.06	V	1298.67	649.83	8
3	284.20	142.60	L	1199.60	600.30	7
4	833.37	417.19	C(+446.17)	1086.51	543.76	6
5	930.43	465.71	P	537.34	269.17	5
6	1027.48	514.24	P	440.29	220.64	4
7	1124.53	562.77	P	343.24	172.12	3
8	1223.60	612.30	V	246.18	123.59	2
9			K	147.11	74.06	1

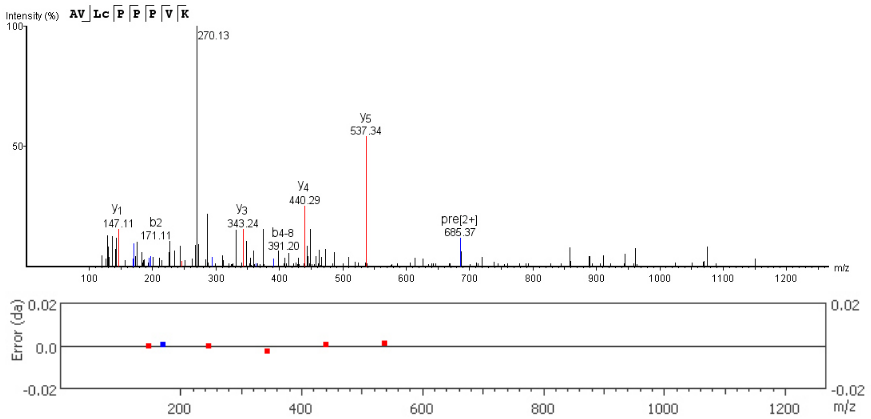

[S7.71] 60S ribosomal protein L27a

NQSFC(+446.17)PTVNLDK

m/z=906.4186 z=2

#	b	b (2+)	Seq	y	y (2+)	#
1	115.05	58.03	N			12
2	243.11	122.05	Q	1697.77	849.38	11
3	330.14	165.57	S	1569.71	785.36	10
4	477.21	239.10	F	1482.68	741.84	9
5	1026.38	513.69	C(+446.17)	1335.61	668.31	8
6	1123.44	562.22	P	786.43	393.73	7
7	1224.49	612.74	T	689.38	345.19	6
8	1323.55	662.28	V	588.34	294.67	5
9	1437.60	719.30	N	489.27	245.13	4
10	1550.68	775.84	L	375.22	188.11	3
11	1665.71	833.35	D	262.14	131.57	2
12			K	147.11	74.06	1

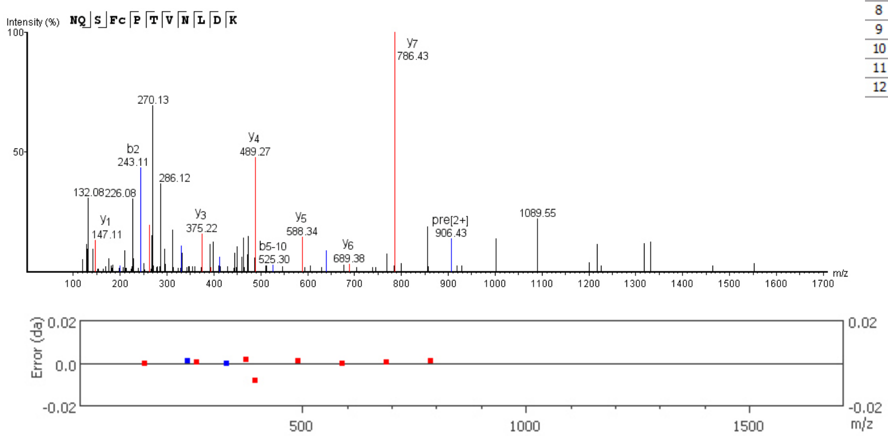

[S7.72] Histone-binding protein RBBP7

HPAKPDPSGEC(+446.17)NPDLR

m/z=727.0025 z=3

#	b	b (2+)	Seq	y	y (2+)	#
1	138.07	69.53	H			16
2	235.12	118.06	P	2041.91	1021.46	15
3	306.16	153.58	A	1944.86	972.93	14
4	434.25	217.63	K	1873.82	937.41	13
5	531.30	266.15	P	1745.73	873.36	12
6	646.34	323.67	D	1648.68	824.84	11
7	743.38	372.19	P	1533.65	767.32	10
8	830.42	415.71	S	1436.60	718.80	9
9	887.44	444.22	G	1349.56	675.28	8
10	1016.48	508.74	E	1292.54	646.77	7
11	1565.66	783.33	C(+446.17)	1163.50	582.25	6
12	1679.70	840.35	N	614.33	307.66	5
13	1776.75	888.88	P	500.28	250.64	4
14	1891.78	946.39	D	403.23	202.11	3
15	2004.86	1002.93	L	288.20	144.60	2
16			R	175.12	88.06	1

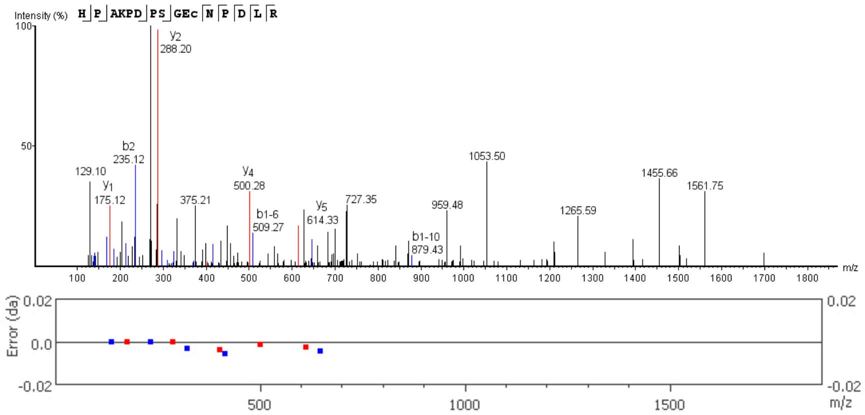

Supplementary Figure S7

[S7.73] Prostaglandin E synthase 3

LTFSC(+446.17)LGGSDNFK

m/z=917.9189 z=2

#	b	b (2+)	Seq	y	y (2+)	#
1	114.09	57.55	L			13
2	215.14	108.07	T	1721.73	861.37	12
3	362.21	181.60	F	1620.69	810.84	11
4	449.24	225.12	S	1473.62	737.31	10
5	998.41	499.71	C(+446.17)	1386.59	693.79	9
6	1111.50	556.25	L	837.41	419.21	8
7	1168.52	584.76	G	724.32	362.66	7
8	1225.54	613.27	G	667.30	334.15	6
9	1312.57	656.79	S	610.28	305.64	5
10	1427.60	714.30	D	523.25	262.13	4
11	1541.64	771.32	N	408.22	204.61	3
12	1688.71	844.86	F	294.18	147.59	2
13			K	147.11	74.06	1

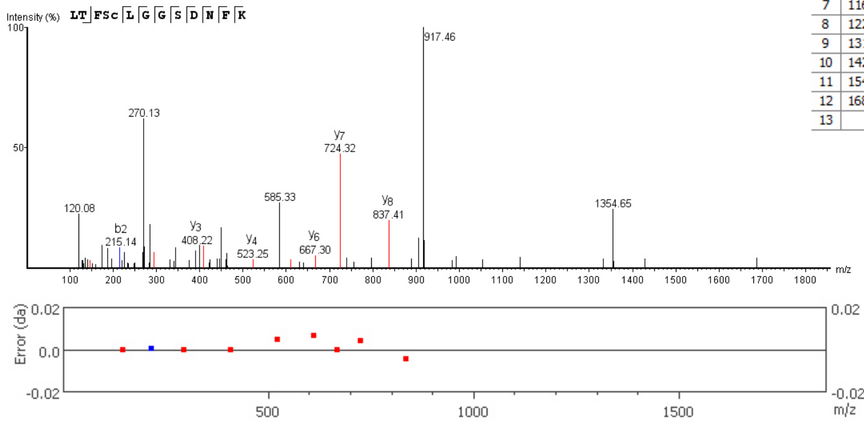

[S7.74] 60S ribosomal protein L36

EVC(+446.17)GFAPYER

m/z=808.8582 z=2

#	b	b (2+)	Seq	y	y (2+)	#
1	130.05	65.53	E			10
2	229.13	115.06	V	1487.65	744.32	9
3	778.29	389.65	C(+446.17)	1388.58	694.79	8
4	835.32	418.16	G	839.40	420.20	7
5	982.38	491.69	F	782.38	391.69	6
6	1053.42	527.21	A	635.31	318.16	5
7	1150.47	575.74	P	564.28	282.64	4
8	1313.54	657.27	Y	467.22	234.11	3
9	1442.58	721.79	E	304.16	152.58	2
10			R	175.12	88.06	1

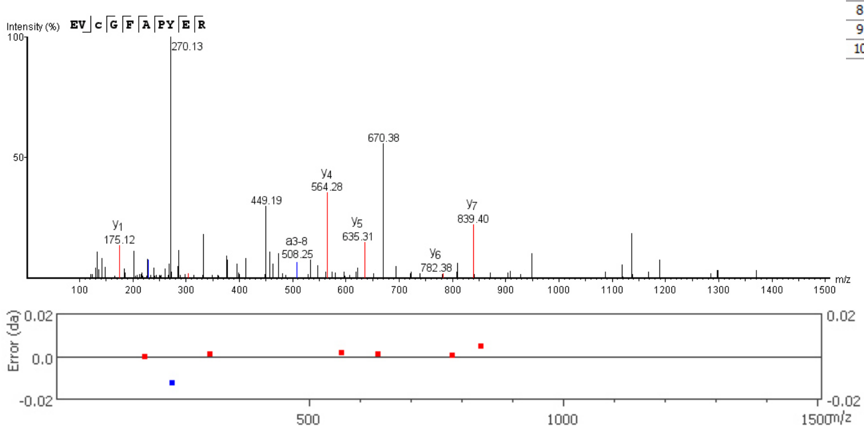

[S7.75] Nucleolin

EALNSC(+446.17)NK

m/z=662.7971 z=2

#	b	a	b (2+)	Seq	y	y (2+)	#
1	130.05	102.06	65.53	E			8
2	201.09	173.09	101.04	A	1195.53	598.26	7
3	314.17	286.18	157.59	L	1124.49	562.74	6
4	428.21	400.22	214.61	N	1011.41	506.20	5
5	515.24	487.25	258.12	S	897.36	449.19	4
6	1064.42	1036.43	532.71	C(+446.17)	810.33	405.67	3
7	1178.46	1150.47	589.73	N	261.16	131.08	2
8				K	147.11	74.06	1

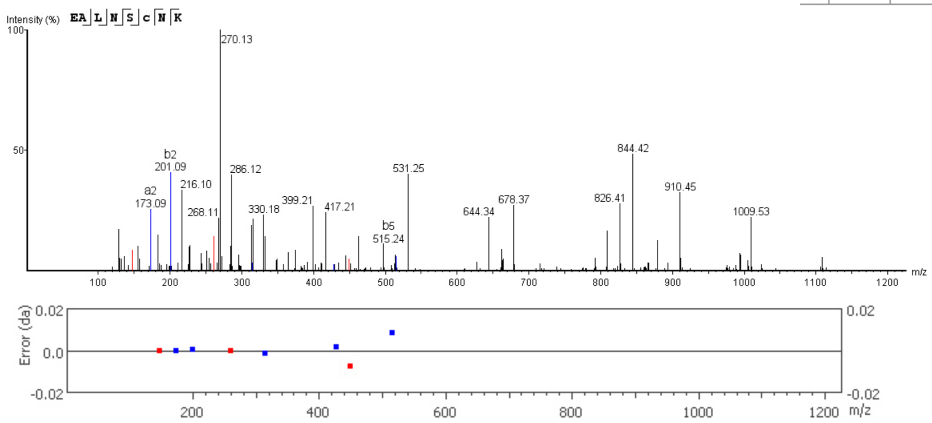

Supplementary Figure S7

[S7.76] Endoplasmin

G(+42.01)YEVIYLTEPVDEYC(+446.17)IQ(+.98)ALPEFDGK

m/z=1127.5282 z=3

#	b	a	b (2+)	Seq	y	y (2+)	#
1	100.04	72.04	50.52	G(+42.01)			25
2	263.10	235.11	132.05	Y	3281.50	1641.25	24
3	392.15	364.15	196.57	E	3118.43	1559.72	23
4	491.21	463.22	246.11	V	2989.39	1495.20	22
5	604.30	576.30	302.65	I	2890.32	1445.66	21
6	767.36	739.37	384.18	Y	2777.24	1389.12	20
7	880.45	852.45	440.72	L	2614.18	1307.59	19
8	981.49	953.50	491.25	T	2501.09	1251.05	18
9	1110.54	1082.54	555.77	E	2400.04	1200.52	17
10	1207.59	1179.59	604.29	P	2271.00	1136.00	16
11	1306.66	1278.66	653.83	V	2173.95	1087.47	15
12	1421.68	1393.69	711.34	D	2074.88	1037.94	14
13	1550.73	1522.73	775.86	E	1959.85	980.43	13
14	1713.79	1685.79	857.39	Y	1830.81	915.91	12
15	2262.96	2234.97	1131.98	C(+446.17)	1667.75	834.37	11
16	2376.05	2348.05	1188.52	I	1118.57	559.79	10
17	2505.09	2477.10	1253.05	Q(+.98)	1005.49	503.24	9
18	2576.13	2548.13	1288.56	A	876.43	438.72	8
19	2689.21	2661.22	1345.11	L	805.40	403.20	7
20	2786.27	2758.27	1393.63	P	692.32	346.66	6
21	2915.31	2887.31	1458.15	E	595.27	298.14	5
22	3062.38	3034.38	1531.69	F	466.23	233.61	4
23	3177.40	3149.41	1589.20	D	319.16	160.08	3
24	3234.42	3206.43	1617.71	G	204.13	102.57	2
25				K	147.11	74.06	1

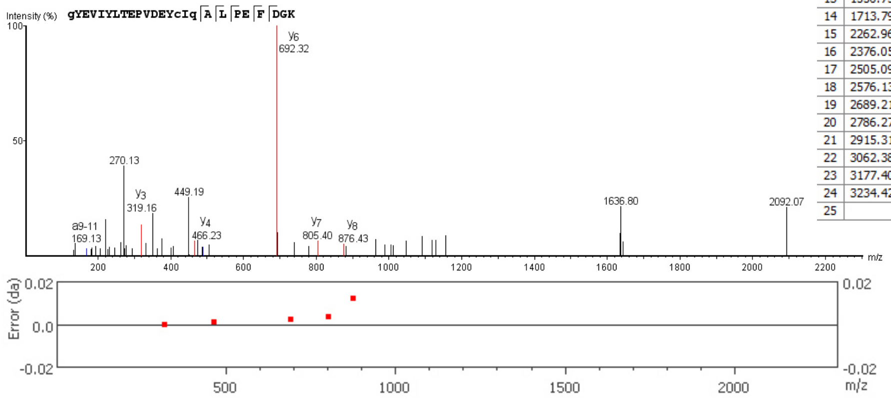

[S7.77] Peptidyl-prolyl cis-trans isomerase A

KITIADC(+446.17)GQLE

m/z=818.9002 z=2

#	b	b (2+)	Seq	y	y (2+)	#
1	129.10	65.05	K			11
2	242.19	121.59	I	1508.68	754.84	10
3	343.23	172.12	T	1395.60	698.30	9
4	456.32	228.66	I	1294.55	647.77	8
5	527.36	264.18	A	1181.46	591.23	7
6	642.38	321.69	D	1110.43	555.71	6
7	1191.56	596.28	C(+446.17)	995.40	498.20	5
8	1248.58	624.79	G	446.22	223.61	4
9	1376.64	688.82	Q	389.20	195.11	3
10	1489.72	745.36	L	261.14	131.07	2
11			E	148.06	74.53	1

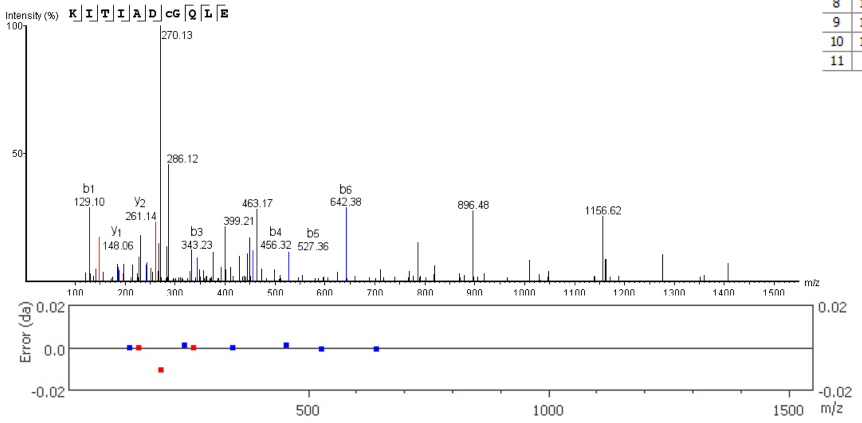

[S7.78] Heterogeneous nuclear ribonucleoprotein U

MC(+446.17)LFAGFQR

m/z=759.8464 z=2

#	b	b (2+)	Seq	y	y (2+)	#
1	132.05	66.52	M			9
2	681.22	341.11	C(+446.17)	1387.63	694.32	8
3	794.31	397.65	L	838.45	419.73	7
4	941.38	471.19	F	725.37	363.19	6
5	1012.41	506.71	A	578.31	289.65	5
6	1069.43	535.22	G	507.27	254.13	4
7	1216.50	608.75	F	450.25	225.62	3
8	1344.56	672.78	Q	303.18	152.09	2
9			R	175.12	88.06	1

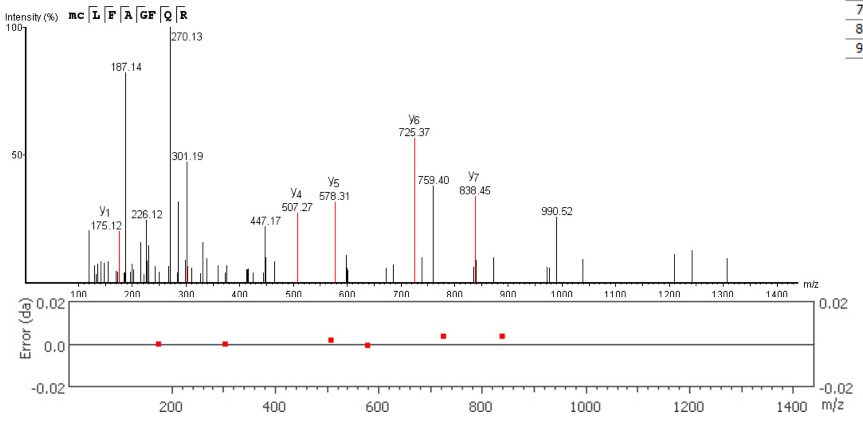

Fig S7: MS spectra of persulfide peptides (S-IAMBio, +446.17 Da) found in the total fraction of non-stimulated cells. MS/MS fragment ions of persulfide peptides identified in the total fraction of the initial proteomic approach (Fig 1) are depicted in red and blue, respectively. The inset shows the sequence coverage of the peptides by b, b^{2+}, y and y^{2+} ions. Lower panels indicate the mass error.
